# Impaired regulation by purinergic signaling axis contributes to CD8^+^ T cell dysregulation in STAT3 gain of function

**DOI:** 10.1172/jci.insight.202959

**Published:** 2026-09-08

**Authors:** Jose S. Campos Duran, Montana S. Knight, Samir U. Sayed, Megan C. Dalalo, Andrea A. Mauracher, Peyton Conrey, Aaron B. Schultz, Ceire A. Hay, Robert B. Lindell, Ilona Neale, Kyle Yeakle, Eric D. Abrams, Erica G. Schmitt, Martin A. Thelin, Christian A. Howard, Sara Bluestein, Christine M. Seroogy, Tamara C. Pozos, Akaluck Thatayatikom, Ingrid S. Lundgren, Amelie Gauthier, Scott W. Canna, Helen C. Su, Michael D. Keller, Ottavia M. Delmonte, Lisa R. Forbes Satter, Steven M. Holland, Jenna R.E. Bergerson, Jennifer W. Leiding, Neil Romberg, Will Bailis, Christopher A. Hunter, Alexandra F. Freeman, Alejandro V. Villarino, Mark S. Anderson, Megan A. Cooper, Tiphanie P. Vogel, Sarah E. Henrickson

**Affiliations:** 1 Division of Allergy and Immunology, Department of Pediatrics, Children’s Hospital of Philadelphia, Philadelphia, Pennsylvania, USA.; 2 Perelman School of Medicine, University of Pennsylvania, Philadelphia, Pennsylvania, USA.; 3 Institute for Immunology and Immune Health, University of Pennsylvania Perelman School of Medicine, Philadelphia, Pennsylvania, USA.; 4 Department of Biomedical and Health Informatics, Children’s Hospital of Philadelphia, Philadelphia, Pennsylvania, USA.; 5 Department of Microbiology and Immunology, Miller School of Medicine, and; 6 Sylvester Comprehensive Cancer Center, University of Miami, Miami, Florida, USA.; 7 Division of Critical Care Medicine, Department of Anesthesia and Critical Care, Children’s Hospital of Philadelphia, Philadelphia, Pennsylvania, USA.; 8 Department of Pediatrics, Division of Rheumatology and Immunology, and; 9 Department of Pathology and Immunology, Washington University School of Medicine, St. Louis, Missouri, USA.; 10 Diabetes Center, and; 11 Department of Pediatric Endocrinology, UCSF, San Francisco, California, USA.; 12 Atlanta Allergy & Asthma, Atlanta, Georgia, USA.; 13 University of Wisconsin School of Medicine and Public Health, Madison, Wisconsin, USA.; 14 Clinical Immunology, Children’s Minnesota, Minneapolis, Minnesota, USA.; 15 Pediatric Rheumatology/Immunology, AdventHealth for Children, Orlando, Florida, USA.; 16 Pediatric Infectious Diseases, St. Luke’s Children’s Hospital, Boise, Idaho, USA.; 17 Department of Allergy and Immunology, CHU de Québec-CHUL, Laval University Hospital Center, Laval University, Quebec City, Quebec, Canada.; 18 Division of Rheumatology, Immune Dysregulation Program, Children’s Hospital of Philadelphia, Philadelphia, Pennsylvania, USA.; 19 Laboratory of Clinical Immunology and Microbiology, National Institute of Allergy and Infectious Diseases, NIH, Bethesda, Maryland, USA.; 20 Center for Cancer & Immunology Research, and; 21 Division of Allergy and Immunology, Children’s National Hospital, Washington, DC, USA.; 22 GW Cancer Center, George Washington University School of Medicine, Washington, DC, USA.; 23 Department of Pediatrics, Division of Immunology, Allergy and Retrovirology, Baylor College of Medicine and William T Shearer Center for Human Immunobiology and Texas Children’s Research Institute, Texas Children’s Hospital, Houston, Texas, USA.; 24 Division of Allergy and Immunology, Department of Pediatrics, Johns Hopkins University, Baltimore, Maryland, USA.; 25 Institute for Clinical and Translational Research and Cancer and Blood Disorders Institute, Johns Hopkins All Children’s Hospital, St. Petersburg, Florida, USA.; 26 Department of Pathology and Laboratory Medicine, Children’s Hospital of Philadelphia, Philadelphia, Pennsylvania, USA.; 27 Department of Pathology and Laboratory Medicine, Perelman School of Medicine, University of Pennsylvania, Philadelphia, Pennsylvania, USA.; 28 Department of Pathobiology, School of Veterinary Medicine, University of Pennsylvania, Philadelphia, Pennsylvania, USA.; 29 Department of Medicine, and; 30 Diabetes Center, UCSF, San Francisco, California, USA.; 31 Department of Pediatrics, Division of Rheumatology, Baylor College of Medicine and William T Shearer Center for Human Immunobiology and Texas Children’s Research Institute, Texas Children’s Hospital, Houston, Texas, USA.; 32 Department of Microbiology, Perelman School of Medicine, University of Pennsylvania, Philadelphia, Pennsylvania, USA.

**Keywords:** Immunology, Metabolism, Genetic diseases, T cells

## Abstract

Gain-of-function (GOF) variants in STAT3 cause a complex disorder characterized by early-onset autoimmunity, lymphoproliferation, recurrent infections, and immune dysregulation. In both primary human and mouse models of STAT3 GOF, CD8^+^ T cells have been implicated as pathogenic drivers of autoimmunity, though the exact mechanisms remain poorly understood. Here, we found that in patients with STAT3 GOF, CD8^+^ T cells exist in an activated state. Functional assessment revealed that naive CD8^+^ T cells have an increased capacity for IFN-γ and TNF-α production, with type I and type II IFN transcriptional signatures. Evaluation of immunoregulatory pathways revealed dysregulation of the purinergic signaling axis in CD8^+^ T cells: CD39 was increased, whereas downstream purinergic family members, CD73 and the adenosine receptor A_2A_R, were downregulated, impairing the potential to produce or sense immunosuppressive adenosine. Evaluation of the impact of precision therapy, in the form of JAK inhibition, at a cellular and functional level revealed partial normalization of CD8^+^ T cell dysregulation in patients, including aberrant cytokine production. Our study suggests that a dysregulated purinergic signaling axis plays a key role in CD8^+^ T cell dysregulation in STAT3 GOF and may have implications for other rare monogenic immune disorders and common inflammatory disorders.

## Abstract 2

STAT3 gain-of-function drives CD8+ T cell dysfunction, in part through a dysregulated purinergic axis, which is partially restored by targeted JAK inhibitor therapy.

## Introduction

Signal integration downstream of activating and inhibitory stimuli regulates CD8^+^ T cell homeostasis, differentiation, and function. In settings where antigenic stimulation and inflammation resolve, such as vaccination or acute infection, CD8^+^ T cells differentiate into short-lived effector cells, with a subset remaining as long-lived memory CD8^+^ T cells (1). Meanwhile, in situations with persistent antigenic stimulation in the setting of chronic inflammation, such as chronic viral infections and cancer, CD8^+^ T cells may develop into dysfunctional states (2, 3). Disrupted immunosurveillance can contribute to dysregulated effector function, including loss of self-tolerance and autoimmunity. These scenarios highlight the importance of immune cell signaling regulation and the impact of signaling pathways in human health and disease. Rare, monogenic inborn errors of immunity provide insights into human immunity by altering key signaling pathways, resulting in changes in the relative proportion and/or function of cellular and humoral components of the immune system, (4, 5). In a subset of inborn errors of immunity, known as primary immune regulatory disorders, genetic variants alter the regulation of immune cell differentiation and function, leading to autoimmunity, hyperinflammation, recurrent infections, and/or lymphoproliferation (6). Primary immune regulatory disorders that alter cytokine signaling, including STAT3 gain of function (STAT3 GOF), demonstrate how changes in a key signaling pathway can have broad and complex impacts on immune function, and these insights may facilitate identification of targetable dysfunctional pathways.

JAK/STAT signaling affects the transcriptional regulation of immune cell differentiation and activation by transforming signals from cytokines and adipokines into instructions for the cell nucleus (7–9). The transcription factor STAT3 regulates diverse immune cell processes, including proliferation, differentiation, apoptosis, and inflammation, and is activated downstream of numerous cytokines, including the gp130 family (e.g., IL-6), IL-10 family, and IL-12 family (e.g., IL-27) (10, 11). STAT3 GOF is a rare monogenic, autosomal dominant primary immune regulatory disorder initially identified in 2014 in patients with early-onset severe multi-organ autoimmunity. It is caused by a diverse group of GOF variants across the *STAT3* gene, with variable impacts on STAT3 function, including increased magnitude of phosphorylation, increased transcriptional activity, and/or delayed dephosphorylation (12–17). The most recently published global cohort of patients described 191 individuals and 72 genetic variants (12–14, 16, 17). Patients with STAT3 GOF experience a heterogeneous and broad spectrum of clinical features, which include early-onset autoimmunity (e.g., cytopenias, enteropathy, interstitial lung disease, cutaneous inflammation, and arthritis), lymphoproliferation, growth failure, and/or increased susceptibility to infections including viral infections (16–18). Understanding the mechanisms underlying altered immune function in STAT3 GOF will improve our understanding of targeted therapies, guide evaluation of response, potentially identify novel therapeutic targets, and deepen our understanding of this key pathway.

Given the early-onset autoimmunity experienced by patients with STAT3 GOF and the importance of STAT3 in Th17 and Treg differentiation (19–24), initial investigations into STAT3 GOF focused on CD4^+^ T cells (25, 26). Although some patients have increased Th17 frequencies and decreased Treg frequencies, this had been variable (16, 24, 27, 28). Research in 2 mouse models of STAT3 GOF (STAT3^+/G421R^ and STAT3^+/K392R^) on the C57BL/6 background has shown normal Treg development, differentiation, phenotype, and suppressive function, suggesting Tregs may not be the main drivers of disease (25, 26). Instead, in 2 mouse models of STAT3 GOF, CD8^+^ T cells have been implicated as drivers of autoimmunity (29, 30). The first was based on the finding that a patient with STAT3 GOF (p.K392R) developed neonatal onset type 1 diabetes (T1D) (12). STAT3 knockin mice bearing the p.K392R variant were generated on both C57BL/6 and NOD backgrounds. In the NOD background, STAT3^+/K392R^ mice demonstrated more rapid onset and higher prevalence of T1D (25, 29). Restricting expression of the STAT3 GOF variant to islet-specific TCR transgenic CD8^+^ T cells by crossing 8.3 NOD mice to STAT3^+/K392R^ mice, followed by adoptive transfer into NOD.SCID recipients, was sufficient to increase the prevalence of T1D compared with transfer of 8.3 TCR transgenic CD8^+^ T cells with WT STAT3 (29). In a separate study, pathogenic effector CD8^+^ T cells contributed to autoimmunity and mortality in mice homozygous for the STAT3 GOF variant (30). Mouse models of STAT3 GOF thus highlight the link between inflammation and autoimmunity, the dysregulation of effector CD8^+^ T cell differentiation and function, and the need to define immune dysregulatory mechanisms.

Here, we investigated how changes in STAT3 signaling lead to dysregulation of CD8^+^ T cells at a phenotypic, transcriptional, and functional level in patients with STAT3 GOF and the impact of targeted therapy on immune function. In human and mouse models of STAT3 GOF, CD8^+^ T cells were dysregulated in their immune phenotype and transcriptional profiles, including increased capacity for proinflammatory cytokine production (IFN-γ and TNF-α) and significantly increased type I and II IFN gene set expression. We identified dysregulation of the immunoregulatory purinergic signaling pathway in patients with STAT3 GOF: although there was increased CD39 on CD8^+^ T cells from patients with STAT3 GOF, expression of downstream pathway members CD73 and the adenosine receptor A_2A_R were decreased, suggesting impaired immunoregulatory function of this pathway. Through a series of in vitro assays using samples from healthy controls and patients with STAT3 GOF, along with mouse models of STAT3 GOF, we investigated the role of STAT signaling in regulating purinergic pathway function in CD8^+^ T cells. Lastly, because patients with STAT3 GOF are often treated with immunomodulatory therapy that directly inhibits upstream components of the JAK/STAT signaling pathway (e.g., JAK inhibitors with or without anti (α)-IL-6R, tocilizumab) (16, 17, 31, 32), we evaluated changes in the CD8^+^ T cell compartment in patients with STAT3 GOF who were receiving JAK inhibition (JAKi) with or without α-IL-6R. Treatment corrected components of the dysregulated CD8^+^ T cell phenotype, including purinergic signaling pathway members, and normalized or led to deficiency of inflammatory cytokine production. Together, these data suggest that dysregulation of the purinergic axis contributes to CD8^+^ T cell dysfunction in STAT3 GOF. This may suggest future strategies for targeted therapy in this rare disease, and perhaps in more common inflammatory disorders that share alterations in these regulatory pathways.

## Results

### The CD8^+^ T cell compartment is dysregulated in patients with STAT3 GOF.

**Figure 1:**
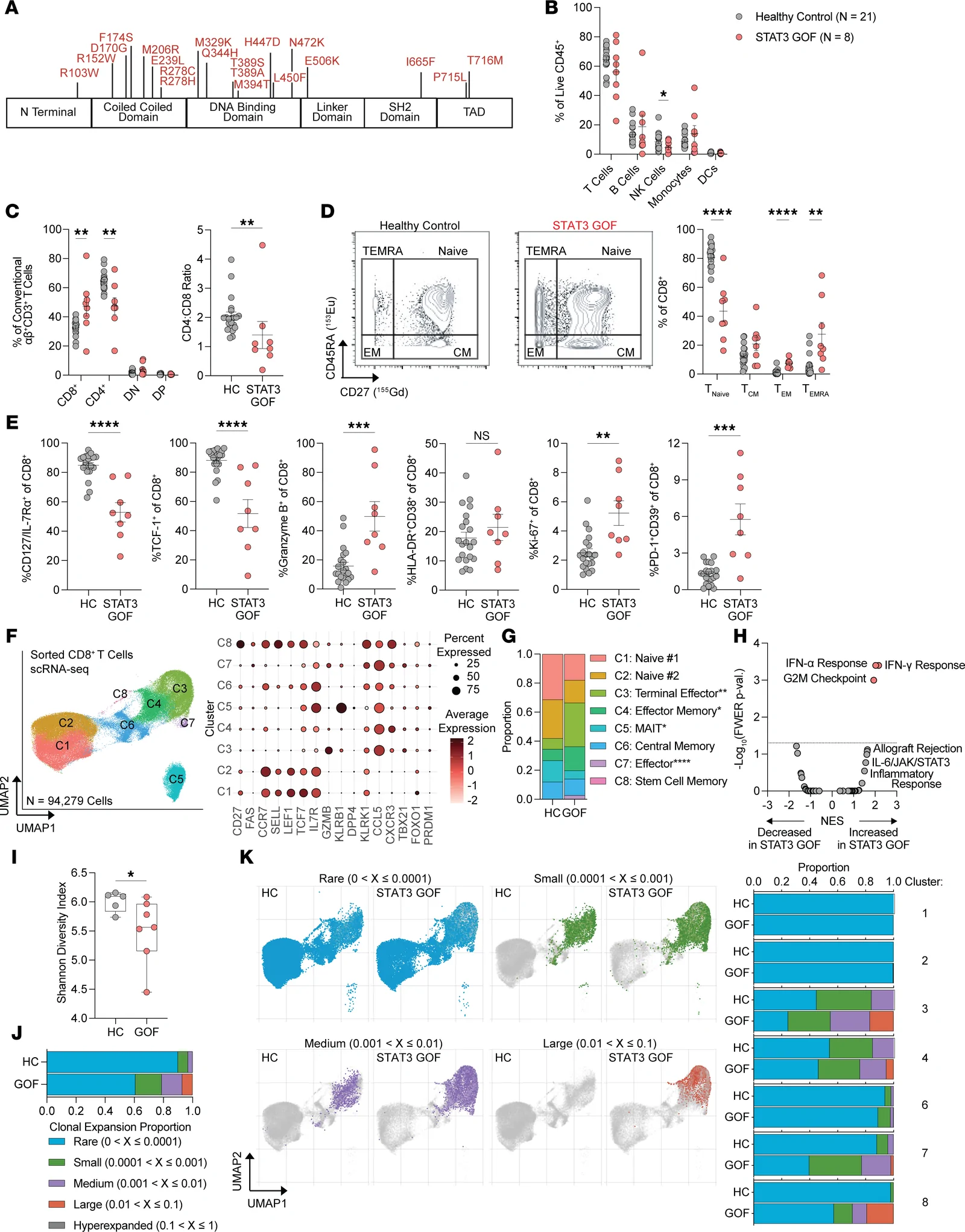
Patients with STAT3 GOF demonstrate a hyperactivated CD8^+^ T cell phenotype and transcriptional profile. (**A**) STAT3 protein domains with GOF variants indicated. (**B**) Frequencies of immune cell lineages in 8 untreated patients with STAT3 GOF and 21 age-matched healthy controls. (**C**) Frequencies of CD3^+^αβ^+^ T cell subsets after exclusion of NKT (CD3^+^CD56^+^), CD3^+^TCRγδ^+^, and MAIT (CD3^+^CD26^+^CD161^+^) cells; quantification of CD4/CD8 ratio. (**D**) Differentiation status of CD8^+^ T cells: naive, CD45RA^+^CD27^+^; central memory (CM), CD45RA^–^CD27^+^; effector memory (EM), CD45RA^–^CD27^–^; EM reexpressing CD45RA (EMRA), CD45RA^+^CD27^–^. (**E**) Activation and effector molecule expression on CD8^+^ T cells. (**F**) scRNA-Seq uniform manifold approximation and projection (UMAP) and cluster marker expression/annotation and (**G**) cluster proportion of sorted CD8^+^ T cells from 7 patients with STAT3 GOF and 5 age-matched healthy controls. (**H**) GSEA of pathways enriched in conventional CD8^+^ T cells. (**I**) Shannon Diversity Index and (**J**) proportion of expanded clones in patients with STAT3 GOF compared with healthy controls. (**K**) Visualization of TCR clonotypes overlaid on UMAPs and proportion of expanded TCR clones by cluster. For **B**–**E**: data are pooled from 3 independent experiments. Data represent mean ± SEM. *P ≤ 0.05, **P ≤ 0.01, ***P ≤ 0.001, ****P ≤ 0.0001 by Mann-Whitney test. For **G**: *P ≤ 0.05, **P ≤ 0.01, ****P ≤ 0.0001 by *propeller* (2-tailed moderated *t* test). Not listed or ns was not statistically significant. Figure 1A was created in BioRender (https://BioRender.com/4pf5knc).

To investigate the effect of increased STAT3 signaling in vivo, we began by evaluating phenotypic changes in immune cells in patients with STAT3 GOF (Figure 1A and Supplemental Table 1; supplemental material available online with this article; https://doi.org/10.1172/jci.insight.202959DS1). PBMCs from a cohort of 8 patients with STAT3 GOF not receiving immune modulatory therapy (“untreated”) along with 21 age-matched healthy controls were profiled using 43-parameter mass cytometry (Supplemental Figure 1A). As expected, the proportion of T cells, B cells, monocytes, and DCs did not significantly differ between patients with STAT3 GOF and healthy controls, though the proportion of NK cells was slightly decreased in patients (Figure 1B) (13, 14, 26, 33, 34). Within CD3^+^ cells, we did not detect any differences in the proportion of nonconventional T cells, including NKT (CD3^+^CD56^+^), CD3^+^TCRγδ^+^, or MAIT (CD3^+^CD26^+^CD161^+^) cells between patients and healthy controls (Supplemental Figure 1B). Previous studies have documented variable impact on the proportion of CD127^lo^FOXP3^+^ Tregs; here, we observe a trend toward a decreased proportion of Tregs (Supplemental Figure 1C) and CD27^+^ memory B cells in STAT3 GOF (Supplemental Figure 1D) (13, 14, 26, 33, 34). We also found an increased relative proportion of early (CD56^Bright^CD16^–^) NK cells (Supplemental Figure 1E), a decreased proportion of nonclassical (HLA-DR^+^CD16^+^CD14^–^) monocytes, and an increased proportion of classical (HLA-DR^+^CD16^–^CD14^+^) monocytes (Supplemental Figure 1F) (35).

Given that STAT3 is a critical regulator of CD8^+^ T cell differentiation and activation in cancer and viral infections (36, 37), and given the evidence of altered CD8^+^ T cell phenotype and function in mouse models of STAT3 GOF, we next defined the differentiation, activation state, and functional capacity of CD8^+^ T cells from patients with STAT3 GOF (25, 26, 29, 30, 38). Within the conventional αβ T cell compartment, we found an increased frequency of CD8^+^ T cells in STAT3 GOF compared with healthy controls, leading to a decreased CD4/CD8 ratio (Figure 1C). Moreover, subset analysis revealed a significant decrease in naive CD8^+^ T cells (CD45RA^+^CD27^+^) and an increased frequency of effector memory (T_EM_, CD45RA^–^CD27^–^) and effector memory cells reexpressing CD45RA (T_EMRA_, CD45RA^+^CD27^–^) CD8^+^ T cells, with no change in central memory cells (T_CM_, CD45RA^–^CD27^+^) (Figure 1D). Beyond differentiation, we also assessed the activation state of CD8^+^ T cells and found downregulation of memory markers, including TCF-1 and CD127 (IL-7Rα), and an increased frequency of expression of activation markers and effector molecules including Granzyme B and Ki-67, the latter indicative of a proliferative population (Figure 1E). Although there were no differences in the frequency of HLA-DR^+^CD38^+^ CD8^+^ T cells, which can be elevated in hyperinflammatory disorders or recent antigen exposure, there was an increased frequency of PD-1^+^CD39^+^ cells, which can be seen in highly activated and/or dysfunctional CD8^+^ T cells (Figure 1E) (39–43). Activation state was further evaluated through differentiation stages, with a decreased frequency of both naive and non-naive CD8^+^ T cells (particularly T_CM_) expressing CD127 and TCF-1 and increased frequency of T_CM_ cells expressing Granzyme B or PD-1^+^CD39^+^ (Supplemental Figure 1G). Given the altered differentiation and dysregulated activation state of CD8^+^ T cells in patients with STAT3 GOF, we defined their transcriptional state.

### Transcriptional and clonal dysregulation in STAT3 GOF CD8^+^ T cells.

To explore transcriptional changes in patients with STAT3 GOF, we next performed single-cell RNA-Seq (scRNA-Seq) on sorted CD8^+^ T cells from 7 patients with STAT3 GOF (6 not receiving any immunomodulatory therapy and 1 receiving targeted therapy with JAKi) and 5 age-matched healthy controls. After performing integration, clustering, and annotation, we identified 8 clusters of CD8^+^ T cells (Figure 1F and Supplemental Methods) (44–46). Our analysis demonstrated trends toward a reduction in the frequency of both naive CD8^+^ T cell clusters (C1 and C2, *P* = 0.07 and 0.12, respectively) in patients with STAT3 GOF (Figure 1, F and G) (47). There was a significant expansion of the terminal effector (C3, *P* = 0.0015), effector memory (C4, *P* = 0.019), and effector (C7, *P* = 3.7 × 10^–6^) clusters in STAT3 GOF (Figure 1, F and G). Central memory (C6) and stem cell memory (C8) clusters did not differ between healthy controls and STAT3 GOF (Figure 1, F and G). The MAIT cell cluster (C5) was decreased in STAT3 GOF (*P* = 0.013) (Figure 1, F and G). Using a multimodal approach within this unique cohort, we defined altered CD8^+^ T cell differentiation in STAT3 GOF, with an increase in the proportions of terminal effector, effector memory, and effector T cells.

Defining the mechanism by which STAT3 GOF affects CD8^+^ T cell differentiation and activation required further evaluation of transcriptional networks within these T cell states. Differentially expressed gene (DEG) analysis within conventional CD8^+^ T cells (i.e., excluding C5, MAIT cells) revealed upregulation of 554 genes, including IFN-stimulated genes (e.g., *IFIT2*, *IFIT3*, *OASL*) and genes associated with cytotoxic and effector function (e.g., *TBX21*, *CCL5*, *CXCR3*, *NKG7*); 600 genes were downregulated, including *CD27*, *SELL*, *TCF7*, and *IL7R* (Supplemental Figure 2, A and B, and Supplemental Table 2). To better understand how these gene expression changes altered the biological state and function of CD8^+^ T cells, we performed gene set enrichment analysis (GSEA) using the Hallmark Human MSigDB collection (48, 49). IFN-γ response (normalized enrichment score [NES] = 2.17, family-wise error rate [FWER] *P* < 0.0001), IFN-α response (NES = 2.02, FWER *P* < 0.0001), and G2M checkpoint (NES = 1.95, FWER *P* = 0.001) were significantly enriched in patients with STAT3 GOF, providing transcriptional evidence of IFN exposure and impact on cell cycle progression (Figure 1H, Supplemental Figure 2C, and Supplemental Table 3). Having observed changes in differentiation and evidence of altered transcription of IFN gene sets, we next assessed clonality in CD8^+^ T cells. TCR repertoire analysis revealed a decreased Shannon Diversity Index in CD8^+^ T cells from patients with STAT3 GOF, with small, medium, and large expanded clones increased in proportion (Figure 1, I and J). Expansion of these medium and large clonotypes was most pronounced within the terminal effector (C3), effector memory (C4), effector (C7), and stem cell memory (C8) clusters (Figure 1K). Large clones all represented 3 clones from 1 patient with STAT3 GOF (Supplemental Figure 2D). Overall, we observed reduced CD8^+^ T cell clonal diversity in patients with STAT3 GOF.

### STAT3 GOF CD8^+^ T cells demonstrate aberrant cytokine production profiles.

**Figure 2:**
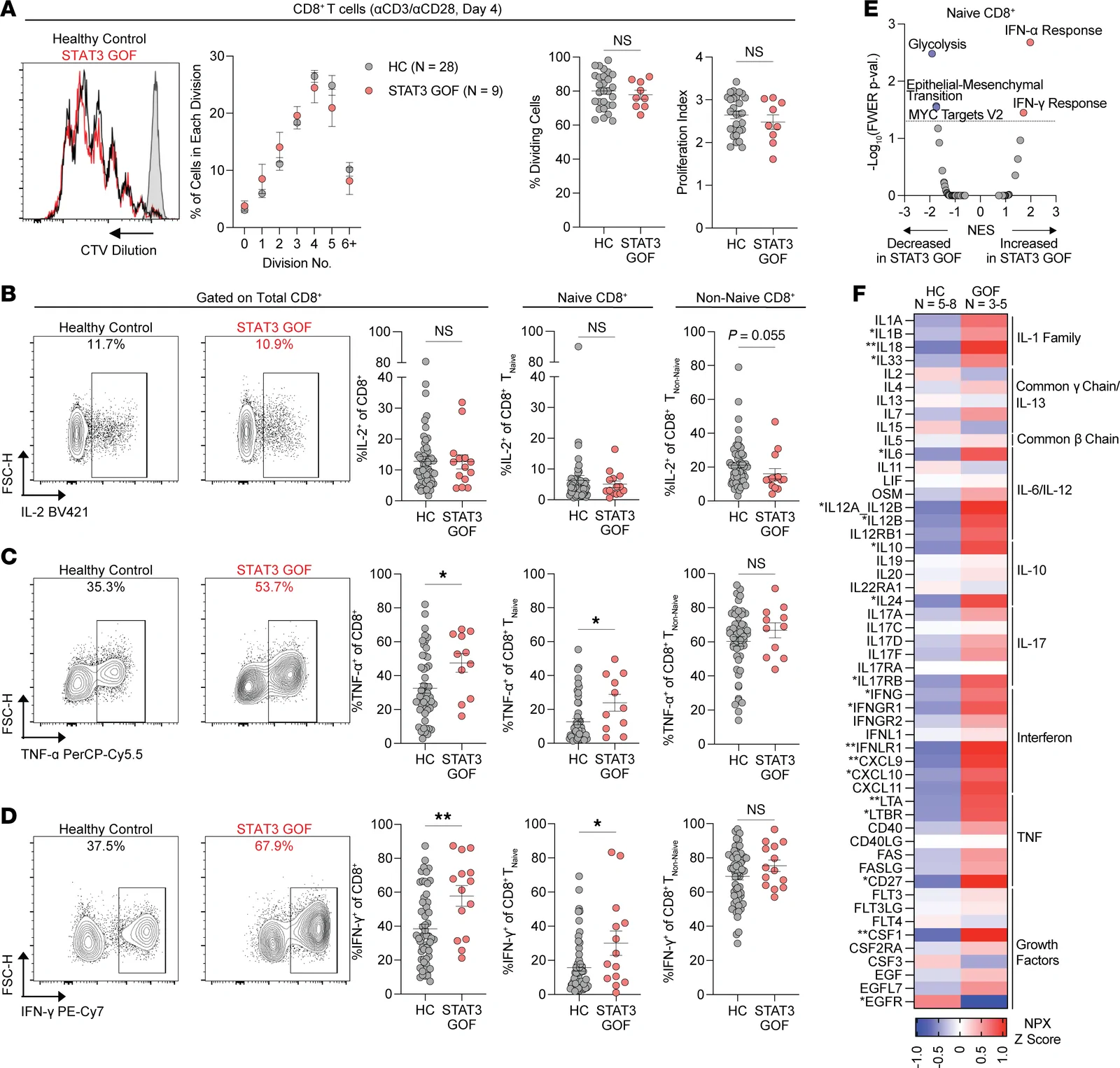
STAT3 GOF differentially affects effector function in a subset-specific manner in CD8^+^ T cells. (**A**) Representative CD8^+^ T cell CellTrace Violet dye dilution histograms from a healthy control and patient with STAT3 GOF activated with αCD3/αCD28 for 4 days. Quantification of (**B**) IL-2, (**C**) TNF-α, and (**D**) IFN-γ cytokine production within total, naive (CD45RA^+^CD27^+^), and non-naive (antigen-experienced or non-CD45RA^+^CD27^+^) CD8^+^ T cells after stimulation with PMA/ionomycin plus BFA/monensin for 4 hours. (**E**) GSEA of pathways enriched in naive CD8^+^ T cells (cluster 1 and 2). (**F**) Heatmap showing row log_2_ normalized protein expression (NPX) of select biomarkers from heparinized plasma from healthy controls (*n* = 5–8) and patients with STAT3 GOF (*n* = 3–5). For **A**: data are pooled from 3 independent experiments with healthy controls (*n* = 28) and untreated patients with STAT3 GOF (*n* = 9); for **B**–**D**: data are pooled from 7 to 8 independent experiments with healthy controls (*n* = 50–58) and untreated patients with STAT3 GOF (*n* = 11–14). Data represent mean ± SEM. *P ≤ 0.05, **P ≤ 0.01 by Mann-Whitney test.

Given the alterations in the immune phenotype and transcriptional profile of CD8^+^ T cells in STAT3 GOF, we next investigated whether these changes translated into functional impacts. Since the frequency of Ki-67^+^CD8^+^ T cells and G2M checkpoint gene set expression were increased in patients with STAT3 GOF, we first tested the ability of CD8^+^ T cells to proliferate in vitro. After 4 days of activation with αCD3/αCD28, we found no differences in either the proportion of divided cells or proliferation index, suggesting comparable rates of cell division (Figure 2A). We also tested the capacity of CD8^+^ T cells to produce cytokines. Compared with healthy controls, there were no differences in IL-2 production by total or naive (CD45RA^+^CD27^+^) CD8^+^ T cells from patients with STAT3 GOF (Figure 2B and Supplemental Figure 3A). However, there was a trending decrease in IL-2 production by non-naive (non-CD45RA^+^CD27^+^) CD8^+^ T cells (*P* = 0.055; Figure 2B). This deficit in IL-2 production was also observed within naive and non-naive CD4^+^ T cells (Supplemental Figure 3, B–D). Consistent with previous findings in humans and mouse models of STAT3 GOF, in our patient cohort, we observed an increased frequency of CD4^+^IFN-γ^+^ cells (Supplemental Figure 3B) (25, 26, 38). The proportion of IFN-γ^+^ cells was higher in total and non-naive CD4^+^ T cells in STAT3 GOF, but naive CD4^+^ T cells also had a higher proportion of IFN-γ^+^ cells (Supplemental Figure 3, B–D). TNF-α and IFN-γ production were increased in total CD8^+^ T cells in patients with STAT3 GOF (Figure 2, C and D). Subset analysis revealed that naive CD8^+^ T cells contained increased proportions of TNF-α– and IFN-γ–producing cells when stimulated (Figure 2, C and D). However, frequencies of non-naive STAT3 GOF CD8^+^ T cells producing TNF-α and IFN-γ in response to stimulation were comparable to healthy controls (Figure 2, C and D). In the previously described STAT3^+/G421R^ and NOD-STAT3^+/K392R^ mouse models of STAT3 GOF (26, 29), mice develop splenomegaly (Supplemental Figure 4A) and immune dysregulation, as indicated in part by altered CD8^+^ T cell frequency and differentiation (Supplemental Figure 4, B and C), consistent with our findings in humans. Detailed immunophenotyping of CD8^+^ T cells in both models also revealed increased expression of activation markers, including increased frequency of PD-1– and Ki-67–expressing cells and T-bet/Granzyme B coexpressing cells (Supplemental Figure 4, D and E). Moreover, we observed increased effector cytokine production, including IFN-γ and TNF-α (Supplemental Figure 4F). Thus, CD8^+^ T cells from both species showed evidence of altered function.

Having identified increased inflammatory cytokine production by naive CD8^+^ T cells in patients with STAT3 GOF, we used our scRNA-Seq data and performed GSEA within the naive CD8^+^ T cell clusters. Naive clusters (C1 and C2) demonstrated enrichment of the IFN-α and IFN-γ response gene sets, similar to our analysis within total CD8^+^ T cells (Figure 2E). To connect transcriptional alterations to the immune cell environment, we performed plasma proteomic analysis of patients with STAT3 GOF and healthy controls (50). This demonstrated increased IL-1β, IL-6, IL-10, IL-18, IFN-γ, CXCL9, and CXCL10 (the latter 2 induced by IFNs) in STAT3 GOF (Figure 2F) (37, 51–53). Overall, patients with STAT3 GOF demonstrated increased levels of systemic inflammatory cytokines, with CD8^+^ T cells exhibiting a transcriptional imprint of IFN exposure and a greater proportion of cells able to produce inflammatory cytokines. Since early-onset autoimmunity is frequently found in patients with STAT3 GOF, with evidence of CD8^+^ T cells directly contributing to lymphoproliferation and autoimmunity in mouse models (25, 29, 30), we next sought to define potential underlying mechanisms of dysregulated CD8^+^ T cell function.

### Dysregulation of purinergic pathway in CD8^+^ T cells from patients with STAT3 GOF.

**Figure 3:**
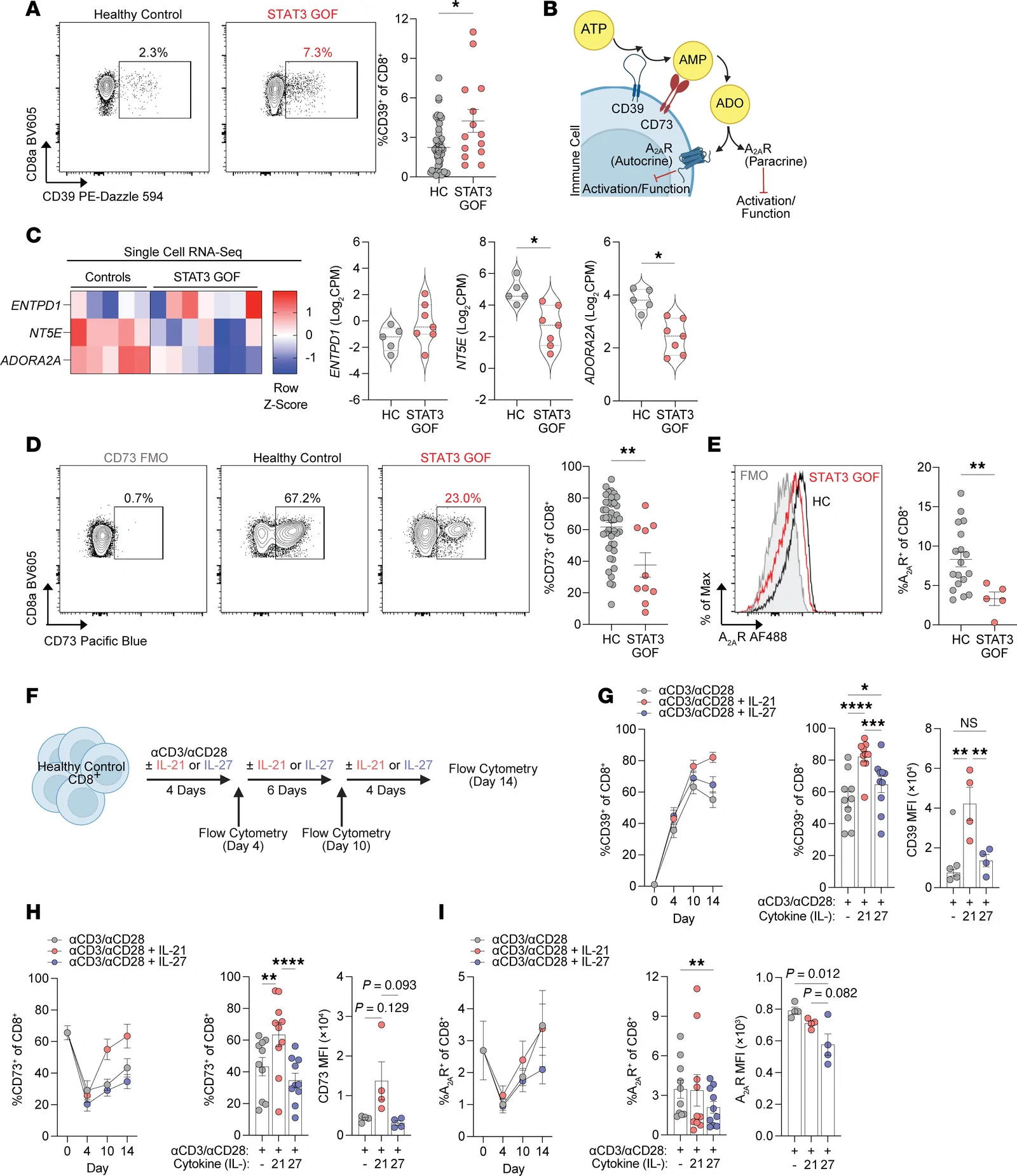
Dysregulation of purinergic pathway in patients with STAT3 GOF. (**A**) Flow cytometry plot and quantification of CD39 frequency within CD8^+^ T cells in healthy controls (*n* = 47) and patients with STAT3 GOF (*n* = 14). (**B**) Schematic of purinergic signaling regulation of immune cell activation and function. (**C**) CD39 (*ENTPD1*), CD73 (*NT5E*), and A_2A_R (*ADORA2A*) row *z* score and log_2_ count per million (log_2_ CPM) in CD8^+^ T cells from scRNA-Seq studies. (**D**) Representative flow cytometry plot and CD73 frequency on CD8^+^ T cells from healthy controls (*n* = 42) and untreated patients with STAT3 GOF (*n* = 10). (**E**) Representative histogram of A_2A_R expression on CD8^+^ T cells in healthy controls (*n* = 18) and untreated patients with STAT3 GOF (*n* = 5). (**F**) Schematic of 14-day in vitro culture of healthy control CD8^+^ T cells activated with αCD3/αCD28 with or without IL-21 or IL-27. Time course of (**G**) CD39, (**H**) CD73, and (**I**) A_2A_R induction over 14 days followed by frequency (*n* = 10) and MFI quantification (*n* = 4) at day 14. Data are pooled from more than 3 independent experiments for **A**, **D**, and **G**–**I**, and from 2 experiments for **E**. Data represent mean ± SEM. For **A**, **D**, and **E**: *P ≤ 0.05 and **P ≤ 0.01 by Mann-Whitney test. For **G**–**I**: *P ≤ 0.05, **P ≤ 0.01, ***P ≤ 0.001, ****P ≤ 0.0001 by repeated-measures 1-way ANOVA with Tukey’s multiple-comparison test. Figure 3B (https://BioRender.com/otmaeli) and Figure 3F (https://BioRender.com/lmy3557) were created in BioRender.

We investigated whether immune regulatory pathways might be impaired and thus contribute to dysregulated CD8^+^ T cell function. Although there are multiple regulatory systems and networks in the immune system that could be engaged to counteract chronic inflammation in STAT3 GOF, we noted that compared with healthy controls, patient CD8^+^ T cells displayed elevated levels of an ectoenzyme, CD39, that hydrolyzes extracellular ATP into ADP and AMP (Figure 3A and Supplemental Figure 5A). Expression of CD39 was also increased on immune cells, including CD4^+^ T cells, Tregs, and NK cells (Supplemental Figure 5, B–D). In cells constitutively expressing CD39, including B cells, monocytes, and DCs, expression was comparable between STAT3 GOF and healthy controls (Supplemental Figure 5, E–G). Downstream of CD39, CD73 hydrolyzes AMP into the immunosuppressive molecule adenosine, and A_2A_R is a key adenosine receptor on T cells (Figure 3B). The purinergic axis has been shown to promote forms of CD8^+^ T cell dysfunction including T cell exhaustion (T_EX_), a state of impaired function that occurs in the setting of chronic exposure to antigen and inflammation, which is characterized by increased expression of inhibitory receptors and altered transcriptional networks and epigenetic poise (3, 40, 43, 54, 55). In this context, CD39 has been used as a marker to identify terminally differentiated exhausted CD8^+^ T cells (39). To evaluate whether T_EX_ is present in STAT3 GOF, we assessed expression of TOX, a key transcriptional and epigenetic driver of T_EX_, in patients with STAT3 GOF, using spectral flow cytometry. We recapitulated the differences we previously identified using CyTOF in CD8^+^ T cell differentiation (Figure 1 and Supplemental Figure 6A) and found no significant difference in TOX protein levels in non-naive CD8^+^ T cells from patients with STAT3 GOF and healthy controls (Supplemental Figure 6B). When we evaluated the expression of a gene set with increased expression in T_EX_ (56) in our scRNA-Seq data, we did not find a significant enrichment in non-naive CD8^+^ T cells from patients with STAT3 GOF (Supplemental Figure 6C). Overall, these data are not consistent with T_EX_ playing an important role in STAT3 GOF. With regard to other forms of CD8^+^ T cell dysregulation, a previous study identified increased frequencies of CD57^+^ effector CD8^+^ T cells in patients with STAT3 GOF; these cells shared similarities with effector NKG2D^+^CD8^+^ T cells that contribute to autoimmune and inflammatory pathology in a mouse model of STAT3 GOF (30). In our cohort, we did not identify increased expression of NKG2D on human CD8^+^ T cells (Supplemental Figure 6D) (30). However, we found an increased frequency of CD57^+^CD8^+^ T cells but minimal coexpression of CD57 with CD39, suggesting that both CD57^+^ and CD39^+^ populations are present as nonoverlapping CD8^+^ T cell states in STAT3 GOF (Supplemental Figure 6E). Having identified this increase in CD39^+^CD8^+^ T cells in STAT3 GOF, we sought to further define the purinergic axis in STAT3 GOF.

Since CD39 acts in conjunction with CD73 and A_2A_R, we next evaluated whether downstream components of this regulatory pathway were altered in expression. scRNA-Seq analysis revealed that expression of CD73 (*NT5E*) and A_2A_R (*ADORA2A*) were both decreased on STAT3 GOF total CD8^+^ T cells compared with healthy controls at the transcript level (Figure 3C). We evaluated protein levels by flow cytometry of CD73 and A_2A_R and confirmed that CD73 and A_2A_R levels were decreased on CD8^+^ T cells from patients with STAT3 GOF (Figure 3, D and E). Within other immune cells, CD73 expression was only increased in NK cells in STAT3 GOF (0.27% vs. 0.77%) (Supplemental Figure 7A). Analysis of CD39 and CD73 coexpression revealed that B cells coexpressed CD39 and CD73, but coexpression frequencies did not differ between STAT3 GOF and healthy controls; however, monocytes expressed an increased frequency of CD39^+^CD73^+^ cells in patients with STAT3 GOF versus healthy controls (15.88% vs. 6.45%) (Supplemental Figure 7B). Meanwhile, A_2A_R levels were only decreased within CD3^+^ T cells (Supplemental Figure 7C). We next evaluated expression across CD8^+^ T cell differentiation states. First, we showed the frequency of CD39^+^ (Supplemental Figure 7D), CD73^+^ (Supplemental Figure 7E), and A_2A_R^+^ (Supplemental Figure 7F) cells within naive (T_N_) or non-naive (T_NN_) CD8^+^ T cells, and then within non-naive subsets (T_CM_ vs. T_EM_ vs. T_EMRA_). The proportion of naive and T_CM_ CD8^+^ T cells that expressed CD39 was increased (Supplemental Figure 7D). CD73 and A_2A_R were decreased in proportion within naive and non-naive CD8^+^ T cells, and within T_CM_, there was a significant decrease in the proportion of CD73^+^ cells and a trending decrease in the proportion of CD73^+^ T_EM_ (Supplemental Figure 7E). The proportion of A_2A_R^+^ cells was decreased across all non-naive CD8^+^ T cell subsets (Supplemental Figure 7F). Moreover, there was a trending decrease in CD73 expression in T_N_ and a significant decrease in the amount of A_2A_R in T_NN_ in STAT3 GOF (Supplemental Figure 7, E and F). When we evaluated across multiple experiments, we saw an increase in CD39 MFI in T_N_ (Supplemental Figure 7D) and a trend toward decreased CD73 MFI in patients with STAT3 GOF (Supplemental Figure 7E). Thus, STAT3 GOF affects the proportion of CD8^+^ T cells expressing CD39, CD73, and A_2A_R and the expression of each protein.

### STAT3 signaling regulates expression of CD39 on CD8^+^ T cells from healthy controls.

Having identified dysregulation of CD39, CD73, and A_2A_R expression, we sought to better understand the contribution of STAT signaling in regulating purinergic pathway member expression. There is evidence that the CD39 (*Entpd1*) locus is directly bound by STAT3 in mouse CD4^+^ T cells (57). We also found evidence that CD39 (*Entpd1*), CD73 (*Nt5e*), and A_2A_R (*Adora2a*) may be directly bound by IL-27–driven STAT3 in mouse CD4^+^ T cells, based on reanalysis of published ChIP-Seq data (Supplemental Figure 8A) (58–60). These findings are consistent with work demonstrating that IL-6–mediated STAT3 activation promotes CD39 expression in CD4^+^ Th17 cells (57) and is consistent with increased CD39 expression in CD4^+^ T cells (Supplemental Figure 5B), including Tregs (Supplemental Figure 5C) in patients with STAT3 GOF. Prior data and our work thus support that *Entpd1* is a direct target of STAT3 in mouse CD4^+^ T cells. Although CD8^+^ T cells from patients with STAT3 GOF have increased expression of CD39 (Figure 3A and Supplemental Figure 5A), the impact of amplified STAT3 signaling on CD39 expression in human CD8^+^ T cells remained to be determined.

Since T cell activation is governed by a variety of signals, including TCR engagement and cytokine signals, we first assessed the kinetics of CD39 upregulation upon αCD3/αCD28 activation in healthy control PBMCs (61, 62). At baseline, CD8^+^ T cells expressed low levels of CD39, but CD39 expression was upregulated over 96 hours (Supplemental Figure 9A). We next tested the cell-intrinsic impacts of amplifying STAT3 signaling on CD39. STAT3 is activated downstream of a variety of cytokines, including IL-6, which we and others have identified as being increased systemically in STAT3 GOF (Figure 2F) (63). To test whether increasing STAT3 signaling induces higher levels of CD39, we activated purified healthy control CD8^+^ T cells with αCD3/αCD28 plus cytokines that induce phospho-STAT3 at the tyrosine 705 residue (pSTAT3^Y705^) (Supplemental Figure 9B). Cytokines including IL-6, IL-10, IL-21, and IL-27 also phosphorylate STAT1 at tyrosine 701 (pSTAT1^Y701^) to varying degrees (Supplemental Figure 9, B and C). Among these cytokines, IL-21 preferentially induced pSTAT3 over pSTAT1, giving the largest pSTAT3/pSTAT1 ratio, while IL-27 preferentially induced pSTAT1 over pSTAT3 (Supplemental Figure 9C). This allowed us to estimate how the relative magnitude of STAT1 and STAT3 signaling influences purinergic molecule expression. CD39 was robustly induced by αCD3/αCD28 activation and augmented by IL-6, IL-10, IL-21, and IL-27 but not IL-2; cytokine treatment alone was not sufficient to induce CD39 expression (Supplemental Figure 9, D and E). We next assessed pSTAT3 levels within CD8^+^ T cells that did not upregulate CD39 (CD8^+^CD39^lo^) versus those that did (CD8^+^CD39^hi^). Irrespective of activation conditions (i.e., αCD3/αCD28 with or without cytokine), pSTAT3 levels were higher in CD8^+^CD39^hi^ cells relative to CD8^+^CD39^lo^ cells (Supplemental Figure 9F). Having assessed differences in pSTAT3 levels in CD39 expressing and nonexpressing cells, we tested whether this increase in CD39 expression is dependent on STAT3 signaling. To do this, we blocked STAT3 phosphorylation with the STAT3 inhibitor STATTIC (64). This drug primarily inhibits STAT3, though at high doses it can also impair STAT1 activity and cell viability (Supplemental Figure 10, A and B). Therefore, we identified a STATTIC dose (0.5 μM) that did not affect cell viability but reduced pSTAT3 with minimal impact on pSTAT1 (Supplemental Figure 10, C and D). In αCD3/αCD28-activated culture conditions with or without STAT3-activating cytokines, incubation with STATTIC decreased pSTAT3 levels (Supplemental Figure 10E) and the frequency of CD39^+^CD8^+^ T cells (Supplemental Figure 10F). This suggests that STAT3 contributes to the regulation of CD39 expression in a TCR-dependent manner in healthy control CD8^+^ T cells.

### Amplitude of STAT3 signaling regulates CD39 expression in CD8^+^ T cells.

Having established that STAT3 can regulate CD39 expression in vitro, we further evaluated the connection between the amplitude of STAT3 signaling and CD39 expression. We therefore compared patients with STAT3 GOF to a cohort of patients with STAT3 dominant-negative (DN) hyper IgE syndrome (Supplemental Table 1). STAT3 DN (also known as Job syndrome or autosomal dominant hyper IgE syndrome), is a monogenic inborn error of immunity caused by heterozygous STAT3 variants, which yield decreased but not absent STAT3 signaling (65–68). We found that, at baseline, CD39 expression in CD8^+^ T cells from patients with STAT3 DN was comparable to healthy controls and reduced when compared with STAT3 GOF patients (Supplemental Figure 11A). However, upon activation, CD8^+^ T cells from patients with STAT3 DN had reduced CD39 induction compared with healthy controls and patients with STAT3 GOF (Supplemental Figure 11B). By comparing the impact of amplifying and diminishing STAT3 signaling in STAT3 GOF and STAT3 DN, respectively, we found evidence to support the hypothesis that modulating the amplitude of STAT3 signaling regulates CD39 expression ex vivo upon activation of CD8^+^ T cells.

To test whether STAT3 GOF variants intrinsically induce higher levels of CD39, we used a system in which STAT3 signaling was endogenously absent in mouse T cells to directly compare the impact of STAT3 GOF variants on CD39 levels (Supplemental Figure 12A). *Stat3^fl/fl^* mice were crossed to CD4-Cre mice to yield *Stat3*^fl/fll^ CD4-Cre, which specifically ablates STAT3 expression in all CD3^+^ T cells (69). To assess the ability of STAT3 GOF variants to mobilize target genes, we transduced T cells from WT (*Stat3^fl/fl^*) mice or mice with STAT3-deficient T cells (*Stat3^fl/fl^* CD4-Cre) with either WT or STAT3 GOF patient variants and measured CD39 by flow cytometry (Supplemental Figure 12B). Importantly, transduced T cells from WT *Stat3^fl/fl^* mice approximated the scenario in humans where WT and mutant alleles coexist. In control *Stat3*^fl/fll^ mice containing endogenous WT STAT3, introduction of the p.Q344H variant was sufficient to drive higher levels of CD39 compared with introducing additional WT STAT3 (Supplemental Figure 12C). In STAT3-deficient T cells, CD39 expression was low, and introduction of WT STAT3 induced expression of CD39; the STAT3 GOF variant (p.Q344H) drove even higher levels of CD39 than WT STAT3 (Supplemental Figure 12C). Together, this suggests that activating variants that increase STAT3 signaling are sufficient to augment CD39 expression upon TCR engagement.

### STAT1 and STAT3 regulate expression of CD73 and A_2A_R on CD8^+^ T cells from healthy controls.

Having observed decreased frequencies of CD73^+^CD8^+^ and A_2A_R^+^CD8^+^ T cells in patients with STAT3 GOF, we next wanted to test whether STAT3 positively or negatively regulated expression of these molecules. Using our in vitro model of healthy control CD8^+^ T cell activation with αCD3/αCD28 with or without cytokines, we found that 96 hours of activation with αCD3/αCD28 reduced CD73 levels on healthy control CD8^+^ T cells (Supplemental Figure 13, A and B). However, the frequency of CD73^+^ cells trended toward increased only in conditions with αCD3/αCD28 plus IL-21 relative to αCD3/αCD28 alone (Supplemental Figure 13B). Recent work in mice has shown that CD73 is downregulated upon initial activation but is reexpressed during the resolution phase of an immune response after infection (70). Therefore, we tested whether more prolonged exposure of CD8^+^ T cells to STAT3-activating cytokines in the setting of αCD3/αCD28 activation would increase CD73 and A_2A_R. Here, we selected IL-21 and IL-27 because both can induce pSTAT3 to varying magnitudes, with IL-21 preferentially activating pSTAT3 over pSTAT1 and IL-27 preferentially activating pSTAT1 over pSTAT3 (Supplemental Figure 9C). We activated healthy control CD8^+^ T cells with αCD3/αCD28 with or without IL-21 or IL-27 for 4 days followed by fresh media with or without cytokine exchange every 4–6 days for a total of 14 days (Figure 3F). Here, we found that prolonged coculture with IL-21 resulted in increased frequencies and expression of CD39^+^CD8^+^ and CD73^+^CD8^+^ T cells compared with coculture without cytokine or with IL-27 (Figure 3, G and H, and Supplemental Figure 13C). Moreover, IL-21 led to increased frequencies of CD8^+^ T cells coexpressing CD39 and CD73 compared with the αCD3/αCD28 with or without IL-27 conditions (Supplemental Figure 13D). Lastly, IL-21 promoted a trend toward reexpression of A_2A_R at day 14 relative to IL-27 (Figure 3I and Supplemental Figure 13E). Furthermore, CD8^+^ T cells at day 10 of in vitro culture with αCD3/αCD28 with or without IL-21 or IL-27 showed that relative to IL-21, IL-27 led to a trending decrease in CD73 (*P* = 0.057) and A_2A_R positivity (*P* = 0.08; Supplemental Figure 13, F and G). Moreover, on day 10, IL-27 led to higher levels of pSTAT1 and lower levels of CD73, while IL-21 led to higher levels of both pSTAT3 and CD73 (Supplemental Figure 13F). We observed similar trends with A_2A_R (Supplemental Figure 13G). Overall, although both IL-21 and IL-27 can induce STAT3 signaling, an inflammatory milieu that also promotes a strong pSTAT1 signal (e.g., IL-27) inhibits re-upregulation of CD73 and may inhibit A_2A_R as well. Increased STAT1 signaling in the context of increased STAT3 signaling would be consistent with the upregulated IFN signatures detected in our scRNA-Seq analysis and systemic inflammation, as indicated by increased levels of IFN-γ and CXCL9, and could contribute to the purinergic molecule expression patterns in STAT3 GOF.

### Patients with STAT3 GOF receiving disease-modifying therapy have partial restoration of dysregulated CD8^+^ T cell function and purinergic molecule expression.

**Figure 4:**
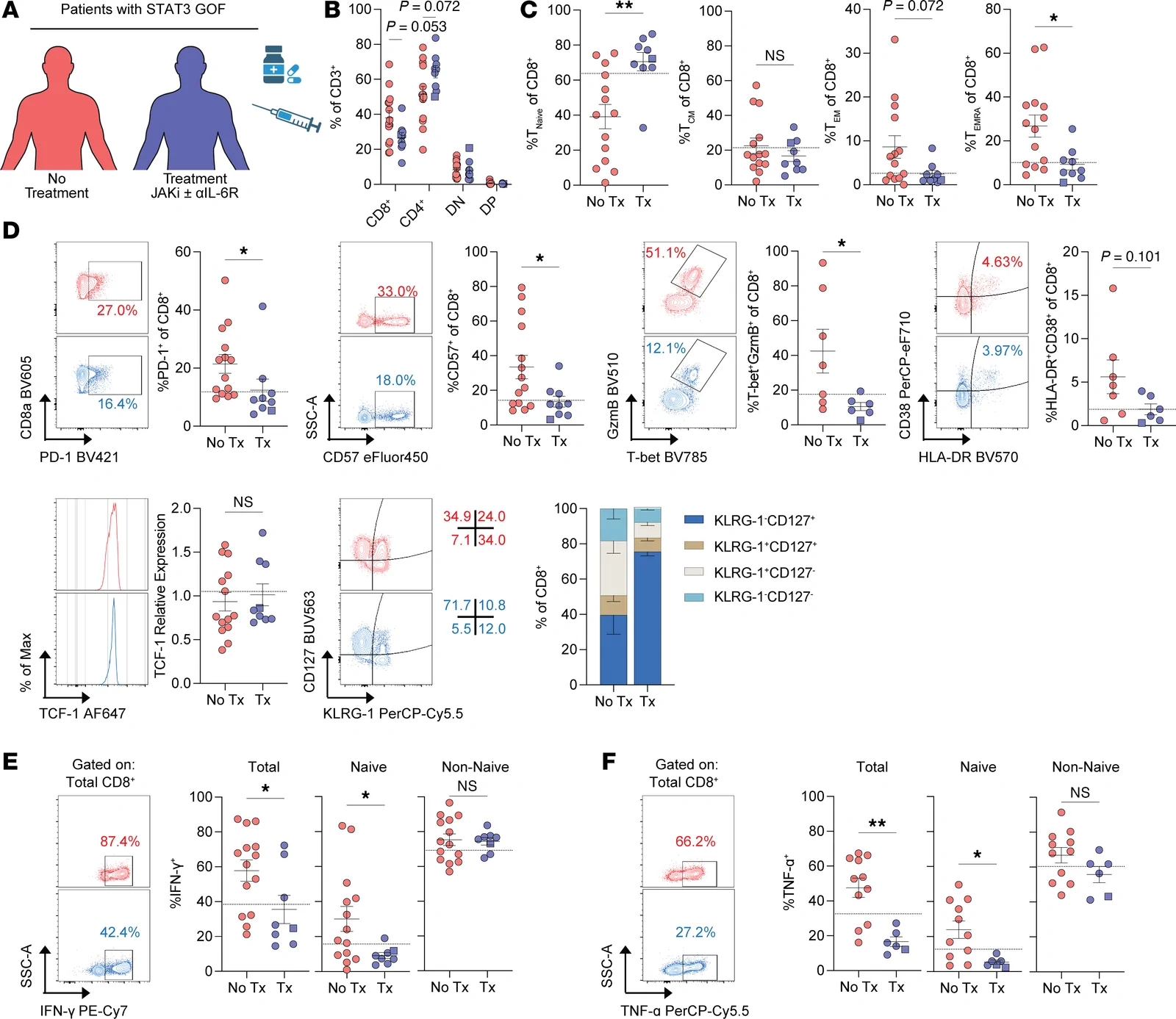
Impact of therapy (JAKi with or without αIL-6R) on aberrant CD8^+^ T cell phenotype and function. (**A**) Study schematic comparing samples from patients with STAT3 GOF not receiving immune modulatory therapy (red) to samples from patients with STAT3 GOF receiving immune modulatory therapy (blue). (**B**) Proportion of CD8^+^, CD4^+^, double-negative (DN), and double-positive (DP) cells within CD3^+^ cells in untreated (*n* = 14) and treated (*n* = 9) patients with STAT3 GOF. Impact of treatment on (**C**) CD8^+^ T cell differentiation and (**D**) activation markers in untreated (*n* = 7–14) and treated (*n* = 6–9) patients with STAT3 GOF. Impact of treatment on (**E**) IFN-γ cytokine production (*n* = 14 untreated STAT3 GOF patients; *n* = 8 treated STAT3 GOF patients) and (**F**) TNF-α cytokine production by CD8^+^ T cell subsets (*n* = 11 untreated STAT3 GOF patients; *n* = 6 treated STAT3 GOF patients). IFN-γ and TNF-α values from untreated STAT3 GOF patients used for Figure 5, E and F, were used in Figure 2D and Figure 2C, respectively. Blue square represents patient treated with rapamycin, rather than JAKi (blue circles). Data are pooled from 3 or more independent experiments for **B** and **C** and from 2–3 experiments for **D**–**F**. Data represent mean ± SEM. *P ≤ 0.05, **P ≤ 0.01 by Mann-Whitney test. Solid dashed line represents the average of 30-plus healthy controls. Figure 4A was created in BioRender (https://BioRender.com/4ut4e0o).

Patients with severe STAT3 GOF are often treated with immunomodulatory therapy (e.g., JAKi with or without α-IL-6R, tocilizumab) that inhibit the JAK/STAT signaling pathway upstream of STAT proteins (31). We evaluated the impact of in vivo modulation of STAT signaling with samples from patients with STAT3 GOF receiving JAKi with or without α-IL6R (Figure 4A) (16, 17, 31). Treated patients with STAT3 GOF demonstrated a trending decrease in the frequency of CD8^+^ T cells and a significant increase and decrease, respectively, in the proportion of naive and T_EMRA_ CD8^+^ T cells compared with untreated patients (Figure 4, B and C). Relative to untreated patients, treated patients demonstrated a decrease in markers indicative of CD8^+^ T cell activation including PD-1, CD57, and Granzyme B and T-bet coexpressing cells (Figure 4D). Although we did not detect any changes in TCF-1 expression, treatment led to decreases in KLRG-1 and increases in CD127 expression (Figure 4D). Moreover, treatment resulted in normalization of differentiation and phenotypic differences (e.g., activation markers and inhibitory receptor expression) when compared with healthy controls (Supplemental Figure 14, A and B). We have demonstrated that CD8^+^ T cells, particularly naive CD8^+^ T cells, from patients with STAT3 GOF had greater capacity to produce inflammatory cytokines relative to healthy controls (Figure 2, C and D). Compared with untreated patients with STAT3 GOF, treatment led to a significant decrease in IFN-γ and TNF-α production capacity, particularly within total and naive CD8^+^ T cells, such that cytokine production levels in treated patients were comparable to healthy controls (Figure 4, E and F, and Supplemental Figure 14C). Thus, patients with STAT3 GOF receiving immunomodulatory therapy have restoration of these aspects of dysregulated CD8^+^ T cell phenotype and function.

**Figure 5:**
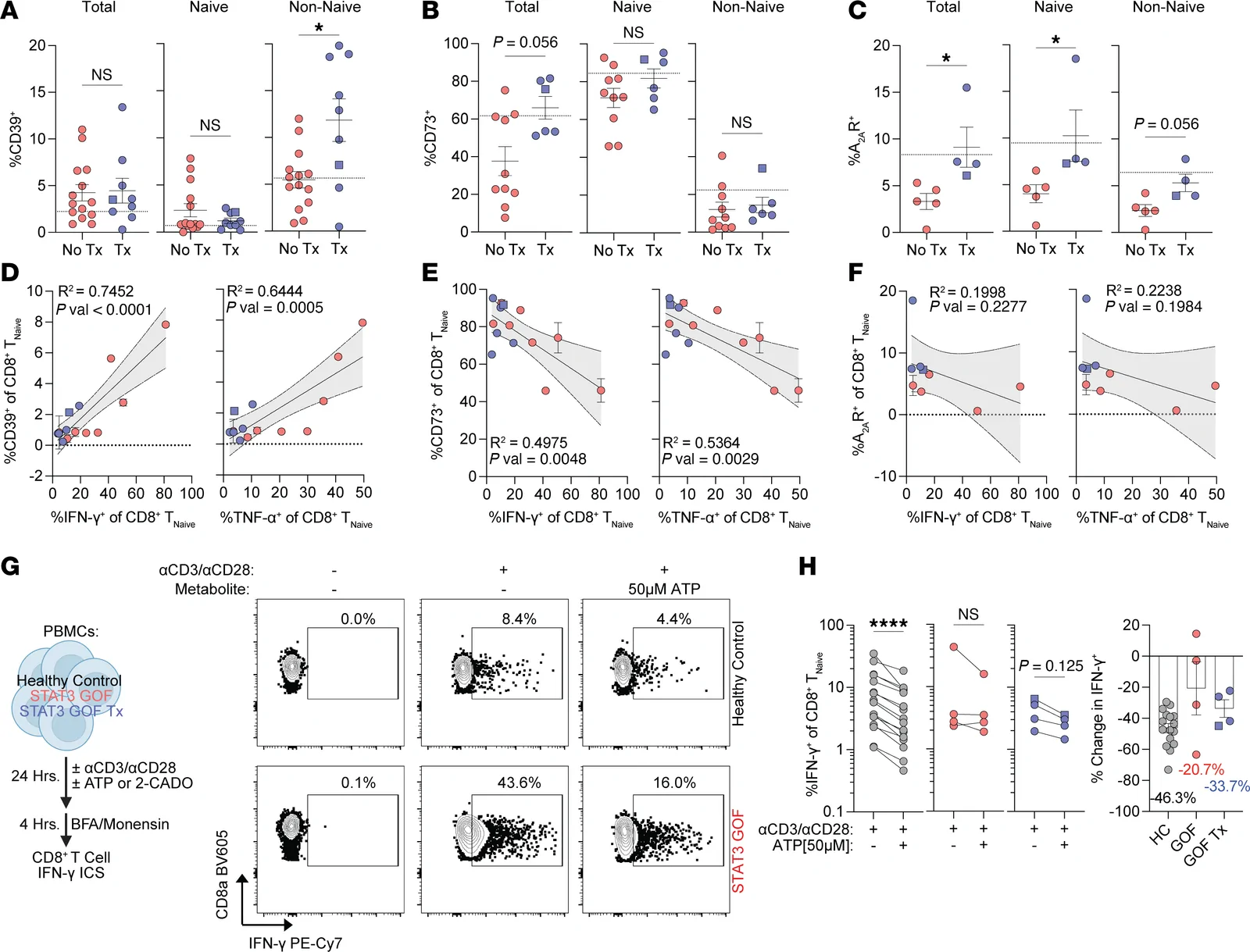
Altered expression of purinergic signaling molecules correlates with aberrant function and susceptibility to inhibition and is partially corrected with therapy. (**A**) CD39 expression on CD8^+^ T cells in untreated (*n* = 14) and treated (*n* = 9) patients with STAT3 GOF. (**B**) CD73 expression on CD8^+^ T cells in untreated (*n* = 10) and treated (*n* = 6) patients with STAT3 GOF. (**C**) A_2A_R expression on CD8^+^ T cells in untreated (*n* = 5) and treated (*n* = 4) patients with STAT3 GOF. Correlation between IFN-γ (left) and TNF-α (right) positivity by (**D**) CD39, (**E**) CD73, and (**F**) A_2A_R positivity in naive CD8^+^ T cells. (**G**) Experimental schematic of αCD3/αCD28 activation with or without ATP or 2-CADO for 24 hours and representative plots of naive CD8^+^IFN-γ^+^ T cells. (**H**) Paired analysis and quantification of percentage change in naive CD8^+^IFN-γ^+^ T cells in αCD3/αCD28 plus ATP conditions relative to αCD3/αCD28. A total of *n* = 16 healthy controls (gray), *n* = 4 untreated (red), and *n* = 4 treated (blue) patients with STAT3 GOF were analyzed. For **A**–**C**: values in total CD8^+^ T cells from untreated patients with STAT3 GOF were also used in Figure 3A (CD39), Figure 3D (CD73), and Figure 3E (A_2A_R). For **A**–**H**: blue square represents patient treated with rapamycin, rather than JAKi. Data are pooled from 3 or more and 2–3 independent experiments for **A** and **B**–**F,** respectively. For **H**: data are pooled from 2 independent experiments with 3 repeat healthy controls and 1 repeat patient with STAT3 GOF; repeated samples were averaged into 1 data point per participant. Data represent mean ± SEM. *P ≤ 0.05, ****P ≤ 0.0001 by Mann-Whitney test, Wilcoxon’s matched-pairs signed rank test, Kruskal-Wallis test with Dunn’s multiple comparisons test, or simple linear regression (with the shaded area indicating 95% CI), as appropriate.

Given that dysregulation of purinergic pathway members was most evident in CD8^+^ T cells, we next explored the impact of therapy on this pathway. We found that relative to untreated patients, patients receiving therapy did not demonstrate decreases in CD39^+^ cells (Figure 5A). In fact, relative to healthy controls, treated patients with STAT3 GOF still demonstrated a trending increase in CD39 positivity (*P* = 0.057) (Supplemental Figure 14D). Relative to untreated patients, treated patients demonstrated a trending increase in CD73^+^ cells (*P* = 0.056) (Figure 5B), but relative to healthy controls, there was no difference in CD73^+^ cells in treated patients (Supplemental Figure 14D). Additionally, JAKi increased the frequency of A_2A_R^+^ cells relative to untreated patients (Figure 5C), and there was no difference in the frequency of A_2A_R^+^ cells between treated patients and healthy controls (Supplemental Figure 14D). Last, patients who are clinically started on JAKi may be more affected by STAT3 GOF at baseline, hence the initiation of therapy. For 2 patients, paired pre-JAKi– and post-JAKi–treated samples were available for analysis: here, CD39 expression was decreased at the time point with JAKi compared with pretreatment (Supplemental Figure 14E).

At baseline, patients with STAT3 GOF demonstrated increased potential for naive CD8^+^ T cells to secrete inflammatory cytokines (Figure 2, C and D) along with reduced levels of the terminal components of the immunoregulatory purinergic signaling cascade (Figure 3, D and E), which were found to be partially corrected with JAKi with or without α-IL6R (Figure 5, A–C). To test for an association between the observed decrease in the terminal components of the purinergic signaling cascade and altered function within naive CD8^+^ T cells in STAT3 GOF, we correlated CD8^+^ T cell cytokine production levels with expression of CD39, CD73, and A_2A_R among patients with STAT3 GOF for whom we had paired purinergic molecule phenotypic and functional data. We found that the frequency of IFN-γ^+^ and TNF-α^+^ naive CD8^+^ T cells positively correlated with the frequency of CD39^+^ naive CD8^+^ T cells, and the frequency of IFN-γ^+^ and TNF-α^+^ naive CD8^+^ T cells was negatively associated with CD73^+^ cells (Figure 5, D and E). Meanwhile, increased frequencies of IFN-γ^+^ and TNF-α^+^ cells were generally associated with decreases in the frequency of A_2A_R^+^ cells, though not statistically significant (Figure 5F). As the frequency of IFN-γ^+^ cells increased, this correlated with decreases in the frequency of CD73^+^ and A_2A_R^+^ cells within total and non-naive CD8^+^ T cells (Supplemental Figure 15, A and B); TNF-α^+^ cells also demonstrated similar trends (Supplemental Figure 15, C and D). Overall, downregulation of terminal components of the purinergic signaling pathway are associated with increased proinflammatory cytokine production from CD8^+^ T cell subsets.

Given decreased CD73 and A_2A_R levels on CD8^+^ T cells in STAT3 GOF, we hypothesized that the immunoregulatory role of the purinergic pathway may be dysfunctional in STAT3 GOF. We tested the impact of engaging this pathway in PBMCs from healthy controls, untreated patients with STAT3 GOF, and patients with STAT3 GOF receiving JAKi with or without α-IL6R by activating PBMCs with αCD3/αCD28 for 24 hours with or without ATP (which can be metabolized to adenosine) or 2-Chloroadenosine (2-CADO; agonist for A_2A_R) and stained for intracellular IFN-γ (Figure 5G). We did not detect differences in cell viability between groups in the assay (Supplemental Figure 16A). We had previously observed effector function dysregulation of naive CD8^+^ T cells from patients with STAT3 GOF, so we focused our analyses of the impact of purinergic signaling on this CD8^+^ T cell subset. Relative to αCD3/αCD28 alone, healthy controls demonstrated a significantly reduced frequency of IFN-γ^+^ naive CD8^+^ T cells when activated with αCD3/αCD28 plus ATP (Figure 5H). In contrast, this was not seen in naive CD8^+^ T cells from untreated patients with STAT3 GOF (Figure 5H). Naive CD8^+^ T cells from untreated patients with STAT3 GOF demonstrated a smaller reduction in IFN-γ positivity (change = –20.7%) relative to healthy controls (change = –46.3%) in the presence of exogenous ATP. Meanwhile, naive CD8^+^ T cells from patients receiving JAKi demonstrated a slight increase in inhibition relative to untreated patients with STAT3 GOF (change = –33.7%) (Figure 5H). Similar effects were seen on IFN-γ MFI within naive cells (Supplemental Figure 16B). Relative to αCD3/αCD28 conditions, only healthy controls activated with αCD3/αCD28 plus 2-CADO demonstrated a statistically significant inhibition of IFN-γ frequency and MFI in naive CD8^+^ T cells (Supplemental Figure 16, C and D). However, the overall change in IFN-γ frequency and MFI in the presence of 2-CADO was comparable between healthy controls and both untreated and treated patients with STAT3 GOF (Supplemental Figure 16, C and D). Altogether, these data suggest that the purinergic pathway is dysfunctional in patients with STAT3 GOF and that IFN-γ production by naive CD8^+^ T cells from patients with STAT3 GOF appears to be less susceptible to ATP-derived adenosine inhibition than healthy controls. Lastly, the purinergic axis and secretion of inflammatory cytokines by CD8^+^ T cells are partially normalized by JAKi treatment of patients with STAT3 GOF.

## Discussion

Here, we have analyzed the CD8^+^ T cell compartment in a large cohort of patients with STAT3 GOF at a phenotypic, functional, and transcriptional level. We have identified increased capacity for inflammatory cytokine production and dysregulated purinergic signaling by human naive CD8^+^ T cells from patients with STAT3 GOF. We identified changes in the purinergic signaling axis (with increased CD39 expression and decreased CD73 and A_2A_R expression on CD8^+^ T cells) and evidence that these changes contribute to the dysregulated inhibitory function of this pathway. Changes in expression of these proteins on CD8^+^ T cells were found by flow cytometry in naive T cells (which were decreased in proportion) and non-naive T cells (which were increased in proportion); within non-naive cells, changes in expression patterns were driven by T_CM_ (which were unchanged in proportion), so we attribute altered pathway function to changes in protein expression, and not simply the change in relative proportions of differentiation states. Systemically, in plasma, patients with STAT3 GOF have elevated levels of STAT3-activating cytokines (e.g., IL-6 and IL-10) and IFN-γ (and downstream target CXCL9), and scRNA-Seq revealed evidence of amplified IFN-α/IFN-γ gene sets in CD8^+^ T cells. CD8^+^ T cells from patients with STAT3 GOF demonstrate impaired purinergic-mediated regulation of inflammatory cytokine production. Given that a targeted, although off-label, therapy exists for this disease (31), we were able to assess the impact of in vivo blockade of JAK/STAT signaling by evaluating CD8^+^ T cells from patients with STAT3 GOF receiving JAKi. This led to partial normalization of purinergic axis components and inflammatory cytokine production by CD8^+^ T cells. The persistence of some aspects of the phenotypic and functional dysregulation of CD8^+^ T cells in patients with STAT3 GOF receiving JAK inhibition therapy may imply a role for epigenetic changes in CD8^+^ T cells, an important area for future work. Overall, our data suggest that in STAT3 GOF, dysregulation of the immunoregulatory purinergic signaling axis contributes to the presence of hyperactivated and proinflammatory naive CD8^+^ T cells and thus plays a role in the pathogenesis of autoimmunity in these patients.

We sought to define the role of STAT signaling in regulating purinergic molecule expression. The interplay between STAT3 and purinergic signaling is deepened in our work and links to prior work, with clear evidence that CD39 is a STAT3 target gene, as well as some evidence that CD73 is also a target of STAT3 (57). Future work will fully define whether CD73 and/or A_2A_R are directly or indirectly affected by STAT3 signaling. However, there may also be important impacts of STAT1 signaling on CD73 to define in this disease, with prior data demonstrating that IFN-γ promoted expression of TRIM21, a ubiquitin ligase, which downregulated CD73 (71). In an in vitro model system, we showed that prolonged culture of CD8^+^ T cells with αCD3/αCD28 with IL-27 led to increased STAT1 signaling and decreased CD73 and A_2A_R reexpression, consistent with the patterns in CD8^+^ T cells from patients with STAT3 GOF. In a small group of patients with STAT3 GOF, we found that CD8^+^ T cells had less inhibition of IFN-γ production with inclusion of ATP or adenosine, supporting the idea that changes in purinergic molecules affect CD8^+^ T cell function. Further studies are needed to fully define the cell-intrinsic and cell-extrinsic impact of modifying purinergic signaling on CD8^+^ T cell–mediated autoimmunity in STAT3 GOF.

When attempting to dissect molecular mechanisms by which STAT3 GOF leads to this impact on purinergic signaling, it is important to note that cytokines can simultaneously activate multiple STAT proteins. Activation not only leads to homodimer formation, but also heterodimer formation, and the balance of dimers can be quite different between cytokines that activate a given STAT protein. For example, in CD4^+^ T cells, IL-6 and IL-27 have been demonstrated to induce not only STAT1:STAT1 and STAT3:STAT3 homodimers, but also STAT1:STAT3 heterodimers (59, 72, 73). As we have shown, IL-27 is more likely to induce pSTAT1 than pSTAT3, and IL-21 is more likely to induce pSTAT3 than pSTAT1, though both cytokines activate both STAT proteins. In addition, it has previously been shown in mice that STAT3 can greatly impact how STAT1 interacts with DNA (59). With regard to primary cells, our scRNA-Seq and plasma proteomic profiling from patients with STAT3 GOF revealed evidence of a strong type I/II IFN signature. The relative balance of these 2 transcription factors, in addition to any cross-regulation, and interactions between STAT1 and STAT3 proteins may alter aspects of signaling in the more complex in vivo environment, which would consequently affect the type or magnitude of an immune response and may be a key determinant of both clinical phenotype and disease severity. Specifically, whether there is altered hetero- or homo-dimerization of STAT3 and STAT1, if this affects the function of STAT1 and/or STAT3, respectively, and whether this could have an impact on the IFN signature and inflammatory milieu observed in patients with STAT3 GOF, are important areas for future research and will require application of novel assays, including those that permit quantification of heterodimerization versus homodimerization (59, 74).

There are limitations to our studies. First, the rarity of STAT3 GOF limits access to blood samples from patients, although here we have assembled and analyzed a large cohort of patients with STAT3 GOF, permitting a thorough evaluation of CD8^+^ T cells at a phenotypic, functional, and transcriptional level. Furthermore, obtaining samples from patients with STAT3 GOF prior to treatment with immune modulatory therapy is particularly challenging, given that patients are typically started promptly on various immune modulatory therapies after presentation due to the severity of illness. Second, although we focused on the CD8^+^ T cell compartment in STAT3 GOF syndrome, most of our analyses in experiments that include primary patient samples were performed using PBMCs. This is again due to the challenges in obtaining sufficient blood samples to be able to purify enough STAT3 GOF CD8^+^ T cells for assays, especially since many of these patients are children and are often lymphopenic. Third, we could only make correlations between cytokine production and expression of purinergic pathway members for individuals for whom we have paired CD39/CD73/A_2A_R and cytokine data, which is a subset of our cohort. Finally, in our in vitro models, the duration of exposure to amplified STAT3 signals in healthy control CD8^+^ T cells is limited compared with cells from patients with STAT3 GOF, whose cells experience lifelong alterations in STAT3 signaling through differentiation and activation and may not fully recapitulate the full breadth of signals that cells receive in vivo.

Overall, this study identifies and implicates impaired purinergic signaling in STAT3 GOF as a mechanism of dysregulated CD8^+^ T cell phenotype and function, likely contributing to autoimmunity. This contribution to our conception of how purinergic signaling affects CD8^+^ T cell functionality may also have broader implications for other primary immune regulatory disorders, other causes of autoimmunity, and in more common causes of chronic inflammation. In conclusion, this study improves our understanding of STAT3 GOF pathogenesis by defining a mechanism of CD8^+^ T cell dysregulation due to altered purinergic signaling, provides cellular and functional biomarkers for monitoring disease, and elucidates how targeted therapy in the form of JAKi can exert therapeutic impact.

## Methods

### Sex as a biological variable.

Our study examined samples from male and female patients with STAT3 GOF, STAT3 DN, healthy controls, and mice. In these studies, we did not consider sex as a biological variable. Age, sex, STAT3 variant, and medications for STAT3 GOF and DN are shown in Supplemental Table 1, as is age and sex distribution for healthy controls.

### Statistics.

All statistical analyses were performed using GraphPad Prism (version 10.4.1). When healthy control and/or patient participant samples were run in more than 1 batch of an individual assay, all values obtained for that participant across the batches of that assay were averaged into 1 data point for each participant for plotting and analysis. Statistical significance among groups was calculated with Mann-Whitney test (2-tailed), 1-way ANOVA, linear regression, or Wilcoxon’s matched-pairs signed rank test depending on the design of each experiment and distribution of the data. The number of samples analyzed and exact statistical test for each experiment are in the figure legends or in the figures; for analysis of more than 2 groups, adjusted *P* values were used. A *P* value of 0.05 or less was considered significant.

### Study approval.

In accordance with IRB-approved protocols, written consent (and assent, if appropriate) was obtained from participant or guardian before study enrollment, as below. Participants with STAT3 GOF were recruited with IRB approval (Washington University IRB 201107235; ClinicalTrials.gov NCT03394053 and NCT00404560; Children’s Hospital of Philadelphia IRB 18-15920; Children’s National IRB Pro00009689; Children’s Hospital of Philadelphia IRB 15-01226; National Institute of Allergy and Infectious Diseases (NIAID) NIH IRB 05-I-0213; Texas Children’s Hospital IRB 21453 and 30487). Patients with STAT3 DN were recruited under ClinicalTrials.gov NCT00006150. Healthy controls were recruited under Children’s Hospital of Philadelphia IRB 18-15920 and University of Pennsylvania IRB 834263. Samples were shared via material transfer agreements or collaborative research agreements. For mouse models of STAT3 GOF, STAT3^+/G421R^ mice and littermate controls were maintained at Washington University in St. Louis under previously established guidelines and approvals (26). NOD-STAT3^+/K392R^ mice and littermate controls were maintained at the UCSF animal facility (specific pathogen free) under previously established guidelines and approvals (29). Generation of *Stat3^fl/fl^* CD4-Cre(+) and *Stat3^fl/fl^* CD4-Cre(–) littermate controls was performed as described previously (69). Mice were housed and handled in accordance with NIH guidelines, and all experiments were approved by the University of Miami IACUC.

### Data availability.

Data values used to generate each graph are provided in the Supporting Data Values file. Raw and processed data from scRNA-Seq studies have been deposited in NCBI’s Gene Expression Omnibus (GEO GSE311536).

## Author contributions

JSCD and SEH conceptualized the study; planned the experiments; were responsible for methodology, analysis and visualization; curated the data; and wrote the first draft of the manuscript. JSCD, MSK, SUS, MCD, AAM, PC, ABS, CA Hay, RBL, IN, KY, and CA Howard performed investigations. JSCD, MSK, AVV, WB, CA Hunter, MSA, TPV and SEH contributed to methodology. JSCD, MSK, ABS, AVV, and SEH analyzed data. EDA, SB, CMS, TCP, AT, ISL, AG, SWC, HCS, MDK, OMD, LRFS, SMH, JREB, JWL, NR, AFF, MAC, TPV, and SEH provided resources (patient samples). EGS, MAT, MSA, and MAC provided resources (mouse samples). Funding was acquired by JSCD, RBL, EGS, MAT, HCS, LRFS, NR, MSA, TPV, MAC, and SEH. SEH supervised the overall study. All authors reviewed the manuscript.

## Conflict of interest

The authors have declared that no conflict of interest exists.

## Funding support

This work is the result of NIH funding, in whole or in part, and is subject to the NIH Public Access Policy. Through acceptance of this federal funding, the NIH has been given a right to make the work publicly available in PubMed Central. Approximately 5% of the total project costs are financed with federal funds in the amount of $45,000. The remaining 95% of project costs are financed by nongovernmental sources in the amount of $875,000.

- Howard Hughes Medical Institute Gilliam Fellowship for Advanced Studies GT15736 and Penn Presidential PhD Fellowship (to JSCD).
- NIH National Institute of Child Health and Human Development K12HD047349 and NIH National Institute of General Medical Sciences K23GM159013 (to RBL).
- NIH NIAID P01AI155393, the Jeffrey Modell Diagnostic and Research Center for Primary Immunodeficiencies at St. Louis Children’s Hospital, and the Children’s Discovery Institute of Washington University’s Center for Pediatric Immunology and St. Louis Children’s Hospital (to EGS and MAC).
- NIH NIAID 1K08AI182483 and NIH National Institute of Arthritis and Musculoskeletal and Skin Diseases P30AR073752 (to EGS).
- NIH National Institute of Diabetes and Digestive and Kidney Diseases K12DK133995 (to MAT).
- JDRF Advanced Postdoctoral Fellowship from Breakthrough T1D (to MAT).
- Intramural Research Program of the NIH NIAID with 1ZIAAI001059 (to HCS).
- Jeffrey Modell Foundation and NIH National Center for Advancing Translational Sciences 5UG3TR003908-02 (to LRFS).
- NIH NIAID AI146026 and NIH NIAID AI179680 (to NR).
- NIH NIAID P01AI155393 (to MSA).
- Arthritis National Research Foundation (to TPV).
- NIH NIAID 1K08AI135091, NIH NIAID AI187202, Burroughs Wellcome Fund CAMS, AAAAI Foundation, the Hartwell Foundation, Immune Deficiency Foundation, and Primary Immune Deficiency Treatment Consortium (to SEH).

## Supplementary Material

Supplemental data

Supplemental tables 1-2

Supporting data values

## Version 1 09/08/2026

Electronic publication
