# Supplementary material for: Impaired regulation by purinergic signaling axis contributes to CD8+ T cell dysregulation in STAT3 gain of function
Source: JCI Insight. 2026 Sep 8;11(17):e202959. doi: 10.1172/jci.insight.202959 (PMC13564073; doi:10.1172/jci.insight.202959)
Supplement: Supplementary file 1 [file jciinsight-11-202959-s284.pdf]

Supplemental Material

Impaired regulation by purinergic signaling axis contributes to CD8$^+$ T cell dysregulation in STAT3 GOF

Authors: Jose S. Campos Duran[1,2,3], Montana S. Knight[1,4], Samir U. Sayed[1], Megan C. Dalalo[1], Andrea A. Mauracher[1], Peyton E. Conrey[1], Aaron B. Schultz[5,6], Ceire A. Hay[1,2,3], Robert B. Lindell[2,3,7], Ilona Neale[1,2,3], Kyle Yeakle[1,3], Eric D. Abrams[1], Erica G. Schmitt[8,9], Martin A. Thelin[10,11], Christian A. Howard[2,3], Sara Bluestein[12], Christine M. Seroogy[13], Tamara C. Pozos[14], Akaluck Thatayatikom[15], Ingrid Lundgren[16], Amelie Gauthier[17], Scott W. Canna[2,3,18], Helen C. Su[19], Michael D. Keller[20,21,22], Ottavia M. Delmonte[19], Lisa R. Forbes Satter[23], Steven M. Holland[19], Jenna R. E. Bergerson[19], Jennifer W. Leiding[24,25], Neil Romberg[1,2,3], Will Bailis[26, 27], Christopher A. Hunter[28], Alexandra F. Freeman[19], Alejandro V. Villarino[5,6], Mark S. Anderson[29,30], Megan A. Cooper[8,9], Tiphanie P. Vogel[31], and Sarah E. Henrickson[1,3,32*]

Supplemental Methods

Mass cytometry. Antibodies for mass cytometry (Supplemental Table 4) were conjugated in-house (using unlabeled, purified antibodies and conjugating them to isotope-loaded polymers with MAXPAR kits (Fluidigm)) or purchased as pre-conjugated metal-tagged antibodies from Fluidigm. After titration was performed, antibodies were diluted using antibody stabilization buffer (Candor Bioscience). Maleimido-mono-amine-DOTA (Macrocyclics) was dissolved in MAXPAR L-Buffer (Fluidigm) and mixed with 139La (L/D-139) for a 50 mM concentration. Cryopreserved PBMCs were thawed into warm complete RPMI (cRPMI: RPMI with 10% fetal bovine serum (FBS), 1% Penicillin/Streptomycin, 1% L-Glutamine and 1% HEPES) and plated at $1x10^6$ live PBMC/subject/well into 96-well U-bottom tissue culture plates. Cell suspensions were pelleted and incubated with 1.5-2.0 μM L/D-139 for 5 minutes at room temperature (RT) for Live/Dead discrimination. Cells were then washed (520xg, 5 min, RT) in staining buffer (1% FBS in PBS) and resuspended in 50μL surface antibody cocktail for 30 minutes at RT. They were then washed twice in staining buffer, fixed, and permeabilized in 50μL Fixation/Permeabilization solution (Foxp3 Transcription Factor Staining Buffer Set, eBioscience) and washed with Permeabilization Buffer (650xg, 5 min, RT). Samples were stained intracellularly (50μL intracellular antibody cocktail in Perm Buffer, 60 min, RT). Cells were washed three times (650xg, 5 min, RT) with Perm Buffer before fixation in 1.6% paraformaldehyde (PFA) (Electron Microscopy Sciences) in PBS overnight with 125nM Iridium (Fluidigm) at 4°C. Cells were washed twice in PBS (no Ca^{2+} or Mg^{2+}) and then washed twice in deionized H_2O prior to acquisition. Samples were acquired on a CyTOF2 (Fluidigm) with bead-based normalization. During data export, bead events were removed. FCS files were analyzed using FlowJo v10 (TreeStar).

Antibody staining for flow cytometry. Approximately 0.25-0.5x10^6 PBMCs per patient per stain were plated in a 96-well U-bottom plate (see Supplemental Table 5 for spectral flow cytometry antibody information). PBMCs were washed once with PBS and stained with either Live/Dead Aqua or Live/Dead Blue and Fc Block mix (50µL, 15 min, 4°C) in PBS. PBMCs were washed with PBS, and spun down (800xg, 4 min, RT). The pellet was resuspended in 50µL of surface stain antibody mix in FACS buffer (PBS containing 1% FBS, 2mM EDTA) plus 10% BD Brilliant Stain Buffer and then incubated at 4°C for 30 minutes. PBMCs were washed with FACS buffer, centrifuged, and the pellet was resuspended in 125µL of FACS buffer for acquisition. For intracellular cytokine and transcription factor staining, the surface stain mix was washed off, and samples were fixed and permeabilized by incubating in 50µL of Fix/Perm buffer (20 min, RT in the dark) and washed in Perm Buffer. PBMCs were stained using 50µL of intracellular antibody mix (diluted in Perm Buffer) for 1 hour at 4°C. After the incubation, samples were washed with Perm Buffer and fixed in 125µL of 1.6% PFA in PBS at 4°C overnight. The following day, samples were acquired.

Intracellular cytokine detection. Freshly thawed PBMCs from patients and HC (0.5x10^6) were seeded into 96-well U-bottom plates in 100µL of cRPMI. Cells were left unstimulated in the presence of 100µL BD GolgiPlug (containing Brefeldin A) and BD GolgiStop (containing Monensin) or were stimulated with 100µL PMA (100ng/mL) and Ionomycin (1µg/mL) with GolgiPlug and GolgiStop for 4 hours. To measure intracellular cytokines, surface and intracellular staining was performed as detailed above.

Olink plasma proteomics. For HC and patients with STAT3 GOF, heparinized plasma (100µL/participant) was sent to Olink Proteomics (Uppsala, Sweden). Identification and measurement of proteins was performed with the Olink Explore 1536 or Olink Explore 384 Inflammation proximity extension assay platforms. Concentrations of proteins are provided as Log2-scaled Normalized Protein eXpression (NPX) units post-normalization and quality control (50, 75).

Cytokine-mediated pSTAT1 and pSTAT3 phospho-flow assay. Cryopreserved PBMCs were thawed as described above, counted, and stained with Fc Block, Live/Dead Aqua and surface stain antibodies combined in FACS Buffer containing 10% BD Brilliant Stain Buffer for 30 minutes at RT in the dark (50µL/1x10^6 cells). Cells were washed and allowed to rest for 1hr at 37°C, 5% CO_2 in plain RPMI. Cells were counted and resuspended at 5x10^6 cells/mL in plain RPMI. Then 50µL (0.25x10^6 PBMC) were plated in a 96-well U-bottom plate and rested for 30 min at 37°C, 5% CO_2. Cytokine solutions were prepared in plain RPMI and kept on ice. 50µL of plain RPMI or indicated cytokine solution was added to the cells for 20 minutes at 37°C, 5% CO_2. Cells were immediately fixed with 100µL of 4% PFA (diluted in PBS) for 15 minutes at RT (2% PFA final in-well concentration). The plate was centrifuged, and the cells were resuspended in 150µL of ice-cold methanol and stored at -20°C overnight for permeabilization. The following morning, cells were centrifuged and washed 2X with 150µL of Perm Buffer followed by staining for phospho-proteins in Perm Buffer for 1hr at RT (50µL/well). After the incubation, samples were washed with Perm Buffer, pelleted, and resuspended in 125µL of 1.6% PFA (in PBS) for fixation. Samples were acquired the next day.

Induction of purinergic molecules in CD8⁺ T cells. 96-well flat bottom plates were coated with 1µg/mL anti-CD3 solution in sterile PBS (200µL) overnight at 4°C. Cryopreserved $CD8^+$ T cells were thawed as described above and plated at $0.2x10^6$ live cells/well (50µL) in cRPMI after aspirating the coating solution, gently washing wells with 200µL PBS, and aspirating the PBS wash. 1µg/mL (final concentration) soluble anti-CD28 or media (50µL) was added per well then either 50µL media or cytokine media (to achieve the final indicated concentrations) were added. Finally, 50µL media or STATTIC (0.5µM final concentration per well) was added for a final in-well volume of 200µL/well. Cells were incubated for 4 days at 37°C, 5% CO_2 before surface staining and intracellular staining as described above. For experiments involving phospho-flow cytometry, after the end of the incubation, 150µL of supernatants was removed, and cells were washed in PBS and then transferred to a 96-well U-bottom plate and pelleted. Cells were resuspended in a mixture of Fc Block, Live/Dead discrimination dye, and surface stain antibodies in FACS Buffer containing 10% BD Brilliant Stain Buffer for 30 minutes at RT (50µL/well). Surface stain was removed by washing with 150µL of FACS buffer and pelleting cells. Cell pellets were fixed by resuspending in 100µL of 2% PFA diluted in PBS for 15 minutes at RT. Cells were then pelleted and resuspended in 150µL of ice-cold methanol and permeabilized at -20°C overnight. Completion of intracellular phospho-protein detection was performed as described above.

For assays prolonged in vitro culture assays, three parallel plates were plated (one for Day 4, Day 10, and Day 14). On Day 4, the Day 4 plate was harvested and surface stained as described above while Day 10 and Day 14 plates were centrifuged and half-volume media or media + cytokine exchange was performed. On Day 10, the Day 10 plate was harvested and surface stained as

described above the Day 14 plate was centrifuged and half-volume media or media + cytokine exchange was performed. On Day 14, the Day 10 plate was harvested and surface stained.

Cell sorting. Cryopreserved PBMCs were thawed as described above and 0.5 to $1x10^6$ cells were transferred to a new tube for washing with PBS. After PBS wash and centrifugation, cell pellets were incubated in 50µL of Live/Dead Aqua, CD14/CD16/CD19 V500, CD3 APC-R700, CD4 AF488 and CD8a BV605 antibodies in FACS Buffer with 10% Brilliant Stain Buffer for 30 minutes at RT. Cells were washed with FACS Buffer and pellets were resuspended in 500µL FACS Buffer and filtered through a nylon mesh cell strainer (35µm) to remove clumps, debris, and prevent clogging. Live, single $CD14^-CD16^-CD19^-CD3^+CD4^-CD8^+$ cells were sorted into 1.5mL Eppendorf tubes containing RPMI + 10% FBS on a Cytek Aurora CS System (Cytek Biosciences, Fremont, CA). Removal of EDTA was achieved by washing sorted cells two times with RPMI + 10% FBS. Cells were counted prior to 10X processing for single-cell RNA sequencing.

Single cell RNA sequencing (scRNA-seq) analysis. Single cell analyses of sorted $CD8^+$ T cells from PBMC samples from human participants (HC and STAT3 GOF) were performed using the 10X Chromium Next GEM Single Cell 5' Kit v2 and Chromium Single Cell Human TCR Amplification Kit (10X Genomics, Pleasanton, CA). We worked with the Center for Applied Genomics at Children's Hospital of Philadelphia for single-cell isolation and library preparation. An Illumina S2 flow cell (Illumina, San Diego, CA) was used for sequencing. Sequencing basecall files were demultiplexed with 10x Genomics Cell Ranger v6.1.2's makefastq (76). Gene expression fastq files were mapped to the human genome (GRCh38) and TCR fastq files were mapped to the CellRanger V(D)J reference (GRCh38, ensembl 7.1.0) using 10x Genomics Cell Ranger v7.1.0's

multi (76). Cellranger mapped h5 files were read in to and processed in R using Seurat v4 (77). Samples were assessed for quality and cells with less than 500 total reads, 300 total features, or more than a 5% mitochondrial percentage were filtered out. Doublets were also detected and removed with the DoubletFinder package (78). After quality control, 94,279 cells were further analyzed. Cells from each sample were integrated together using the top 3000 most variable features and Seurat v4's IntegrateData function (77). The integrated Seurat object was clustered with Seurat's FindNeighbors and FindClusters functions (77). Resolutions ranging from 0.05 to 0.9 were assessed with clustree (44). A resolution of 0.3 was optimal and resulted in 10 clusters. These ten clusters were then annotated using a mixture of automated and manual methods. First, cluster annotations were defined using SingleR with the Monaco Immune Cell Data reference dataset from the celldex package, giving an initial set of annotations (45, 46). The clusters were further examined using a select set of markers. One cluster was classified as monocytes due to high levels of *CD14* and was filtered out. Another cluster was identified as low quality $CD8^+$ T cells (low nCounts, nFeatures, high mitochondrial percentage) and filtered out for a total of 8 clusters of $CD8^+$ T cells. The rest of the annotations were defined based on the initial Monaco annotation and the expression of the markers of interest. Naïve clusters (C1 and C2) were defined based on increased expression of *CCR7*, *SELL*, *LEF1*, *TCF7*, *IL7R* and absence of expression of effector and activation genes including *CCL5*, *CXCR3,* and *TBX21*. The terminal effector cluster (C3) was defined based on increased *GZMB, KLRB1, CCL5, TBX21, PRDM1* and absent expression of genes present in C1 and C2. The. effector memory cluster (C4) was defined by increased expression of *CCR7*, *TCF7*, *IL7R*, *GZMB, CCL5, CXCR3, TBX21* while the effector cluster (C7) had increased expression of *FAS*, *GZMB*, *KLRB1*, *KLRK1*, *CCL5*, *CXCR3*, *TBX21* and *PRDM1*. The central memory cluster (C6) had increased expression of *CD27*, *CCR7*, *TCF7*, *IL7R*,

CCL5 and decreased expression of *TBX21*, *CXCR3* and *PRDM1* while the stem cell memory cluster (C8) demonstrated presence of C1 and C2 genes along with increased expression of *FAS, CXCR3* and effector genes *KLRK1*, *CCL5*, *TBX21,* and *FOXO1*. The last cluster (C5) was annotated as MAIT cells due to expression of *KLRB1*, *IL7R* and *DPP4*. Propeller in the speckle package was used to assess independence of the cell type annotations and patient disease status (47). Remaining $CD8^+$ T cells were examined without the MAIT cell cluster. Genes in STAT3 GOF patients and HCs were compared in the $CD8^+$ T cells using Seurat v4's FindMarkers function with the Wilcoxson method being the test used. Differential expression among the genes was conducted by first pseudobulking cell counts per individual sample using Seurat's AggregateExpression function (77). EdgeR was used to process the data and run the differential expression analysis (79). Counts were also normalized and log2 scaled using edgeR's cpm function to look for potential gene set enrichment using the Broad Institute and UC San Diego's GSEA (79). The Human MSigDB v2023.2.Hs Hallmark gene sets were examined in the STAT3 GOF patients and HC (48, 49). Single-cell related figures including the dimension plot, dotplot, and volcano plots were made with a combination of built in Seurat functions and ggplot2 (77, 80).

T cell receptor analysis. TCR-seq data was initially mapped with the rest of the data during using Cellranger (76). The resulting filtered contig annotations per cell were incorporated into the data using the scRepertoire package (v.2.4.0) (81). ScRepertoire was also used to calculate shannon diversity information per sample and gather clonal proportions per sample for the sequenced T cell receptors. Visualizing TCRseq was done using a mixture of the Seurat package, ggplot, and scRepertoire (77, 80, 81). Diversity and clonal expansion were compared between disease groups using a wilcox test. Finally, T cell receptors were mapped to multiple TCR databases: VDJdb (82),

McPAS-TCR (83), iedb (84), and TBA (85), using the Trex package (0.99.12) (86). T cell receptors were mapped to these databases using the TCR beta chains, and up to two amino acids were allowed to be different from the database sequences.

Chromatin immunoprecipitation sequencing re-analysis. ChIP-seq datasets for STAT1 and STAT3 were downloaded from the NCBI Gene Expression Omnibus (GSE65621) (59). We have included data for $CD4^+$ T cells cultured in vitro with or without IL-27. 50 base pair reads were aligned using bowtie and macs2 (default settings) was used to map non-redundant reads to mouse genome (mm9). Input controls were used as references for peak calling. Homer was used for peak annotation and DNA motif enrichment analysis. We used bedtools intersect for peak overlap analysis (-wa option). Gene proximal peaks were defined as occurring within introns, exons, being less than 10 kb from transcriptional start or end sites or in untranslated regions. IGV was used to render genome browser files.

PBMC inhibition with ATP and 2-CADO. Live PBMCs (0.15×10^6, 50µL) were plated per well in a flat-bottom 96-well plate pre-coated with 200µL of 1µg/mL anti-CD3 in PBS. Media or soluble anti-CD28 solution (50µL, final concentration = 1µg/mL) was added alongside 100µL media, ATP (final concentration = 50µM) or 2-choloroadenosine (2-CADO, final concentration = 5µM). Cells were incubated for 24 hours then supernatants were harvested and frozen, followed by addition of Brefeldin A/Monensin for an additional 4 hours. Then, surface and intracellular cytokine staining was performed as described above. Percent change in cytokine production in αCD3/αCD28 ± ATP or 2-CADO conditions was calculated as follows:

$$\% \text{ Change in IFN-}\gamma^+ = ((\%\text{IFN-}\gamma^+_{\text{CD3/CD28 + ATP or 2-CADO}} - \%\text{IFN-}\gamma^+_{\text{CD3/CD28}})/(\%\text{IFN-}\gamma^+_{\text{CD3/CD28}}))*100$$

Mouse splenocyte processing. Spleens were harvested from indicated mouse strains and shipped overnight in R10 media (RPMI + 10% FBS + 1% Penicillin/Streptomycin and 1% L-Glutamine) on ice packs. On arrival, tissues were weighed and mechanically dissociated by passing through a 70µm cell strainer with the back of a 1mL syringe. The strainer was regularly flushed with cold FACS buffer. Cells were pelleted at 4°C (1600 RPM x 5 min) and resuspended in 4mL of ACK Lysis Buffer, vortexed, and incubated for 8 min to lyse red blood cells, followed by quenching with 16mL of R10 media. Cells were counted with Nexcelom Cellometer K2 Image Cytometer Automated Cell Counter and after pelleting, cells were resuspended at $1x10^7$ live splenocytes/mL in mouse cRPMI (RPMI + 10% FBS, 1% Penicillin/Streptomycin, 1% L-Glutamine, 1% HEPES, 1% Non-Essential Amino Acids and 50µM β-ME). 100µL of cells were used per mouse per assay.

Retroviral transduction. cDNA encoding WT (ENSMUST00000127638.8) or mutant (p.R152W or p.Q344H) mouse Stat3 were cloned into an MoMLV-based plasmid vector (MigR1) directly in upstream of the internal ribosome entry sequence (IRES) and GFP marker. Plasmid vectors were then transfected into 293T cells along with pCL-Eco helper plasmid (87). Supernatants containing virus were harvested after 48 hours. In parallel, naive $CD44^{Low}CD25^{-}CD4^{+}$ or $CD8^{+}$ T cells were sorted (purity > 95%) from pooled spleen and lymph nodes of *Stat3* flox/flox CD4-Cre(+) and *Stat3* flox/flox CD4-Cre(-) mice. Cells were activated with plate-bound anti-CD3e/CD28 (10µg/mL each) for 24 hours and then incubated with viral supernatant for 1 hour (2200 rpm, 18°C). Following transduction, cells were cultured in vitro for a further 48 hours with recombinant murine IL-27 (10ng/mL), anti-mouse IL-4 (10µg/mL) and anti-mouse IFN-g (10µg/mL), then

stained for flow cytometry. Cytometry data were collected on an Aurora instrument (Cytek Biosystems).

Flow cytometry. Samples were acquired on a five-laser Cytek Aurora at the Children's Hospital of Philadelphia Flow Cytometry Core, except for the retroviral research, which was performed at the Flow Cytometry Shared Resource of the Sylvester Comprehensive Cancer Center at the University of Miami (RRID: SCR022501). SpectroFlo® QC Beads were used for routine performance tracking. UltraComp eBeads were used for compensation. Data were analyzed using FlowJo software (version 10.9.0, Tree Star, Ashland, OR).

Reagents and Resources. Additional information on key reagents/resources are listed in Supplemental Table 6.

Supplemental Figures

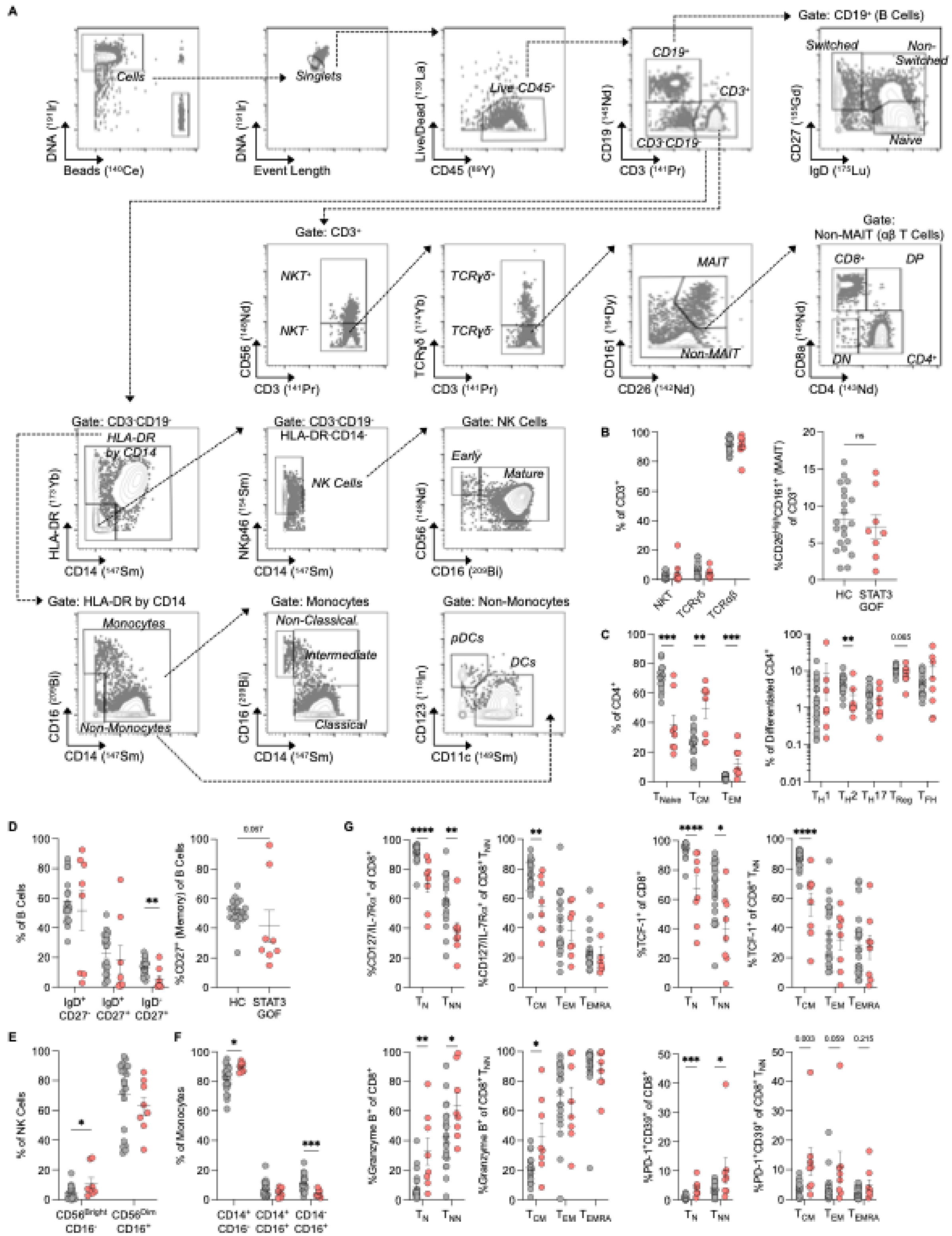

Supplemental Figure 1: Gating strategies and additional characterization of immune cell perturbations in STAT3 GOF in CD4$^+$ T cells, NK cells, B cells, and Monocytes. (A) Mass cytometry (CyTOF) gating strategy and frequencies of major immune cell populations in untreated STAT3 GOF patients (n = 8) and age-matched HC (n = 21). **(B)** Frequencies of NKT (CD3$^+$CD56$^+$), TCRγδ (CD3$^+$CD56$^-$TCRγδ$^+$), TCRαβ (CD3$^+$CD56$^-$TCRγδ$^-$) and MAIT (CD3$^+$CD26$^+$CD161$^+$) cells. **(C)** Differentiation of CD4$^+$ T cells and frequencies of non-naïve CD4$^+$ T helper cell subsets as indicated.: T_H1: T-bet$^+$GranzymeB$^+$, T_H2: CD26^{Int}CRTH2$^+$, T_H17: CD26^{High}CRTH2$^-$, T_{REG}: CD127^{Low}FOXP3$^+$ and T_{FH}: PD-1^{High}CXCR5$^+$. **(D)** Frequencies of B cell populations: naïve: IgD$^+$CD27$^-$, non-switched memory: IgD$^+$CD27$^+$, switched memory: IgD$^-$CD27$^+$ and total memory (CD27$^+$) cells. **(E)** Frequencies of NK cell populations: immature: CD56^{Bright}CD16$^-$ and mature: CD56^{Int}CD16$^+$. **(F)** Frequencies of monocyte populations: non-classical: CD16$^+$CD14$^-$, intermediate: CD16$^+$CD14$^+$, and classical: CD16$^-$CD14$^+$. **(G)** Frequency of CD127$^+$, TCF-1$^+$, Granzyme B$^+$ and PD-1$^+$CD39$^+$ cells within naïve (T_N) or non-naïve (T_{NN}) CD8$^+$ T cells (left plot of each pair) and within non-naïve subsets (T_{CM} vs. T_{EM} vs. T_{EMRA}; right plot of each pair). Data represent mean ± SEM and are pooled from 3 independent experiments. *$P \leq 0.05$, **$P \leq 0.01$, ***$P \leq 0.001$ by Mann-Whitney Test. Not listed or ns was not statistically significant.

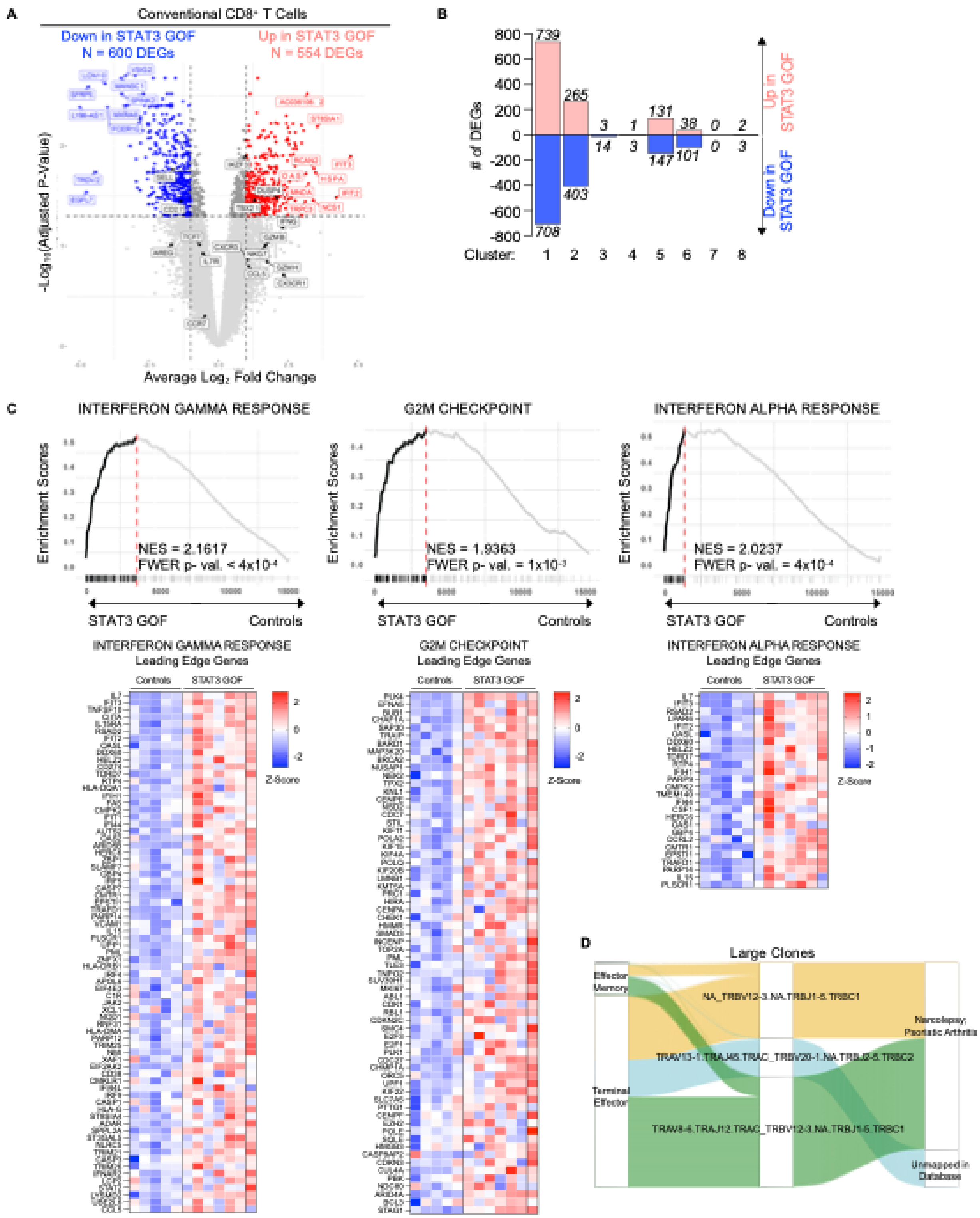
A
Conventional CD8+ T Cells
Down in STAT3 GOF
N = 600 DEGs
Up in STAT3 GOF
N = 554 DEGs
-Log10(Adjusted P-Value)
Average Log2 Fold Change
B
# of DEGs
Up in STAT3 GOF
Down in STAT3 GOF
Cluster: 1 2 3 4 5 6 7 8
C
INTERFERON GAMMA RESPONSE
NES = 2.1617
FWER p- val. < 4x10-4
G2M CHECKPOINT
NES = 1.9363
FWER p- val. = 1x10-3
INTERFERON ALPHA RESPONSE
NES = 2.0237
FWER p- val. = 4x10-4
Enrichment Scores
STAT3 GOF
Controls
INTERFERON GAMMA RESPONSE Leading Edge Genes
G2M CHECKPOINT Leading Edge Genes
INTERFERON ALPHA RESPONSE Leading Edge Genes
Z-Score
D
Large Clones
Effector Memory
Terminal Effector
NA_TRBV12-3.NA.TRBJ1-5.TRBC1
TRAV13-1.TRAJ45.TRAC_TRBV20-1.NA.TRBJ2-5.TRBC2
TRAV8-6.TRAJ12.TRAC_TRBV12-3.NA.TRBJ1-5.TRBC1
Narcolepsy; Psoriatic Arthritis
Unmapped in Database

Supplemental Figure 2: Differentially expressed genes and GSEA leading edge genes in STAT3 GOF. (A) Volcano plot demonstrating top 10 most upregulated (red, labeled), downregulated (blue, labeled) and select genes (black, labeled) in conventional STAT3 GOF $CD8^+$ T cells. **(B)** Bar plot showing number of differentially expressed genes upregulated or downregulated in STAT3 GOF $CD8^+$ T cells from scRNA-seq analysis by cluster. **(C)** Enrichment plots and heatmaps/annotation of leading-edge genes (ordered by Rank Metric Score) of statistically significant pathways enriched in STAT3 GOF. **(D)** Alluvial plot of large clones detected in 1 patient with STAT3 GOF: the leftmost column represents clusters contributing to large clone populations, the middle column represents the TCR usage, and the rightmost column represents the epitope species. Two clonotypes (yellow and green) are both mapped to Psoriatic Arthritis and Narcolepsy using the Trex package and share a single TCR beta chain. while the third clonotype (blue) is not mapped to a specific antigen in any of the tested public databases.

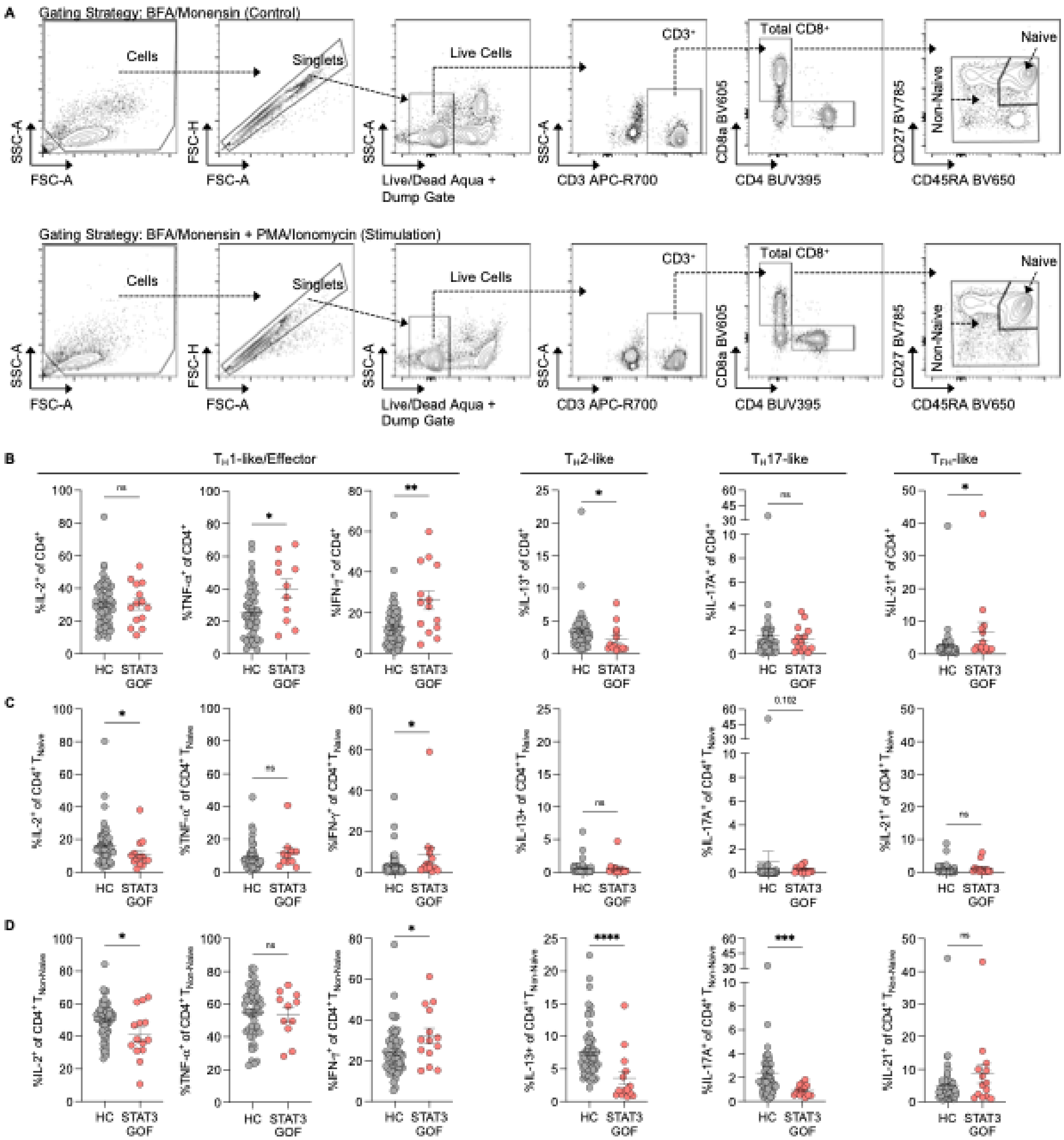

Supplemental Figure 3: Gating strategy for intracellular cytokine detection and quantification of CD4$^+$ T cell cytokine production. **(A)** Gating strategy for spectral flow cytometry intracellular cytokine detection/quantification in the presence of BFA/Monensin ± PMA/Ionomycin. Naïve CD8$^+$ T cells are defined as CD45RA$^+$CD27$^+$ while non-naive cells

(antigen-experienced) are defined as non-$CD45RA^+CD27^+$ (L-shape encompassing $CD45RA^-CD27^+$, $CD45RA^-CD27^-$, and $CD45RA^+CD27^-$). Quantification of T_H1-like (IFN-γ, TNF-α, and IL-2), T_H2-like (IL-13), T_H17-like (IL-17A), and T_{FH}-like (IL-21) cytokine production by **(B)** total, **(C)** naïve, and **(D)** non-naïve $CD4^+$ T cells. For **B**-**D**: data are pooled from 7 to 8 independent experiments with HC (n = 50-58) and untreated patients with STAT3 GOF (n =11-14). Data represent mean ± SEM and $*P \leq 0.05$, $**P \leq 0.01$, $***P \leq 0.001$, $****P \leq 0.0001$ by Mann-Whitney Test.

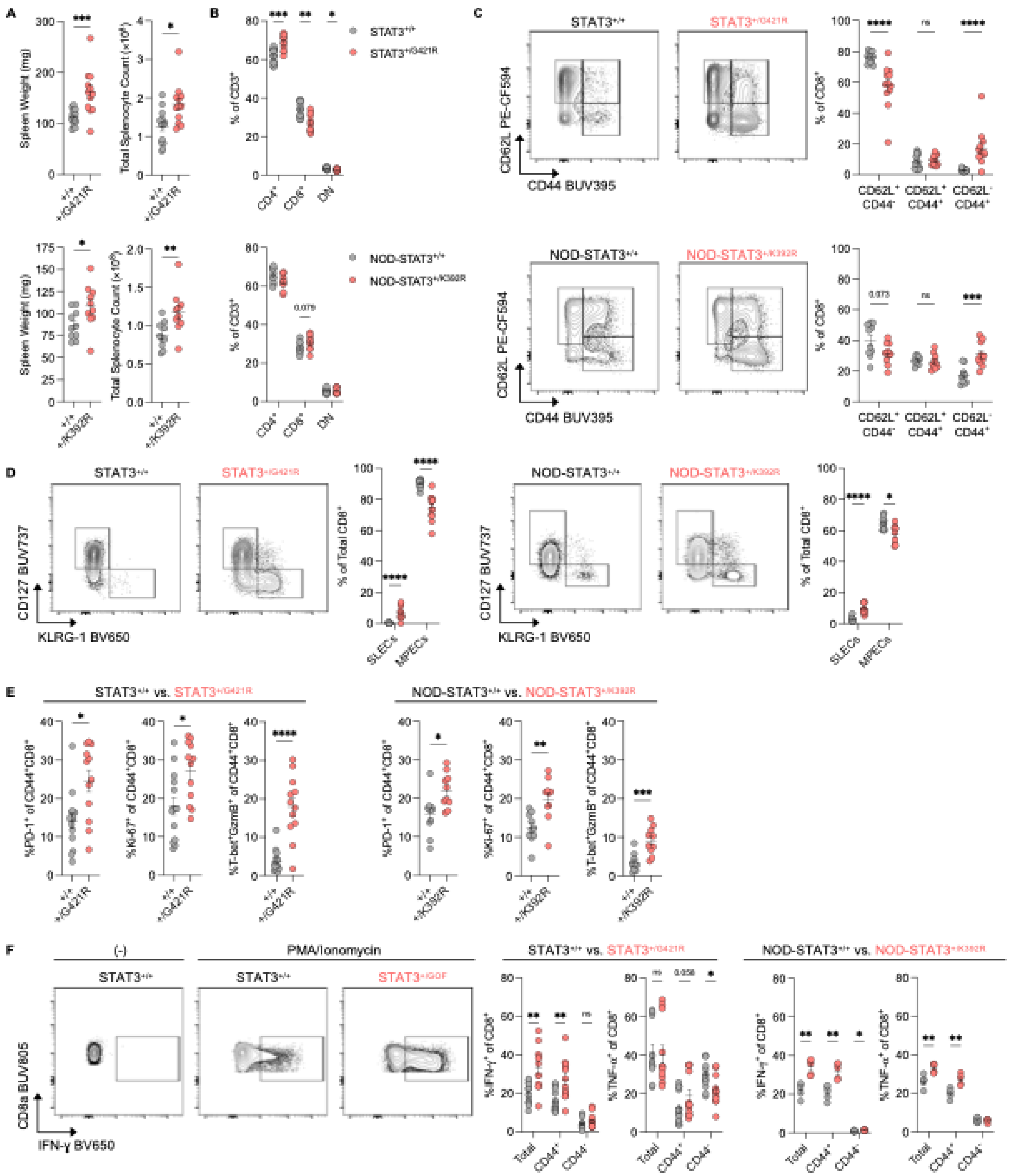

Supplemental Figure 4: Additional characterization of the CD8$^+$ T cell compartment in STAT3$^{+/G421R}$ and NOD-STAT3$^{+/K392R}$ mouse models. (A) Comparison of spleen weight and total cell numbers in the spleen of STAT3$^{+/+}$ vs. STAT3$^{+/G421R}$ (top row) and NOD-STAT3$^{+/+}$ vs. NOD-

$STAT3^{+/K392R}$ mice (bottom row). **(B)** Comparison of $CD3^+$ subsets in the spleen of $STAT3^{+/+}$ vs. $STAT3^{+/G421R}$ (top) and NOD-$STAT3^{+/+}$ vs. NOD-$STAT3^{+/K392R}$ (bottom) mice. **(C)** Representative $CD8^+$ T cell subsets and quantification in $STAT3^{+/+}$ vs. $STAT3^{+/G421R}$ and NOD-$STAT3^{+/+}$ vs. NOD-$STAT3^{+/K392R}$ mice. **(D)** Representative memory precursor effector cell (MPEC; $CD127^+KLRG\text{-}1^-$) and short-lived effector cell (SLEC; $CD127^-KLRG\text{-}1^+$) populations and quantification in $STAT3^{+/+}$ vs. $STAT3^{+/G421R}$ and NOD-$STAT3^{+/+}$ vs. NOD-$STAT3^{+/K392R}$ mice. **(E)** Quantification of activation markers on $CD44^+CD8^+$ T cells in $STAT3^{+/+}$ vs. $STAT3^{+/G421R}$ and NOD-$STAT3^{+/+}$ vs. NOD-$STAT3^{+/K392R}$ mice. **(F)** Representative flow cytometry plots of IFN-γ^+ cytokine production by $CD8^+$ T cells after 5 hours with PMA/Ionomycin and percent IFN-γ^+ and TNF-α^+ cells in total $CD8^+$, $CD44^+CD8^+$ and $CD44^-CD8^+$ T cells in $STAT3^{+/+}$ vs. $STAT3^{+/G421R}$ and NOD-$STAT3^{+/+}$ vs. NOD-$STAT3^{+/K392R}$ mice. Assays were performed in total splenocytes and then gated on live $mCD45^+B220^-Gr\text{-}1^-CD3^+CD8^+$ cells. For **A**-**E**: data are pooled from 3 independent experiments for $STAT3^{+/+}$ (n = 14) vs. $STAT3^{+/G421R}$ (n = 12); data are pooled from 2 independent experiments for NOD-$STAT3^{+/+}$ (n = 10) vs. NOD-$STAT3^{+/K392R}$ (n = 10). For **F**: data are pooled from 3 independent experiments for $STAT3^{+/+}$ (n = 14) vs. $STAT3^{+/G421R}$ (n = 12); data is from 1 experiment with NOD-$STAT3^{+/+}$ (n = 5) vs. NOD-$STAT3^{+/K392R}$ (n = 5). Data represent mean ± SEM and $*P \leq 0.05$, $**P \leq 0.01$, $***P \leq 0.001$, $****P \leq 0.0001$ by Mann-Whitney Test.

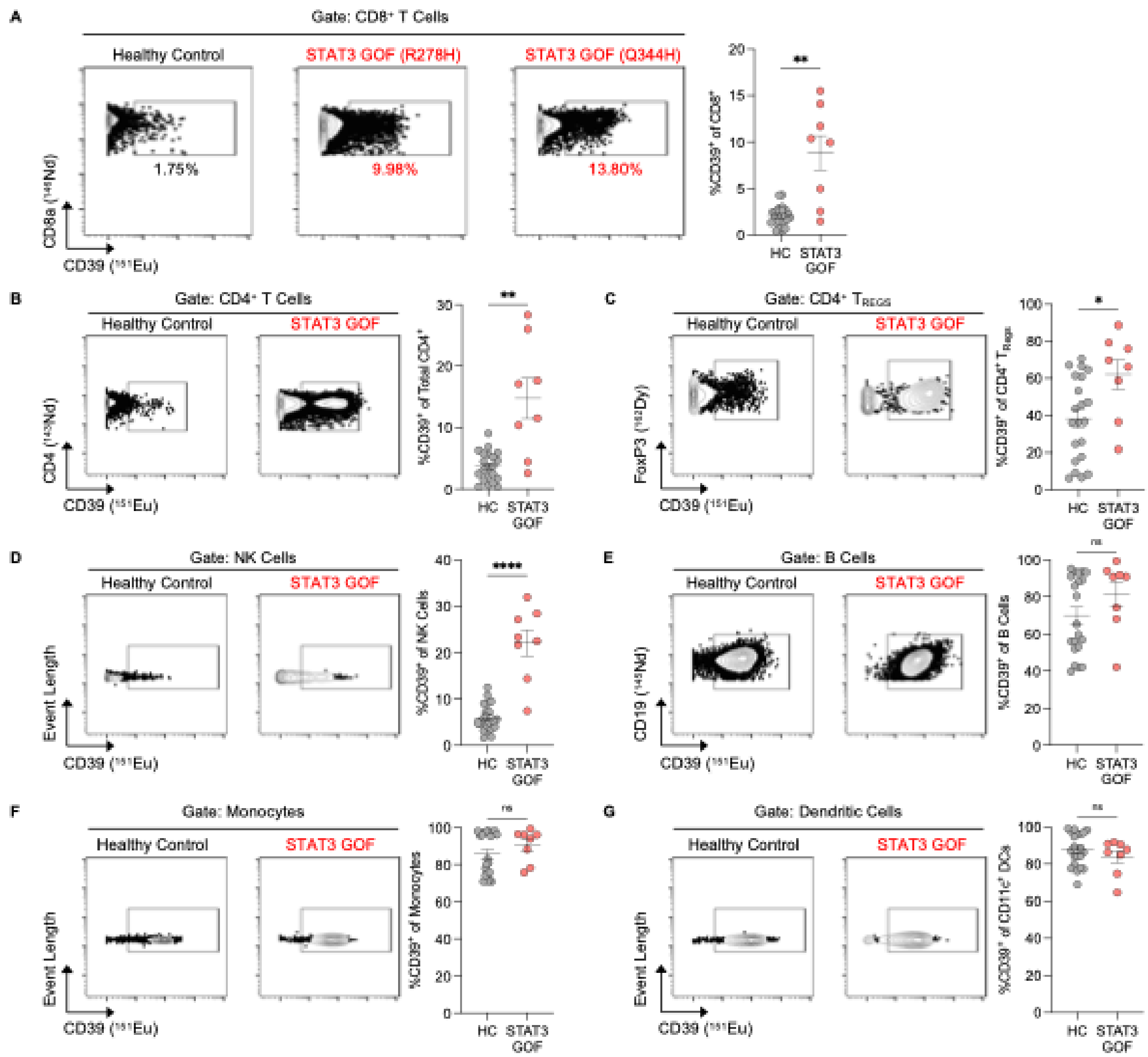

Supplemental Figure 5: Mass cytometry quantification of CD39 expression across immune cell lineages. Representative CD39 plots and quantification of CD39 frequency between HC (n = 21) and patients with STAT3 GOF (n = 8) in: **(A)** CD8$^+$ T cells, **(B)** CD4$^+$ T cells, **(C)** TREGS, **(D)** NK cells, **(E)** B cells, **(F)** monocytes and **(G)** CD11c$^+$ dendritic cells. Gating strategy from Supplemental Figure 1A was used to define lineages. Data are pooled from 3 independent experiments and data represent mean ± SEM. *$P \leq 0.05$, **$P \leq 0.01$, ****$P \leq 0.0001$ by Mann-Whitney Test.

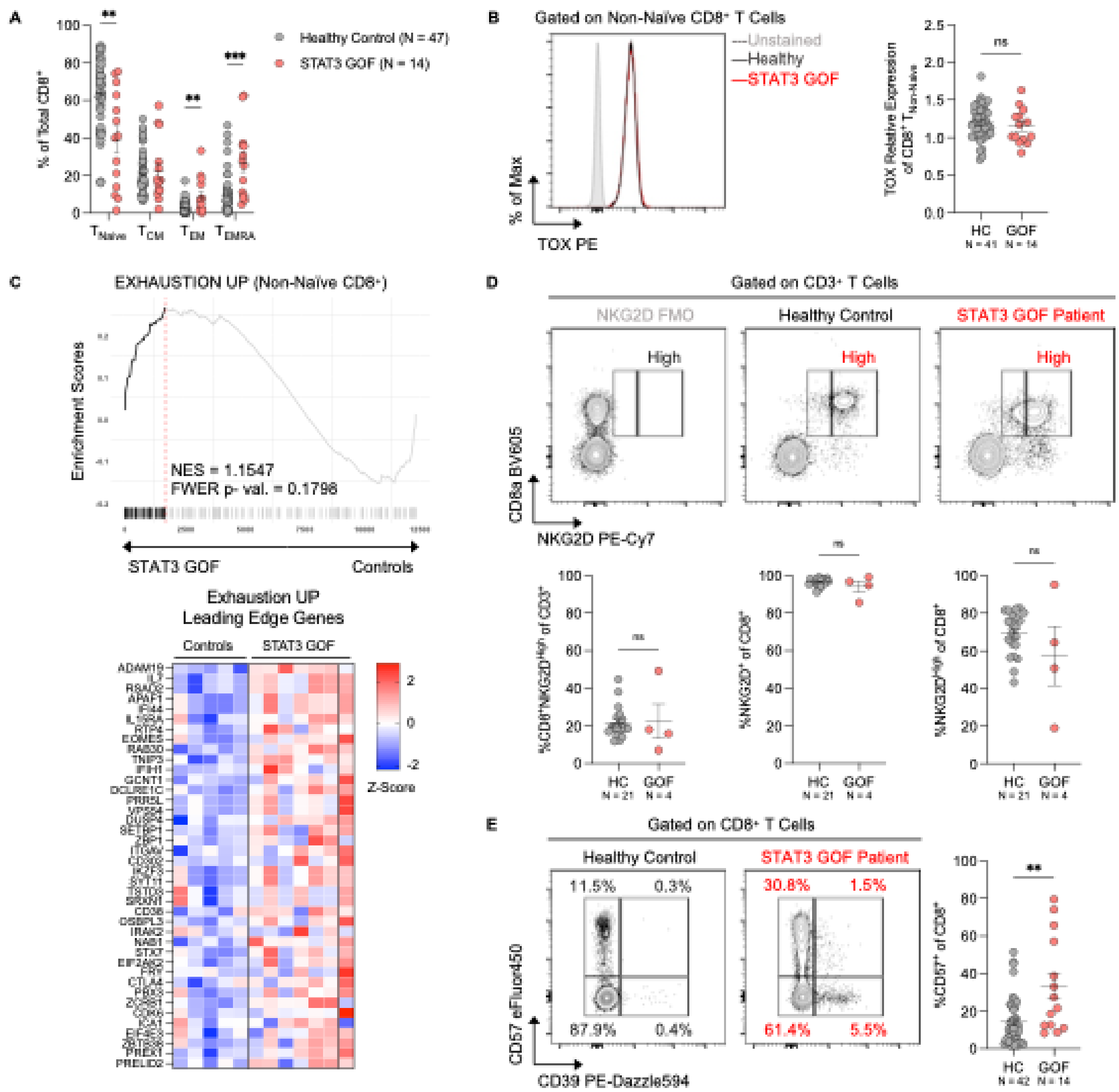

Supplemental Figure 6: Characterization of TOX, NKG2D, and CD57 expression in CD8$^+$ T cells. (A) Frequencies of CD8$^+$ T cell subsets from spectral flow cytometry assays. **(B)** Representative flow cytometry histogram of TOX expression and quantification of relative expression among non-naïve CD8$^+$ T cells compared to a consistently used control in each experiment. **(C)** Enrichment plots and annotation of leading-edge genes (ordered by Rank Metric Score) of T cell exhaustion GSEA on non-naïve CD8$^+$ T cell clusters in STAT3 GOF compared to HC. **(D)** Representative flow cytometry plots and frequencies of NKG2D^{High}CD8$^+$ (within CD3$^+$ T cells) and NKG2D$^+$ and NKG2D^{High} (within CD8$^+$ T cells). **(E)** Representative CD57 and CD39

co-expression flow cytometry plots and CD57 frequency quantification. For **A**-**B, E**: data are pooled from >5 independent experiments. For **D**: data are pooled from 2 independent experiments. Data represent mean ± SEM. $**P \leq 0.01$, $***P \leq 0.001$ by Mann-Whitney Test.

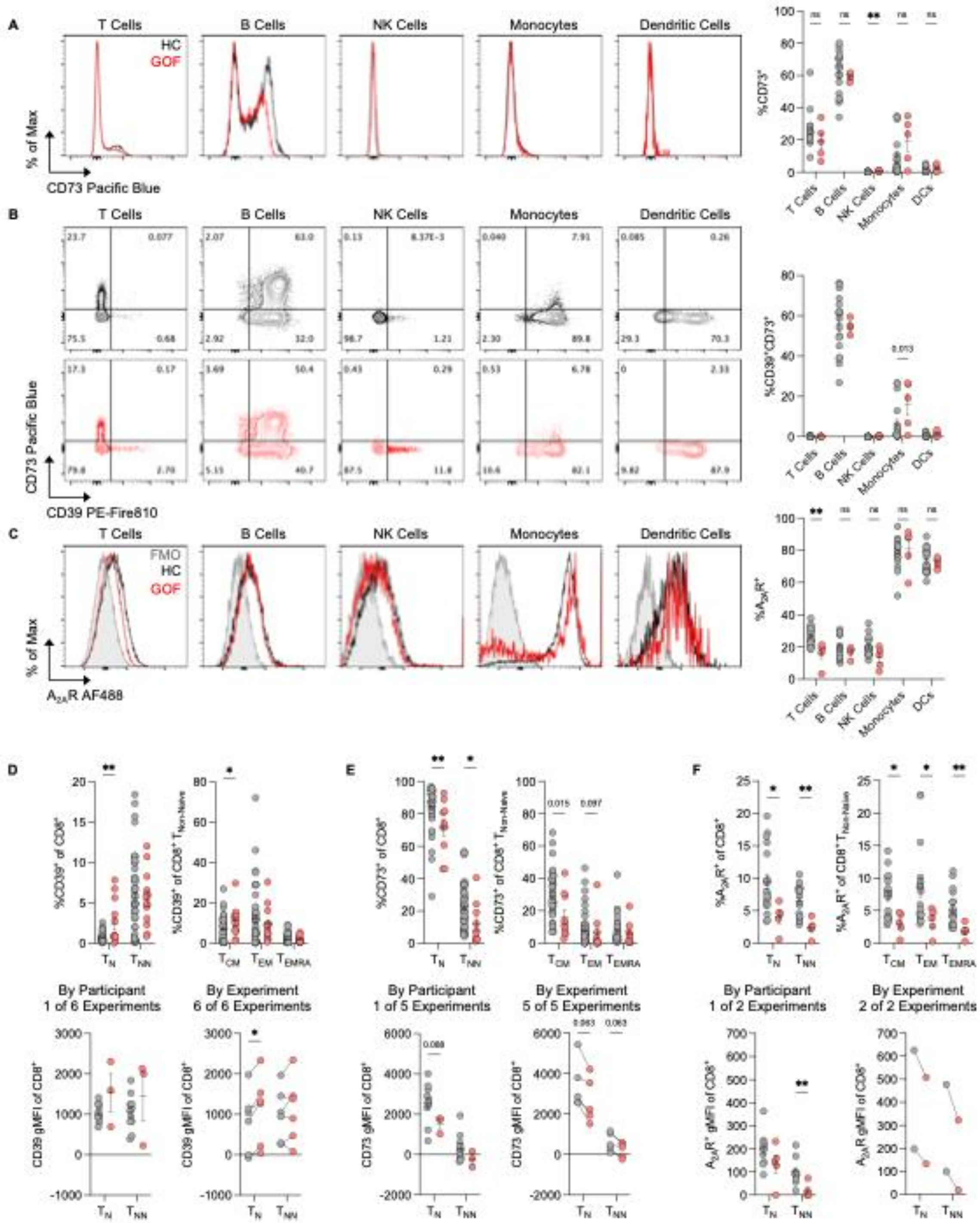

Supplemental Figure 7: CD73 and A2AR expression across STAT3 GOF immune cell lineages, and alterations in expression of CD39, CD73, A2AR in T cell subsets by spectral flow cytometry. **(A)** Representative CD73 histograms and quantification of CD73 positivity across

immune cell lineages by disease state. **(B)** Representative CD39 and CD73 bivariate plots across immune cell lineages and quantification of CD39 and CD73 co-expression. **(C)** Representative $A_{2A}R$ histograms and quantification of $A_{2A}R$ positivity across immune cell lineages by disease state. Top row: **(D)** CD39, **(E)** CD73, and **(F)** $A_{2A}R$ positivity in HC and untreated patients with STAT3 GOF first by naïve (T_N) or non-naïve (T_{NN}) $CD8^+$ T cells (left plots) and then within non-naïve subsets (T_{CM} vs. T_{EM} vs. T_{EMRA}) (right plots) for each marker. Bottom row: **(D)** CD39, **(E)** CD73, and **(F)** $A_{2A}R$ expression (gMFI) in a representative experiment (left plot) and pooled/collapsed data where HC and STAT3 GOF values were averaged into 1 data point which each data point representing a unique experiment (right plots). For **A-C**: HC (n =18) and untreated patients with STAT3 GOF (n = 5). For top row in **D-F**: CD39: HC (n = 47) and untreated patients with STAT3 GOF (n = 14); CD73: HC (n = 42) and untreated patients with STAT3 GOF (n = 10); $A_{2A}R$: HC (n = 18) and untreated patients with STAT3 GOF (n = 5). Data represent mean ± SEM. $*P \leq 0.05$ and $**P \leq 0.01$ by multiple Mann-Whitney Test or Multiple Wilcoxon test (where data are paired) with not listed or ns not being statistically significant.

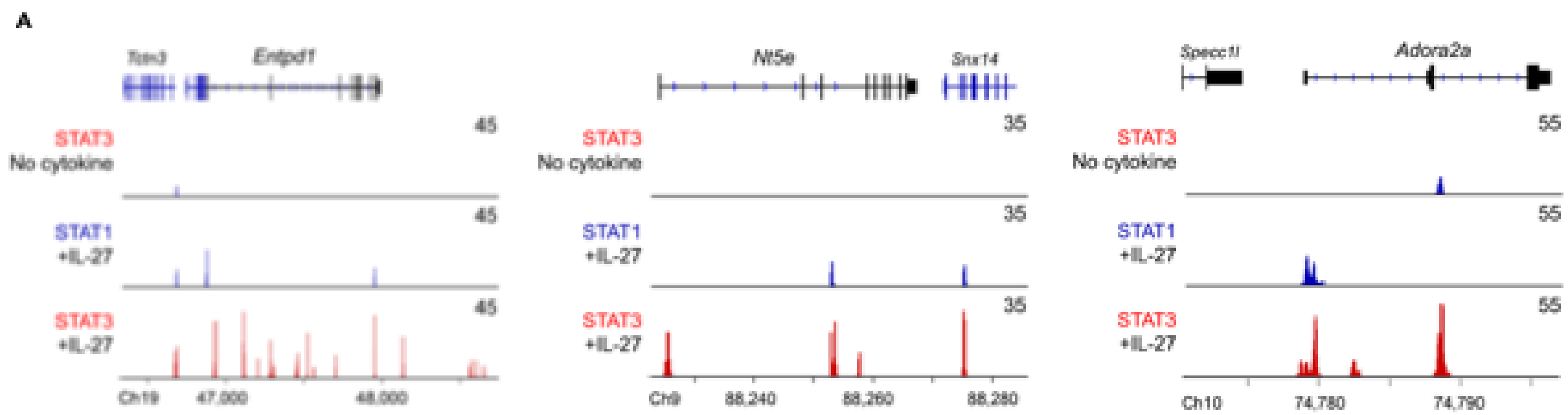

Supplemental Figure 8: ChIP-Seq analysis of STAT3 binding at *Entpd1, Nt5e, and Adora2a* loci in mouse $CD4^+$ T cells. (A) Chromatin immunoprecipitation (ChIP)-Seq analysis of publicly available data demonstrating *Entpd1* (CD39), *Nt5e* (CD73), and *Adora2a* ($A_{2A}R$) as STAT3 targets in mouse $CD4^+$ T cells stimulated with IL-27.

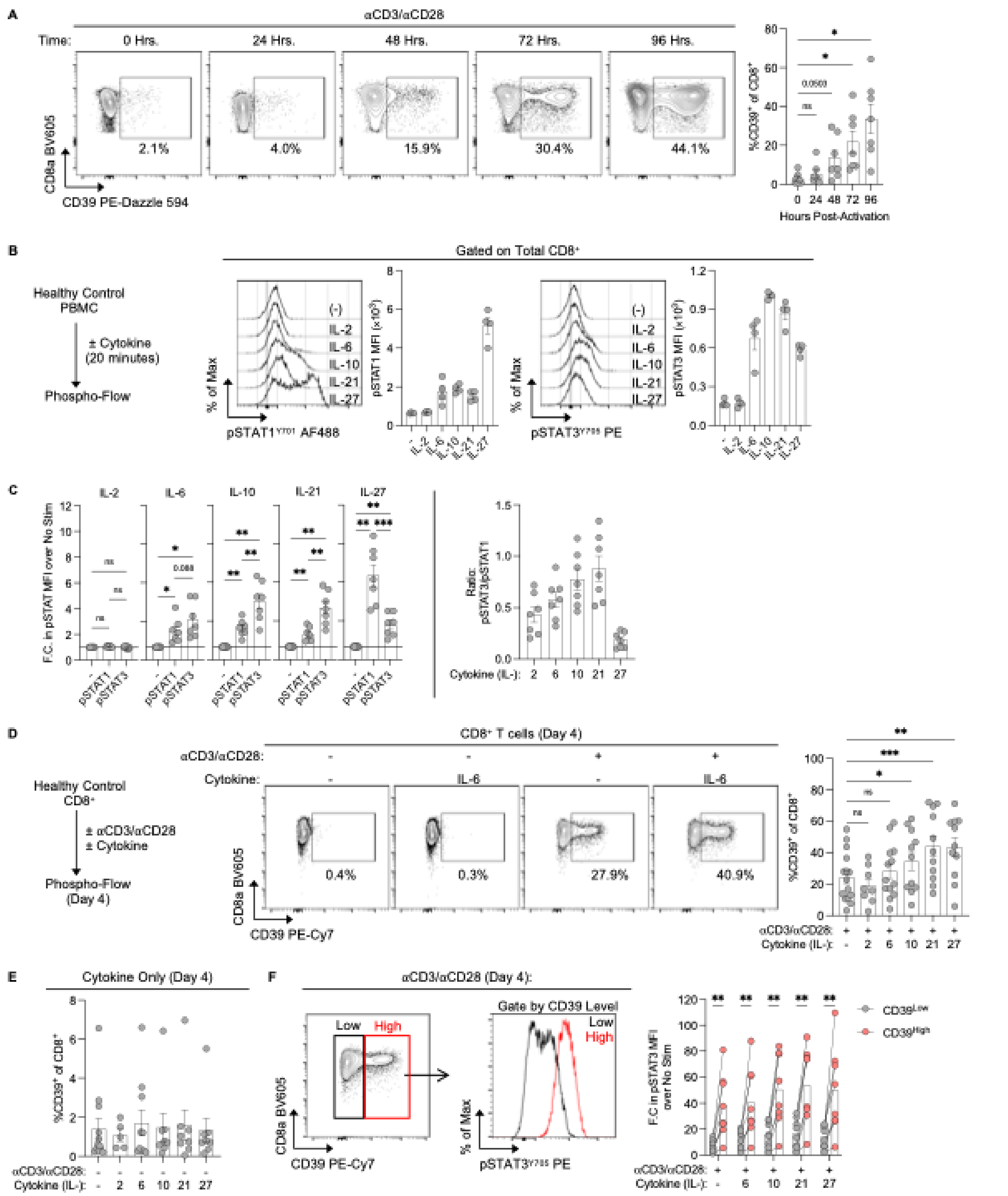

Supplemental Figure 9: T cell receptor engagement and STAT3-activating cytokines synergize to increase CD39 expression in healthy donor CD8$^+$ T cells. (A) Representative flow

cytometry plots of CD39 induction in PBMCs (gated on $CD8^+$ T cells) over 96 hours following αCD3/αCD28 activation and quantification of CD39-positivity (n = 7). **(B)** Characterization of pSTAT1 and pSTAT3 induction in $CD8^+$ T cells by various cytokines (IL-2 = 25IU/mL, IL-6 = 100ng/mL, and IL-10, IL-21 and IL-27 = 25ng/mL) for 20 minutes; data is representative of 1 of 2 experiments. **(C)** Summary of pSTAT1 and pSTAT3 fold change over unstimulated condition and ratio of pSTAT3/pSTAT1 signal from 2 independent experiments (n = 7). **(D)** Representative plots of $CD39^+CD8^+$ T cells left unstimulated, stimulated with IL-6 alone or with αCD3/αCD28 ± IL-6; quantification of CD39-positivity across various αCD3/αCD28 ± cytokine conditions in isolated $CD8^+$ T cells (n > 5). **(E)** Frequencies of $CD39^+CD8^+$ T cells after 96-hour activation with indicated cytokines alone (n > 5). **(F)** Stratification of $CD39^{Low}$ and $CD39^{High}$ $CD8^+$ T cells and $pSTAT3^{Y705}$ intensity after 96-hour activation; quantification of fold change in pSTAT3 levels across CD39 levels in αCD3/αCD28 ± cytokine conditions (n = 8). For **D-E**: assays were performed in technical triplicates, and each data point is the average of those triplicates. Data represent mean ± SEM and $*P \leq 0.05$, $**P \leq 0.01$, $***P \leq 0.001$ by Paired T-test or RM one-way ANOVA with Tukey's multiple comparison test, as appropriate.

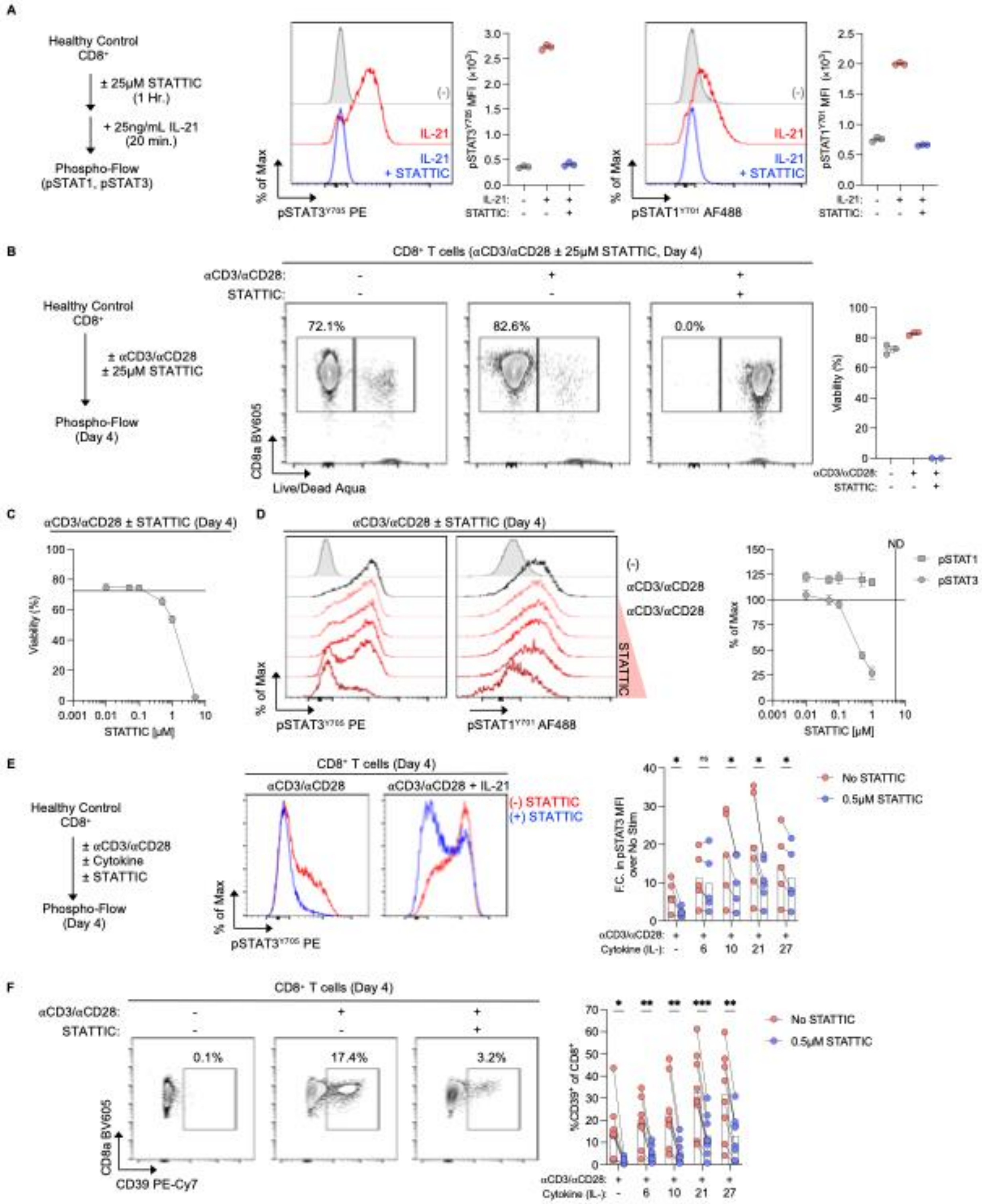

Supplemental Figure 10: In vitro STAT3 inhibition assay development with STATTIC. (A) Schematic for in vitro inhibition of phospho-STAT (pSTAT) signaling with STATTIC (25μM) pre-

treatment for 1 hour followed by stimulation with IL-21. Representative pSTAT3 and pSTAT1 histograms, and quantification of pSTAT levels in $CD8^+$ T cells. **(B)** Study schematic for $CD8^+$ T cells activated with αCD3/αCD28 ± 25μM STATTIC for 96 hours and quantification of viability. **(C)** Viability of cells after 96 hours of activation with αCD3/αCD28 and decreasing amounts of STATTIC; dashed line indicates viability of cells activated with αCD3/αCD28 alone. **(D)** Quantification of percent of max pSTAT3 and pSTAT1 levels in αCD3/αCD28 + STATTIC treated cells relative to αCD3/αCD28 condition alone (dashed line). Representative histograms of **(E)** $pSTAT3^{Y705}$ levels and **(F)** CD39 frequency in HC $CD8^+$ T cells activated with αCD3/αCD28 ± indicated cytokine in the absence (red) or presence (blue) of STATTIC. **E-F**: data are pooled from > 5 independent experiments; each experiment performed in a single donor with each data point representing the average of 2-3 technical replicates. Data represent mean ± SEM and *$P \leq 0.05$ **$P \leq 0.01$ by Paired T-Test.

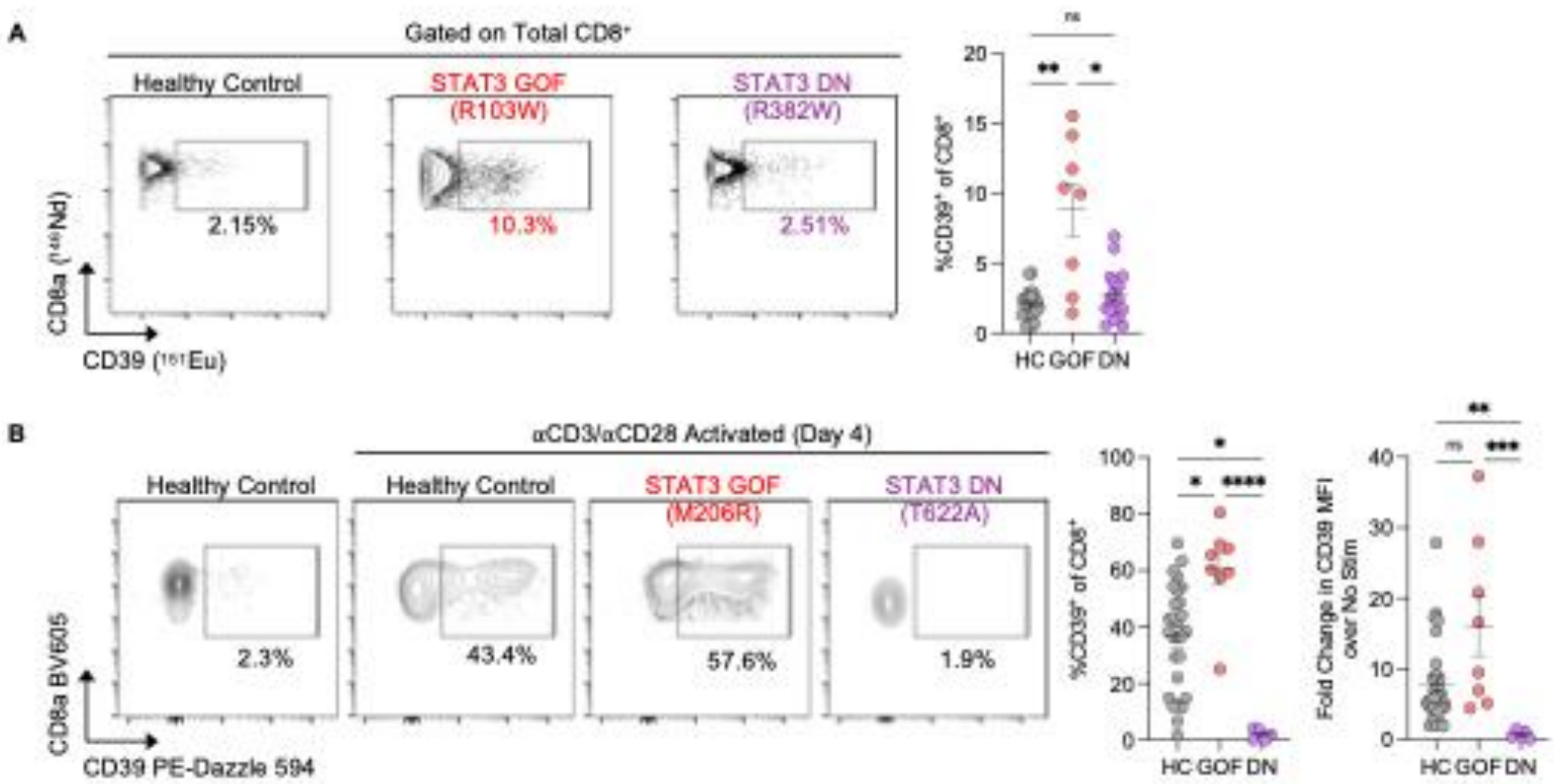

Supplemental Figure 11: CD39 induction in patients bearing STAT3 GOF or DN variants. **(A)** Representative mass cytometry CD39 frequency plots from a HC, patient with STAT3 GOF, and a patient with STAT3 DN, followed by quantification from multiple individuals. Healthy control and STAT3 GOF values are also used in Supplemental Figure 5A. **(B)** On the left, representative CD39 induction in a STAT3 GOF and STAT3 DN patient compared to HC after 4 days of activation with αCD3/αCD28. On the right, quantification of CD39 frequency and fold change in expression from HCs ($n = 28$), STAT3 GOF ($n = 8$) and STAT3 DN ($n = 5$) patients. For Supplemental Figure 11A: $CD39^+$ frequency values from HC and untreated patients with STAT3 GOF were also used in Supplemental Figure 5A. For **A**: data are pooled from 3 independent experiments. For **B**: data are from 1 experiment. Data represent mean ± SEM and *$P \leq 0.05$, **$P \leq 0.01$, ***$P \leq 0.001$, ***$P \leq 0.0001$ by Kruskal-Wallis test with Dunn's multiple comparison test.

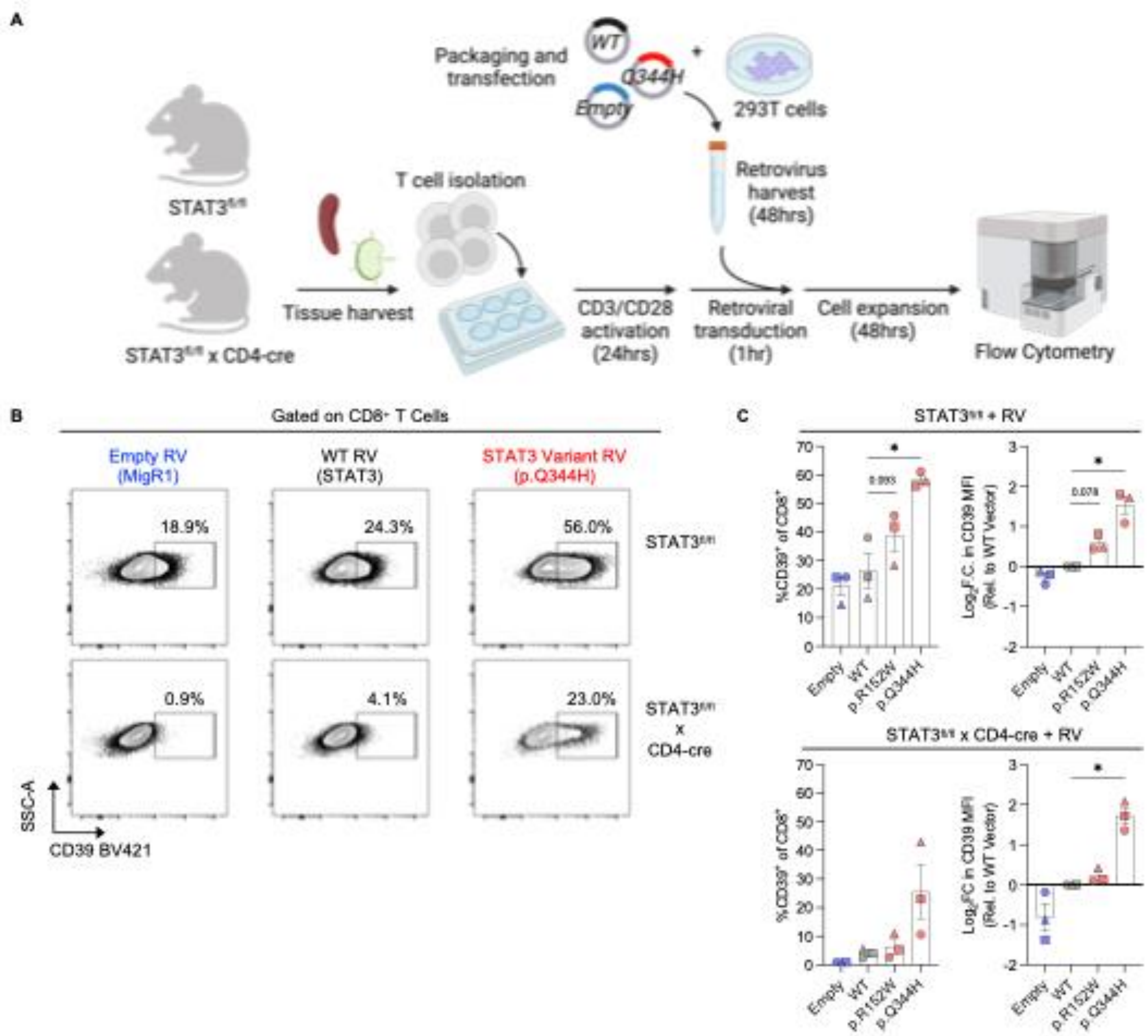

Supplemental Figure 12: CD8⁺ T cells bearing a STAT3 GOF variant induce higher levels of CD39 upon T cell receptor engagement in humans and mice. (A) Introduction of STAT3 GOF variants into STAT3$^{fl/fl}$ (control) or STAT3$^{fl/fl}$ x CD4-cre (experimental) murine T cells using a retroviral transduction system with Empty (MigR1), WT STAT3, or a STAT3 GOF variants (R152W or Q344H). **(B)** Representative CD39 flow cytometry plot in CD8$^+$ T cells in control and test mice across retroviral transduction conditions. **(C)** Quantification (frequency and relative expression) of CD39$^+$CD8$^+$ T cells from STAT3$^{fl/fl}$ (top) or STAT3$^{fl/fl}$ x CD4-cre (bottom) mice (n = 3). Data are pooled from 3 independent experiments as indicated by symbol shape. Where asterisks are used: Data represent mean ± SEM and *$P \leq 0.05$ by Kruskal-Wallis test with Dunn's

multiple comparison test. Supplemental Figure 12A was created in BioRender (https://BioRender.com/n88d277).

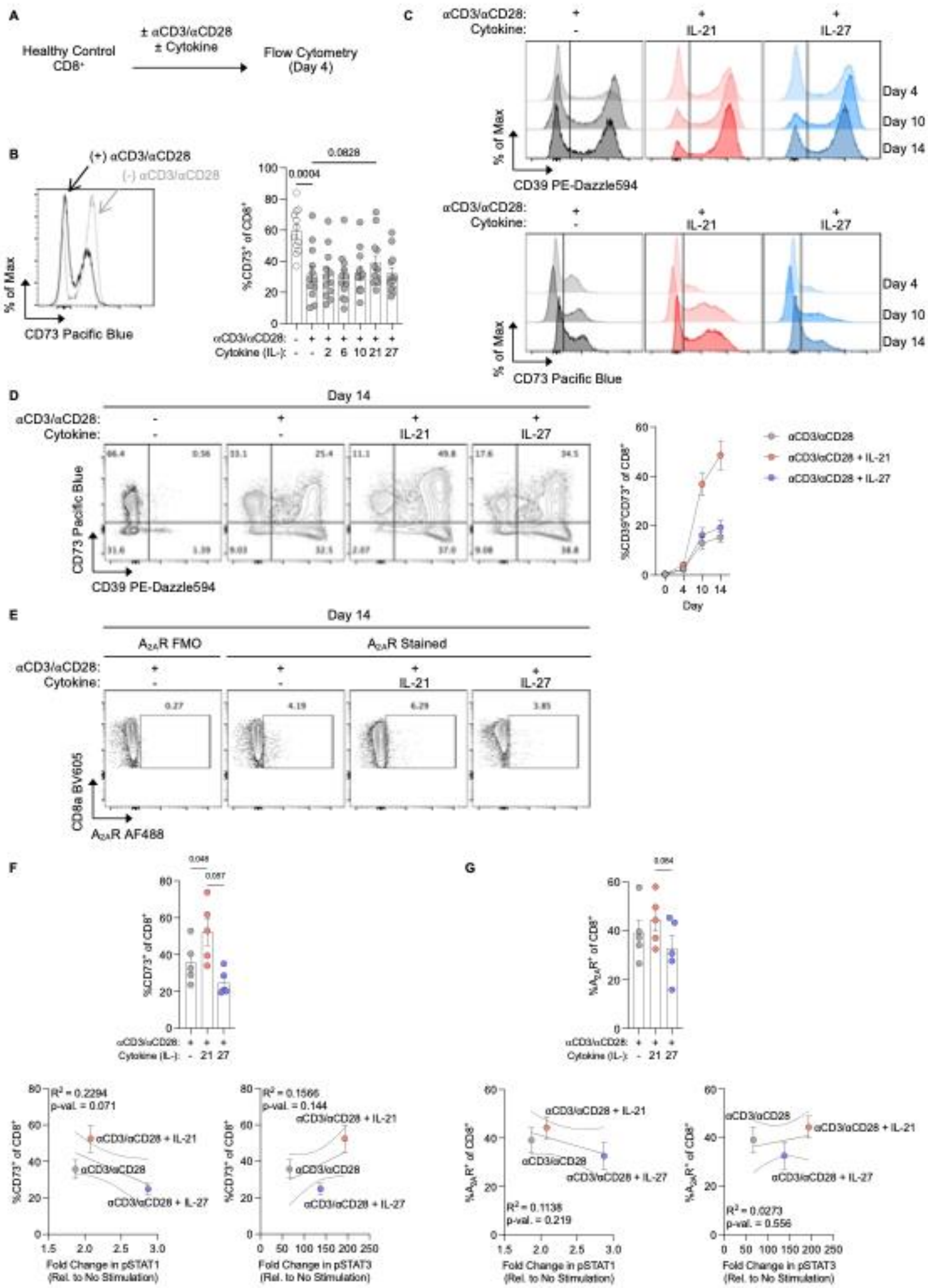

A
Healthy Control CD8+
± αCD3/αCD28
± Cytokine
Flow Cytometry (Day 4)
B
(+) αCD3/αCD28
(-) αCD3/αCD28
% of Max
CD73 Pacific Blue
%CD73+ of CD8+
0.0004
0.0828
αCD3/αCD28:
Cytokine (IL-):
C
αCD3/αCD28:
Cytokine:
IL-21
IL-27
Day 4
Day 10
Day 14
CD39 PE-Dazzle594
CD73 Pacific Blue
D
Day 14
%CD39+CD73+ of CD8+
Day
αCD3/αCD28
αCD3/αCD28 + IL-21
αCD3/αCD28 + IL-27
E
A2AR FMO
A2AR Stained
CD8a BV605
A2AR AF488
F
G
Fold Change in pSTAT1 (Rel. to No Stimulation)
Fold Change in pSTAT3 (Rel. to No Stimulation)

Supplemental Figure 13: Role of STAT3 in regulating CD73 and $A_{2A}R$ expression on $CD8^+$ T cells. (A) Assay schematic for 4-day activation of HC $CD8^+$ with αCD3/αCD28 ± cytokines. **(B)** Quantification of CD73 frequency on $CD8^+$ T cells after 4 days of activation. **(C)** Representative histograms demonstrating CD39 (top) and CD73 (bottom) expression on $CD8^+$ T cells activated with αCD3/αCD28 ± IL-21 or IL-27 over 14 days. **(D)** Representative bivariate plots of CD39 and CD73 co-expression on $CD8^+$ T cells activated with αCD3/αCD28 ± IL-21 or IL-27 at day 14 and quantification on $CD8^+$ T cells over 14 days. **(E)** Representative bivariate plots of $A_{2A}R$ induction at Day 14. **(F)**: Frequency of $CD73^+CD8^+$ T cells from HC (n = 5) after 10 days of activation with αCD3/αCD28 ± IL-21 or IL-27 (top row), followed by correlation between $CD73^+CD8^+$ T cells and fold change in pSTAT1 and pSTAT3 (bottom row). **(G)**: Frequency of $A_{2A}R^+CD8^+$ T cells from HC (n = 5) after 10 days of activation with αCD3/αCD28 ± IL-21 or IL-27 (top row), followed by correlation between $A_{2A}R^+CD8^+$ T cells and fold change in pSTAT1 and pSTAT3 (bottom row). Data represent mean ± SEM; statistics was calculated by RM One-Way ANOVA with Dunnet's or Tukey's multiple comparison test or simple linear regression with 95% confidence interval indicated. Not listed or ns was not statistically significant.

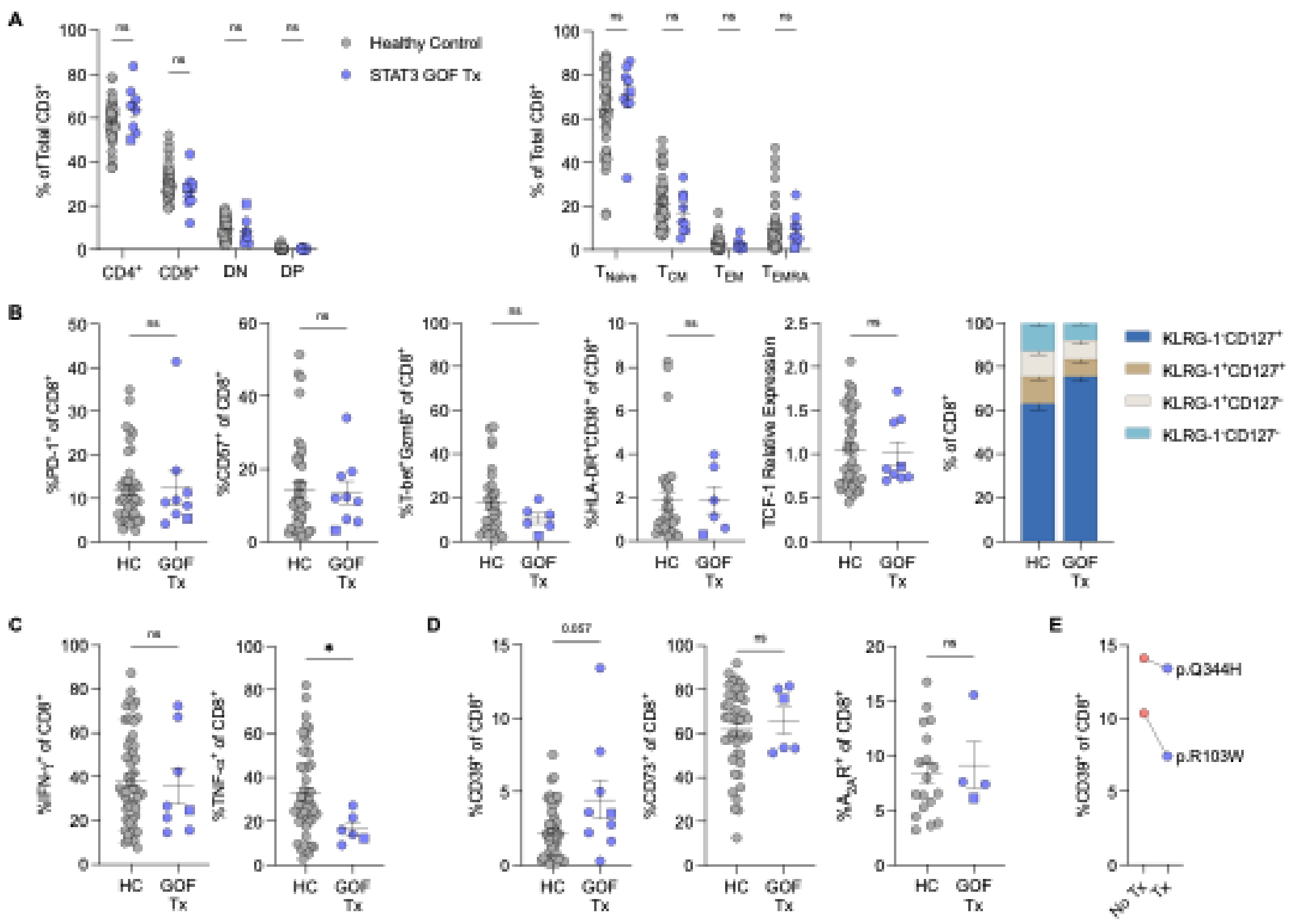

Supplemental Figure 14: Patients with STAT3 GOF on immunomodulatory therapy (JAKi ± αIL-6R) demonstrate partial normalization of aberrant phenotype and function. (A) Comparison of $CD4^+$, $CD8^+$, DN, and DP cells and $CD8^+$ T cell differentiation between HC and treated patients with STAT3 GOF. **(B)** Impact of treatment on $CD8^+$ T cell activation markers treated patients with STAT3 GOF. **(C)** Impact of treatment on IFN-γ and TNF-α cytokine production by $CD8^+$ T cells. **(D)** Impact of treatment on CD39, CD73, and $A_{2A}R$ frequencies within $CD8^+$ T cells. For all plots A-D, at least 18 HC and 4 treated patients with STAT3 GOF were tested. Values for treated patients with STAT3 GOF were also used in Figure 4, B-F and Supplemental Figure 6A and 6E. **(E)** Frequency of $CD39^+CD8^+$ T cells from CyTOF studies in two patients with STAT3 GOF (p.R103W and p.Q344H) before (red) and after (blue) treatment with JAKi ± αIL-6R. Data represent mean ± SEM. $*P \leq 0.05$ by Mann-Whitney Test.

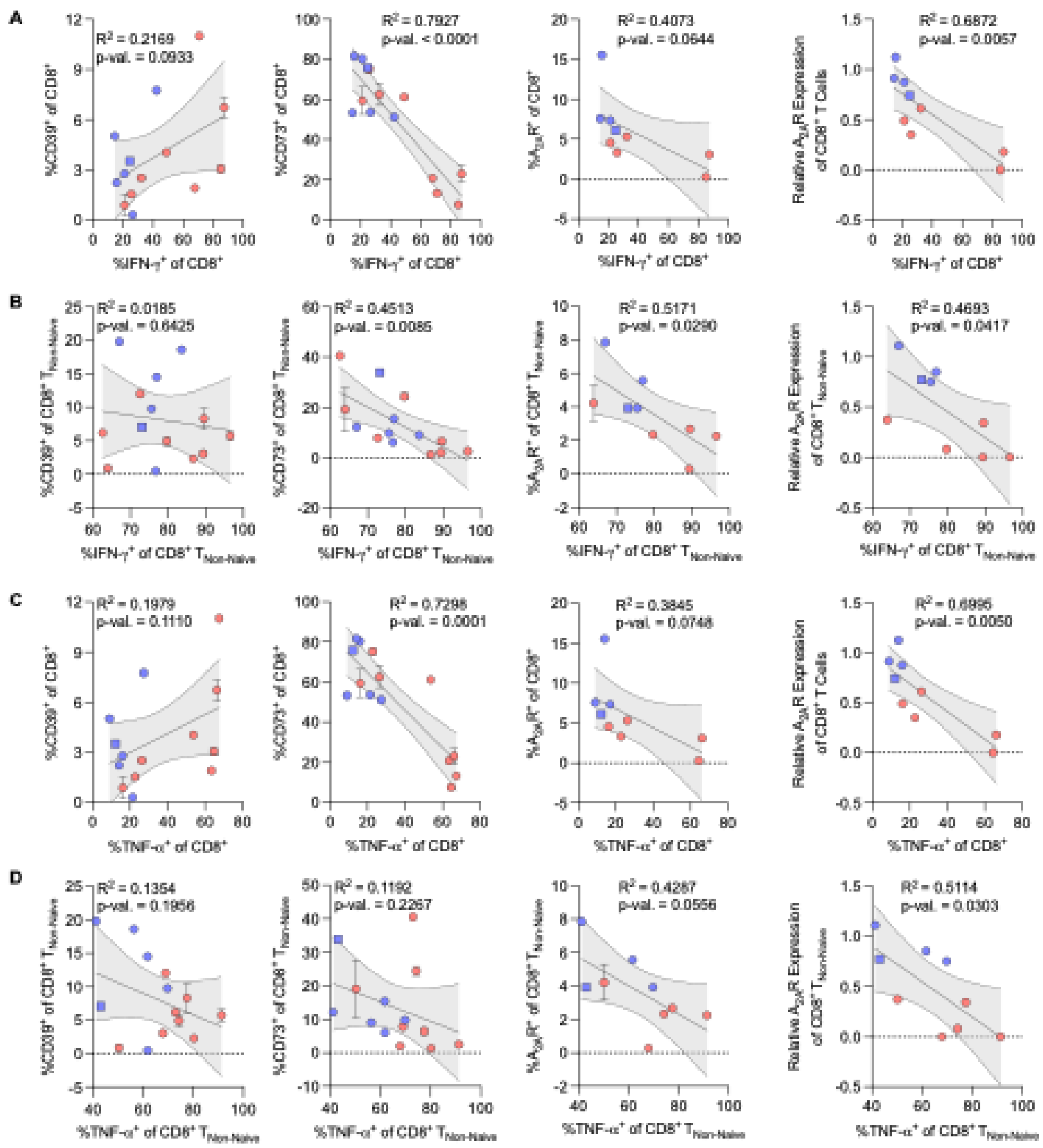

Supplemental Figure 15: Additional correlations between purinergic pathway members and cytokine production by $CD8^+$ T cell subsets. Correlation between CD39, CD73, or $A_{2A}R$ frequencies, or relative $A_{2A}R$ expression, and IFN-γ production in **(A)** total and **(B)** non-naïve $CD8^+$ T cells. Correlation between CD39, CD73, or $A_{2A}R$ frequencies, or relative $A_{2A}R$ expression and TNF-α production in **(C)** total and **(D)** non-naïve $CD8^+$ T cells. Blue square represents patient treated with rapamycin rather than JAKi (blue circles). Significance was calculated via simple linear regression with shaded area indicating 95% confidence interval.

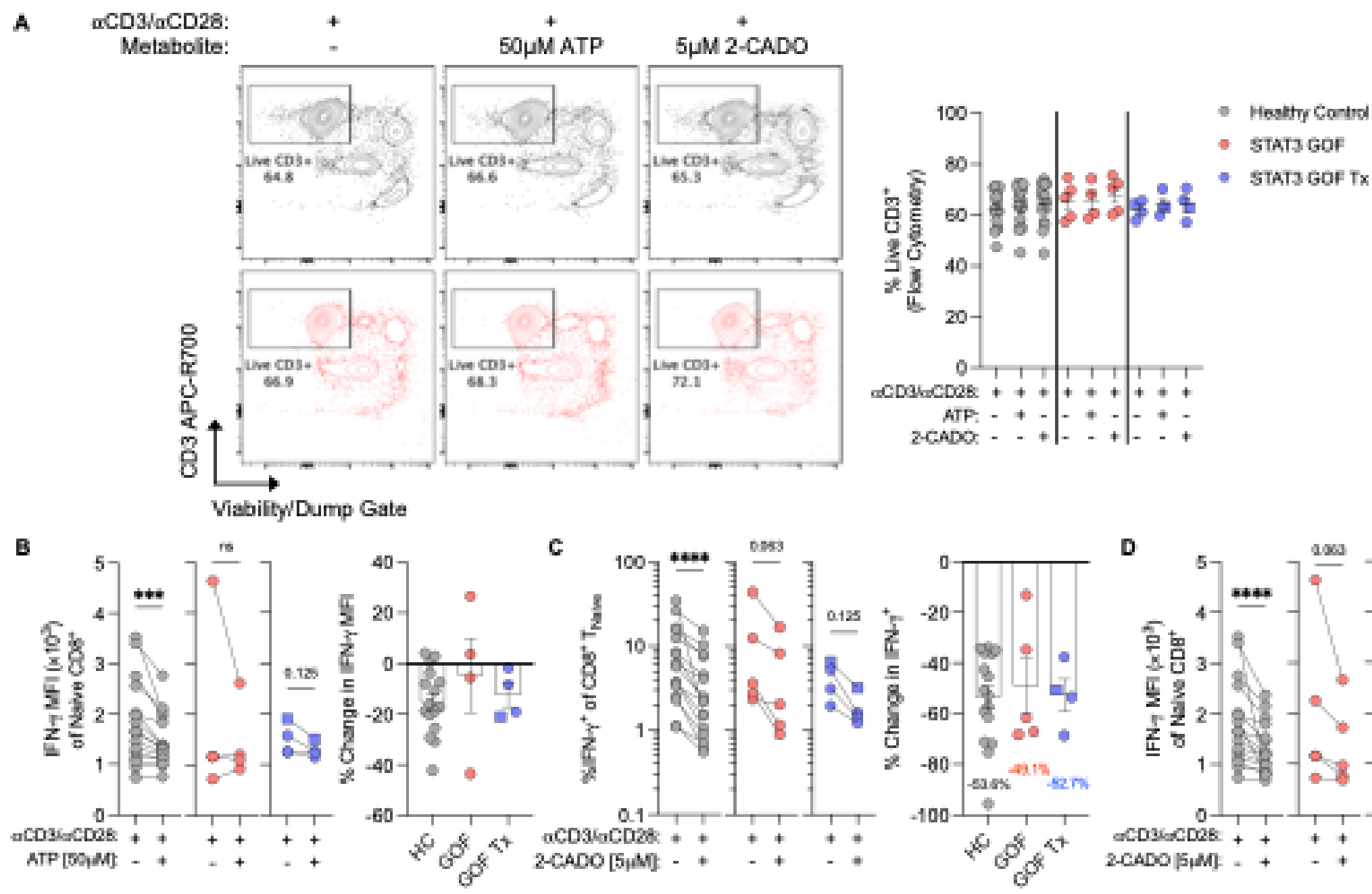

Supplemental Figure 16: Engagement of purinergic pathway results in inhibition of CD8⁺ T cell function. (A) HC and patient cell viability after 24 hours activation with αCD3/αCD28 ± 50μM ATP or 5μM 2-CADO in vitro. **(B)** Paired analysis of naïve CD8⁺ T cell IFN-γ MFI after activation with αCD3/αCD28 ± 50μM ATP followed by quantification of percent change in IFN-γ MFI. Paired analysis of **(C)** %IFN-γ⁺ and **(D)** IFN-γ MFI within naïve CD8⁺ T cells after activation with αCD3/αCD28 ± 5μM 2-CADO and quantification of percent change in IFN-γ⁺ frequency and MFI. For **A**: Data are pooled from 2 independent experiments with 3 repeat HC and 1 patient with STAT3 GOF run in each experiment; repeated samples were averaged into one data point per participant for a total of n = 16 HC (gray), n = 4 or 5 untreated (red), and n = 4 treated (blue) patients with STAT3 GOF. Blue square represents patient treated with rapamycin rather than JAKi. %IFN-γ⁺ values for HC, untreated, and treated patients with STAT3 GOF with αCD3/αCD28 were also used in Figure 5H; IFN-γ MFI values with αCD3/αCD28 in Supplemental Figure 16B were used in Supplemental Figure 16D. Data represent mean ± SEM

and ***$P \leq 0.001$, ****$P \leq 0.0001$ by Wilcoxon matched pairs signed rank test or Kruskal-Wallis test with Dunn's multiple comparisons test as appropriate.

Table S1. Metadata for patients with STAT3 GOF, STAT3 DN, and HC.

Table S1 is available as an attached supplemental spreadsheet (Excel) due to its size.

Table S2. List of DEGs in conventional CD8$^+$ T cells from human scRNA-seq analysis.

Table S2 is available as an attached supplemental spreadsheet (Excel) due to its size.

Table S3. GSEA result summary in conventional CD8$^+$ T cells.

Upregulated in STAT3 GOF							
**NAME**	**SIZE**	**ES**	**NES**	**NOM p-val**	**FDR q-val**	**FWER p-val**	**RANK AT MAX**
HALLMARK_INTERFERON_GAMMA_RESPONSE	173	0.51208454	2.1617355	0	0	0	3711
HALLMARK_INTERFERON_ALPHA_RESPONSE	93	0.5218289	2.0236654	0	0.000173	0.0004	1391
HALLMARK_G2M_CHECKPOINT	182	0.45364296	1.9362842	0	0.000311	0.001	3499
HALLMARK_ALLOGRAFT_REJECTION	160	0.39055002	1.6409074	0.0003196	0.017706	0.0762	1929
HALLMARK_ANGIOGENESIS	13	0.6443134	1.6300333	0.0146421	0.015815	0.0849	799
HALLMARK_MITOTIC_SPINDLE	183	0.37711966	1.6124222	0	0.016007	0.1024	5350
HALLMARK_IL6_JAK_STAT3_SIGNALING	54	0.44277263	1.5635253	0.0117019	0.023905	0.1711	1069
HALLMARK_INFLAMMATORY_RESPONSE	125	0.37017506	1.5037724	0.0046624	0.037382	0.2862	2545
HALLMARK_E2F_TARGETS	185	0.3257695	1.3908293	0.0125846	0.092994	0.6131	3499
HALLMARK_APOPTOSIS	134	0.30513975	1.2494717	0.0780119	0.266093	0.9527	3090
HALLMARK_TGF_BETA_SIGNALING	48	0.36092505	1.2395236	0.1402816	0.260194	0.9613	3825
HALLMARK_PI3K_AKT_MTOR_SIGNALING	93	0.31946695	1.2382655	0.1093492	0.240469	0.9625	6664
HALLMARK_PROTEIN_SECRETION	90	0.32169664	1.2374315	0.1171448	0.22347	0.9635	5597
HALLMARK_ESTROGEN_RESPONSE_LATE	119	0.3056772	1.2232299	0.1065343	0.23038	0.975	2167
HALLMARK_TNFA_SIGNALING_VIA_NFKB	164	0.2576583	1.0837178	0.2756308	0.530647	1	2204
HALLMARK_P53_PATHWAY	166	0.25319943	1.0669346	0.3049363	0.545587	1	2539
HALLMARK_ESTROGEN_RESPONSE_EARLY	127	0.2555726	1.0390085	0.3664861	0.595937	1	2167
HALLMARK_IL2_STAT5_SIGNALING	161	0.24369316	1.0230992	0.3948685	0.610352	1	2791
HALLMARK_COMPLEMENT	143	0.22850192	0.9450612	0.5934933	0.820208	1	2943
HALLMARK_SPERMATOGENESIS	72	0.24838637	0.9205552	0.6161081	0.856555	1	2950
HALLMARK_UV_RESPONSE_DN	99	0.22630708	0.88524216	0.7169439	0.919609	1	5028
HALLMARK_ANDROGEN_RESPONSE	81	0.2192046	0.8308656	0.8089907	1	1	1698
HALLMARK_HEME_METABOLISM	148	0.19880524	0.82817465	0.8713687	0.977027	1	4780
HALLMARK_WNT_BETA_CATENIN_SIGNALING	35	0.24611123	0.79375875	0.8039423	0.997934	1	1812
HALLMARK_HEDGEHOG_SIGNALING	20	0.2634278	0.74397695	0.8307227	1	1	4265
HALLMARK_UNFOLDED_PROTEIN_RESPONSE	106	0.12970278	0.5126856	1	1	1	4340
HALLMARK_OXIDATIVE_PHOSPHORYLATION	198	0.08511553	0.36575946	1	0.999987	1	13541
**Downregulated in STAT3 GOF**							
**NAME**	**SIZE**	**ES**	**NES**	**NOM p-val**	**FDR q-val**	**FWER p-val**	**RANK AT MAX**
HALLMARK_PANCREAS_BETA_CELLS	13	-0.63277864	-1.6534991	0.0159539	0.074123	0.061	683
HALLMARK_MYC_TARGETS_V2	57	-0.43097544	-1.6006259	0.0066178	0.058798	0.0949	3674
HALLMARK_GLYCOLYSIS	146	-0.33231044	-1.4458131	0.0072633	0.153016	0.3263	3307
HALLMARK_APICAL_SURFACE	31	-0.44156492	-1.4351146	0.0510133	0.124624	0.3482	2240
HALLMARK_KRAS_SIGNALING_DN	81	-0.3524503	-1.4005957	0.0301445	0.131989	0.4349	1738
HALLMARK_UV_RESPONSE_UP	126	-0.30240548	-1.2866288	0.0525353	0.270103	0.7606	3896
HALLMARK_MYOGENESIS	112	-0.29367712	-1.2319508	0.0993727	0.347931	0.8834	2806

HALLMARK_EPITHELIAL_MESENCHYMAL_TRANSITION	91	-0.30347574	-1.2303358	0.100785	0.307982	0.8858	1830
HALLMARK_BILE_ACID_METABOLISM	74	-0.31498793	-1.2269938	0.1236094	0.280457	0.8916	2935
HALLMARK_ADIPOGENESIS	163	-0.2730537	-1.2092015	0.0870483	0.286252	0.9206	2417
HALLMARK_HYPOXIA	142	-0.26434535	-1.1402669	0.1739694	0.414726	0.9846	2963
HALLMARK_CHOLESTEROL_HOMEOSTASIS	63	-0.28694054	-1.0858455	0.3015051	0.532358	0.9983	1369
HALLMARK_XENOBIOTIC_METABOLISM	121	-0.25642326	-1.0829833	0.2683756	0.499942	0.9985	2798
HALLMARK_APICAL_JUNCTION	125	-0.25395516	-1.0806516	0.2744593	0.470623	0.9986	2240
HALLMARK_COAGULATION	63	-0.26736543	-1.0126895	0.4242351	0.64403	1	2085
HALLMARK_MTORC1_SIGNALING	188	-0.22053681	-0.995604	0.4602917	0.661111	1	813
HALLMARK_KRAS_SIGNALING_UP	117	-0.23425283	-0.9905183	0.4742991	0.638213	1	1612
HALLMARK_FATTY_ACID_METABOLISM	123	-0.21259327	-0.9017264	0.7069455	0.883654	1	3506
HALLMARK_PEROXISOME	84	-0.22022115	-0.87832516	0.7291102	0.903865	1	2404
HALLMARK_NOTCH_SIGNALING	22	-0.2841464	-0.85108507	0.6867968	0.925591	1	606
HALLMARK_REACTIVE_OXYGEN_SPECIES_PATHWAY	46	-0.21190755	-0.7510673	0.888783	1	1	3535
HALLMARK_MYC_TARGETS_V1	195	-0.1649641	-0.74635416	0.9906542	0.989005	1	11367
HALLMARK_DNA_REPAIR	144	-0.13774304	-0.5970895	1	0.993725	1	4394

Table S4. Mass cytometry antibodies.

Channel/ Metal	Marker	Clone	Dilution	Surface / ICS	Manufacturer	Catalog #	Purchased/ Conjugated	Conjugation Kit Manufacturer	Conjugation Kit Catalog #
89Y	CD45	HI30	300	Surface	Fluidigm	3089003B	Purchased		
113 In	CD45RO	UCHL1	100	Surface	BD Biosciences	555491	Conjugated	Metal from Penn CyTOF Core	
115 In	CD123	6H6	50	Surface	BioLegend	306002	Conjugated	Metal from Penn CyTOF Core	
139 La	Live/Dead	N/A	300	N/A	Macrocyclics	B-272-50	Conjugated	Sigma Aldrich	203521-25g
141 Pr	CD3	UCHT1	800	ICS	BioLegend	300443	Conjugated	Fluidigm	201141A
142 Nd	CD26	BA 5b	200	Surface	BioLegend	302702	Conjugated	Fluidigm	201142A
143 Nd	CD4	RPA-T4	800	ICS	BioLegend	300502	Conjugated	Fluidigm	201143A
144 Nd	CD11b	ICRF44	150	Surface	Fluidigm	3144001B	Purchased		
145 Nd	CD19	HIB19	100	Surface	BioLegend	302202	Conjugated	Fluidigm	201145A
146 Nd	CD8	RPA-T8	100	ICS	Fluidigm	3146001B	Purchased		
147 Sm	CD14	M5E2	200	Surface	BioLegend	301843	Conjugated	Fluidigm	201147A
148 Nd	CD56	HCD56	150	Surface	BioLegend	318345	Conjugated	Fluidigm	201148A
149 Sm	CD11c	3.9	200	Surface	CyTEK	70-0116-U100	Conjugated	Fluidigm	201149A
150 Nd	FceRI	AER-37 (CRA-1)	150	Surface	Fluidigm	3150027B	Purchased		
151 Eu	CD39	A1	400	Surface	BioLegend	328202	Conjugated	Fluidigm	201151A
152 Sm	Granzyme B	CLB-GB11	800	ICS	Invitrogen	MA110338	Conjugated	Fluidigm	201152A
153 Eu	CD45RA	HI100	150	Surface	Fluidigm	3153001B	Purchased		
154 Sm	NKp46	9E2	100	Surface	BioLegend	331947	Conjugated	Fluidigm	201154A
155 Gd	CD27	L128	200	Surface	Fluidigm	3155001B	Purchased		
156 Gd	Helios	22F6	100	ICS	BioLegend	137202	Conjugated	Fluidigm	201156A
158 Gd	PD-1	EH12.2H7	400	Surface	BioLegend	329902	Conjugated	Fluidigm	201158A
159 Tb	CCR7	G043H7	200	Surface	Fluidigm	3159003A	Purchased		
160 Gd	Tbet	4B10	200	ICS	Fluidigm	3160010B	Purchased		
161 Dy	CTLA-4	BNI3	100	ICS	Fluidigm	3161004B	Purchased		
162 Dy	Foxp3	PCH101	100	ICS	Fluidigm	3162011A	Purchased		
163 Dy	CRTH2	BM16	75	Surface	Fluidigm	3163003B	Purchased		
164 Dy	CD161	HP-3G10	200	Surface	Fluidigm	3164009B	Purchased		
165 Ho	Eomes	WD1928	300	ICS	Invitrogen	14-4877-82	Conjugated	Fluidigm	201165A
166 Er	TCF-1	7F11A10	50	ICS	BioLegend	655202	Conjugated	Fluidigm	201166A
167 Er	CD38	HIT2	200	Surface	Fluidigm	3167001B	Purchased		
168 Er	CD138	DL-101	100	Surface	Fluidigm	3168009B	Purchased		
169 Tm	TIGIT	MBSA43	100	Surface	Invitrogen	16-9500-82	Purchased		
170 Er	CXCR5	RF8B2	50	Surface	BD Biosciences	552032	Conjugated	Fluidigm	201170A
171 Yb	ST2	B4E6	50	Surface	MDBioSciences	101002	Conjugated	Fluidigm	201171A
172 Yb	Ki67	B56	400	ICS	Fluidigm	3172024B	Purchased		

173 Yb	HLA-DR	L243	300	Surface	Fluidigm	3173005B	Purchased		
174 Yb	TCRgd	B1	150	Surface	BioLegend	331202	Conjugated	Fluidigm	201174A
175 Lu	IgD	IA6-2	800	Surface	BioLegend	348235	Conjugated	Fluidigm	201175A
176 Yb	CD127/ IL-7Ra	A019D5	200	Surface	Fluidigm	3176004B	Purchased		
191+193	Iridium	N/A	4000	N/A	Fluidigm	201192B	Purchased		
209 Bi	CD16	3G8	200	Surface	Fluidigm	3209002B	Purchased		

Table S5. Flow cytometry antibodies.

PMA/Ionomycin Cytokine Panel (Human PBMCs)						
**Marker**	**Fluorophore**	**Source**	**Clone**	**Catalog No.**	**Dilution**	**Stain**
Fc Block	N/A	BD Biosciences	N/A	564220	200	Surface
CD4	BUV395	BD Biosciences	RPA-T4	564724	400/400	Surface/Intracellular
CD45RO	BUV805	BD Biosciences	UCHL1	748367	200	Surface
IL-2	BV421	BD Biosciences	5344.111	562914	100	Intracellular
CD14	V500	BD Biosciences	M5E2	561391	300	Surface
CD16	V500	BD Biosciences	3G8	561394	300	Surface
CD19	V500	BD Biosciences	HIB19	561121	300	Surface
Live/Dead	Aqua	Invitrogen/ThermoFisher	N/A	L34996A	300	Surface
CD8a	BV605	Biolegend	RPA-T8	301040	100	Surface
CD45RA	BV650	BD Biosciences	HI100	563963	200	Surface
CD27	BV785	Biolegend	O323	302832	200	Surface
IL-17A	AF488	Biolegend	BL168	512308	100	Intracellular
TNF-α	PerCP/Cy5.5	Biolegend	MAb11	502926	100	Intracellular
IL-13	PE	Biolegend	JES10-5A2	501903	100	Intracellular
CD39	PE-Dazzle594	Biolegend	A1	328224	200	Surface
IFN-γ	PE-Cy7	Biolegend	B27	506518	400	Intracellular
IL-21	AF647	Biolegend	3A3-N2	513006	50	Intracellular
CD3	APC-R700	BD Biosciences	UCHT1	565119	400/400	Surface/Intracellular

Purinergic Molecules Panel						
**Marker**	**Fluorophore**	**Vendor**	**Clone**	**Catalog No.**	**Dilution**	**Stain**
Fc Block	N/A	BD Biosciences	N/A	564220	200	Surface
CD45RA	BUV395	BD Biosciences	H100	740298	200	Surface
Live/Dead	Blue	Invitrogen/ThermoFisher	N/A	L34962A	1000	Surface
CD16	BUV496	BD Biosciences	3G8	612944	200	Surface
CD14	BUV563	BD Biosciences	M5E2	741360	200	Surface
CD45	BUV615	BD Biosciences	HI30	751472	300	Surface
CD11c	BUV661	BD Biosciences	B-ly6	612967	100	Surface
CD56	BUV737	BD Biosciences	NCAM16.2	612766	200	Surface
CD26	BUV805	BD Biosciences	L272	749179	100	Surface
CD19	BV421	BD Biosciences	HIB19	562440	300	Surface
CD73	Pacific Blue	Biolegend	AD2	344012	100	Surface
CD3	BV570	Biolegend	UCHT1	300436	400	Surface
CD8a	BV785	Biolegend	RPA-T8	301046	200	Surface
A2AR	AF488	R&D Systems	599717	FAB94971G	50	Surface

CD20	PerCP	Biolegend	2H7	302324	50	Surface
TCRγδ	PerCP-eF710	Invitrogen/ThermoFisher	B1.1	46-9959-42	200	Surface
CD4	cFluor YG584	CyTEK	SK3	R7-20041	400	Surface/Intracellular
CD25	PE-Cy5	BD Biosciences	M-A251	555433	200	Surface
CD39	PE-Fire810	Biolegend	A1	328245	200	Surface
CD27	APC	Biolegend	O323	302810	100	Surface
CD127	APC-R700	BD Biosciences	HIL-7R-M21	565185	100	Surface
CD38	APC-eFluor780	Invitrogen/ThermoFisher	HIT2	47-0389-42	100	Surface
HLA-DR	APC-Fire810	Biolegend	L243	307674	200	Surface

Control

IgG2A Isotype	AF488	R&D Systems	20102	IC003G	50	Surface

T Cell Activation-Exhaustion Panel (Small)

**Marker**	**Fluorophore**	**Vendor**	**Clone**	**Catalog No.**	**Dilution**	**Stain**
Fc Block	N/A	BD Biosciences	N/A	564220	200	Surface
TIM-3	BV421	Biolegend	F38-2E2	345008	100	Surface
CD69	PacBlue	Biolegend	FN50	310920	200	Surface
CD14	V500	BD Biosciences	M5E2	561391	300	Surface
CD16	V500	BD Biosciences	3G8	561394	300	Surface
CD19	V500	BD Biosciences	HIB19	561121	300	Surface
Live/Dead	Aqua	Invitrogen/ThermoFisher	N/A	L34996A	300	Surface
CD8a	BV605	Biolegend	RPA-T8	301040	200	Surface
CD45RA	BV650	BD Biosciences	H100	563963	200	Surface
CD27	BV785	Biolegend	O323	302832	200	Surface
CD4	AF488	BD Biosciences	RPA-T4	557695	200	Surface
TOX	PE	Miltenyi	REA473	130-120-716	200	Intracellular
CD39	PE/Dazzle 594	Biolegend	A1	328224	200	Surface
CD25	PE-Cy5	BD Biosciences	M-A251	555433	200	Surface
LAG-3	PE-Cy7	Biolegend	11C3C65	369310	200	Surface
TCF1/TCF7	AF647	Cell Signaling Technologies	C63D9	6709S	200	Intracellular
CD3	APC-R700	BD Biosciences	UCHT1	565119	100	Surface
PD-1	APC-Fire750	Biolegend	EH12.2H7	329954	100	Surface

Additional Markers and Alternatives for Small T Cell Activation-Exhaustion Panel

CD4	BUV395	BD Biosciences	RPA-T4	564724	200	Surface
CD45RO	BUV805	BD Biosciences	UCHL1	748367	200	Surface
CD57	eFluor450	Invitrogen/ThermoFisher	TB01	48-0577-42	200	Surface
CD73	Pacific Blue	Biolegend	AD2	344012	100	Surface
CD25	FITC	Biolegend	M-A251	356106	200	Surface
CD73	FITC	Biolegend	AD2	344016	100	Surface
CTLA-4	PE-Cy5	BD Biosciences	BNI3	555854	50	Intracellular

T Cell Activation-Exhaustion Panel (Large)						
**Marker**	**Fluorophore**	**Vendor**	**Clone**	**Catalog No.**	**Dilution**	**Stain**
Fc Block	N/A	BD Biosciences	N/A	564220	200	Surface
Ki67	BUV395	BD Biosciences	B56	564071	300	Intracellular
Live/Dead	Live/Dead Blue	Invitrogen/ThermoFisher	N/A	L34962A	1000	Surface
CD4	BUV496	BD Biosciences	RPA-T4	741134	400/400	Surface/Intracellular
CD127	BUV563	BD Biosciences	HIL-7R-M21	748489	100	Surface
CD27	BUV737	BD Biosciences	O323	751681	400	Surface
CD49D	BUV805	BD Biosciences	9F10	749454	300	Surface
PD-1	BV421	Biolegend	EH12.2H7	329920	100	Surface
CD57	eFluor 450	Invitrogen/ThermoFisher	TB01	48-0577-42	200	Surface
CD69	BV480	BD Biosciences	FN50	747519	200	Surface
Granzyme B	BV510	BD Biosciences	GB11	563388	300	Intracellular
HLA-DR	BV570	Biolegend	L243	307638	150	Surface
CD8a	BV605	Biolegend	RPA-T8	301040	100	Surface
CD45RA	BV650	BD Biosciences	H100	563963	200	Surface
LAG-3	BV711	Biolegend	11C3C65	369320	100	Surface
CCR7	BV750	Biolegend	G043H7	353254	100	Surface
Tbet	BV785	Biolegend	4B10	644835	300	Intracellular
CD73	FITC	Biolegend	AD2	344016	100	Surface
KLRG1	PerCP-Cy5.5	Biolegend	SA231A2	367708	100	Surface
CD38	PerCP-efluor 710	Invitrogen/ThermoFisher	HIT2	46-0389-42	150	Surface
TOX	PE	Miltenyi	REA473	130-120-716	200	Intracellular
CD39	PE-Dazzle 594	Biolegend	A1	328224	200	Surface
CTLA-4	PE-Cy5	BD Biosciences	BNI3	555854	200	Intracellular
NKG2D	PE-Cy7	Biolegend	1D11	320812	200	Surface
TCF1	Alexa Fluor 647	Cell Signaling Technologies	C63D9	6709S	200	Intracellular
CD3	APC-R700	BD Biosciences	UCHT1	565119	200	Surface
EOMES	APC-eFluor 780	Invitrogen/ThermoFisher	WD1928	47-4877-42	100	Intracellular

Additional Markers and Alternatives for T Cell Activation-Exhaustion Panel (Large)

Helios	PE-Cy7	Invitrogen/ThermoFisher	22F6	25-9883-42	200	Intracellular

Single Cell RNA-Sequencing Panel for Sorting						
**Marker**	**Fluorophore**	**Vendor**	**Clone**	**Catalog No.**	**Dilution**	**Stain**
CD14	V500	BD Biosciences	M5E2	561391	300	Surface
CD16	V500	BD Biosciences	3G8	561394	300	Surface
CD19	V500	BD Biosciences	HIB19	561121	300	Surface
Live/Dead	Aqua	Invitrogen/ThermoFisher	N/A	L34996A	300	Surface
CD8a	BV605	Biolegend	RPA-T8	301040	200	Surface

CD4	AF488	BD Biosciences	RPA-T4	557695	200	Surface
CD3	APC-R700	BD Biosciences	UCHT1	565119	100	Surface

Phospho-Flow Panel Antibodies						
**Marker**	**Fluorophore**	**Vendor**	**Clone**	**Catalog No.**	**Dilution**	**Stain**
Fc Block	N/A	BD Biosciences	N/A	564220	200	Surface
CD4	BUV395	BD Biosciences	RPA-T4	564724	200	Surface
CD45RO	BUV805	BD Biosciences	UCHL1	748367	200	Surface
pSTAT6 (Y641)	V450	BD Biosciences	18/P-Stat6	561203	50	Intracellular
CD14	V500	BD Biosciences	M5E2	561391	300	Surface
CD16	V500	BD Biosciences	3G8	561394	300	Surface
CD19	V500	BD Biosciences	HIB19	561121	300	Surface
Live/Dead	Aqua	Invitrogen/ThermoFisher	N/A	L34996A	300	Surface
CD8a	BV605	Biolegend	RPA-T8	301040	200	Surface
CD45RA	BV650	BD Biosciences	H100	563963	200	Surface
CD27	BV785	Biolegend	O323	302832	200	Surface
pSTAT1 (Y701)	AF488	BD Biosciences	4a	612596	50	Intracellular
pSTAT5 (Y694)	RB705	BD Biosciences	47/Stat5(pY694)	570286	100	Intracellular
pSTAT3 (Y705)	PE	BD Biosciences	4/P-STAT3	612569	50	Intracellular
CD39	PE-Cy7	Biolegend	A1	328212	200	Surface
Total STAT3	APC	BD Biosciences	M59-50	560392	50	Intracellular
CD3	APC-R700	BD Biosciences	UCHT1	565119	200	Surface

Additional Markers and Alternatives

pSTAT1 (Y701)	BV421	BD Biosciences	4a	562985	50	Intracellular
CD73	FITC	Biolegend	AD2	344016	100	Surface

Other Commonly Used Human Antibodies						
**Marker**	**Fluorophore**	**Vendor**	**Clone**	**Catalog No.**	**Dilution**	**Stain**
CD126 (IL-6Ra)	BV421	BD Biosciences	M5	564163	50	Surface
CD130 (gp130)	PE	BD Biosciences	AM64	555757	50	Surface
pSTAT5 (Y694)	PE-Cy7	BD Biosciences	47/Stat5(pY694)	560117	50	Intracellular

Mouse T Cell Activation/Exhaustion Panel						
**Marker**	**Fluorophore**	**Vendor**	**Clone**	**Catalog No.**	**Dilution**	**Stain**
CD16/CD32 (FcBlock)	N/A	BD Pharmigen	2.4G2	553142	300	Surface
CD44	BUV395	BD OptiBuild	IM7	740215	400	Surface
B220/CD45R	BUV496	BD Horizon	RA3-6B2	612950	150	Surface
CD4	BUV563	BD OptiBuild	GK1.5	741083	800	Surface
CD127	BUV737	BD Horizon	SB/199	612841	100	Surface
CD8a	BUV805	BD Horizon	53-6.7	612898	1800	Surface

Granzyme B	BV421	BD Horizon	GB11	563389	100	Intracellular
Ly108	Pacific Blue	BioLegend	330-AJ	134608	100	Surface
CD69	BV480	BD OptiBuild	H1.2F3	746813	200	Surface
CD3	BV510	BD OptiBuild	17A2	740147	100	Surface
CD45	BV570	BioLegend	30-F11	103136	200	Surface
TIM-3	BV605	BioLegend	RMT3-23	119721	100	Surface
KLRG-1	BV650	BD OptiBuild	2F1	740553	100	Surface
CD39	SB702	Invitrogen	24DMS1	67-0391-82	100	Surface
T-bet	BV786	BD Horizon	O4-46	56141	25	Intracellular
CD73	FITC	Biolegend	Ty/11.8	127220	100	Surface
CXCR5 (CD185)	BB700	BD Horizon	2G8	566493	50	Surface
TCF	PE	BD Pharmingen	S33-966	564217	100	Intracellular
CD62L	PE-CF594	BD Horizon	MEL-14	562404	800	Surface
Ly6C	PE-Cy5.5	Invitrogen	RB6-8C5	35-5931-80	400	Surface
Eomes	PE-Cy7	Invitrogen	Dan11mag	25-4875-82	200	Intracellular
TOX	APC	Miltenyi Biotec	REA473	130-118-335	100	Intracellular
Ki-67	AF700	BD Pharmingen	B56	561277	3000	Intracellular
Zombie NIR	Viability	BioLegend	N/A	423106	3000	Surface
PD-1	APC-Fire750	BioLegend	29F.1A12	135239	600	Surface

Mouse Intracellular Cytokine Detection						
**Marker**	**Fluorophore**	**Vendor**	**Clone**	**Catalog No.**	**Dilution**	**Stain**
CD16/CD32 (FcBlock)	N/A	BD Pharmigen	2.4G2	553142	300	Surface
CD44	BUV395	BD OptiBuild	IM7	740215	400	Surface
CD4	BUV563	BD OptiBuild	GK1.5	741083	1600	Surface/Intracellular
IL-17A	BUV737	Invitrogen	eBio17B7	367-7177-82	50	Intracellular
CD8a	BUV805	BD Horizon	53-6.7	612898	3600	Surface/Intracellular
Granzyme B	BV421	BD Horizon	GB11	563389	100	Intracellular
RORgt	BV480	BD Horizon	Q31-378	567176	100	Intracellular
CD45	BV570	BioLegend	30-F11	103136	200	Surface
IFN-γ	BV650	BioLegend	XMG1.2	505832	100	Intracellular
CD39	SB702	Invitrogen	24DMS1	67-0391-82	100	Surface
T-bet	BV786	BD Horizon	O4-46	56141	25	Intracellular
CD62L	BB515	BD Horizon	MEL-14	565261	200	Surface
CD19	Biotinylated	BioLegend	6D5	115503	600	Surface
CD11b	Biotinylated	BioLegend	M1/70	101203	600	Surface
CD11c	Biotinylated	BioLegend	N418	117303	600	Surface
NK-1.1	Biotinylated	BioLegend	PK136	108703	600	Surface
Gr-1 (Ly-6G/Ly-6C)	Biotinylated	BioLegend	RB6-8C5	108403	600	Surface
Ep-Cam	Biotinylated	BioLegend	G8.8	118203	600	Surface

Streptavidin	PerCP	BioLegend	NA	405213	900	Surface
IL-2	PE	BioLegend	JES6-5H4	503808	200	Intracellular
IL-13	eFluor610	Invitrogen	eBio13A	61-7133-82	250	Intracellular
CD3	PE-Cy7	BioLegend	17A2	100220	400	Surface/Intracellular
TNF-α	AF647	BioLegend	MP6-XT22	506314	100	Intracellular
Zombie NIR	Viability	BioLegend	N/A	423106	3000	Surface
CXCR3 (CD183)	APC-Fire750	BioLegend	CXCR3-173	126539	150	Surface

Table S6. Key reagents/resources.

Buffers/Media	Source	Catalog No.
DPBS (1X) without Ca++ & Mg++	Penn Cell Center	MT21-031-CM
Corning® 500 mL RPMI 1640	Corning	10-040-CV
GemCell™ U.S. Origin Fetal Bovine Serum	GeminiBio	100-500
PENICILLIN STREPTOMYCIN SOL	Penn Cell Center	15140122
L-Glutamine - 200mM (100X)	Penn Cell Center	MT25-005-CI
HEPES Buffer, pH 7.4, 1M, TC tested	Penn Cell Center	1138
Gibco™ MEM Non-Essential Amino Acids Solution (100X)	Fisher Scientific	11-140-050
Sodium Pyruvate - 100X 11g/l 100mM	Penn Cell Center	1175
2-Mercaptoethanol	Sigma-Aldrich	M6250-10ML
eBioscience™ Foxp3 / Transcription Factor Staining Buffer Set	Thermo Scientific	00-5523-00
BD Horizon™ Brilliant Stain Buffer	BD Biosciences	566349
ACK Lysing Buffer	ThermoFisher Scientific	A1049201

Biologics	Source	Catalog No.
BD GolgiStop™ Protein Transport Inhibitor (Containing Monensin)	BD Biosciences	554724
BD GolgiPlug™ Protein Transport Inhibitor (Containing Brefeldin A)	BD Biosciences	555029
Ultra-LEAF™ Purified anti-human CD3 Antibody (Clone = OKT3; 1mg)	Biolegend	317326
Ultra-LEAF™ Purified anti-human CD28 Antibody (Clone = CD28.2; 1mg)	Biolegend	302934
Human IL-2 IS, premium grade	Miltenyi Biotec	130-097-746
Human IL-6 Recombinant Protein, PeproTech®	ThermoFisher Scientific	200-06-100UG
Recombinant Human IL-10 Protein	R&D Systems	217-IL-010
Human IL-21 Recombinant Protein, PeproTech®	ThermoFisher Scientific	200-21-10UG
Recombinant Human IL-27 Protein	R&D Systems	2526-IL-010
Human IFN-α2a, research grade	Miltenyi Biotec	130-093-873
Ultra-LEAF™ Purified anti-mouse CD3ε (Clone = 145-2C11; 1mg)	Biolegend	100340
Ultra-LEAF™ Purified anti-mouse CD28 Antibody (Clone 37.51; 1mg)	Biolegend	102116
InVivoMAb anti-mouse CD3ε (Clone = 145-2C11)	BioXcell	BE0001-1
InVivoMAb anti-mouse CD28 (Clone 37.51)	BioXcell	BE0015-1
Recombinant Mouse IL-27 (NS0-expressed) Protein	R&D Systems	2799-ML-010
InVivoMAb anti-mouse IL-4 (Clone = BE0045)	BioXcell	BE0045
InVivoMAb anti-mouse IFNγ (Clone XMG1.2)	BioXcell	BE0055
Phorbol 12-myristate 13-acetate	Sigma-Aldrich	P1585-1MG
Ionomycin calcium salt	EMD Millipore	I0634-1MG
Adenosine 5′-triphosphate disodium salt hydrate	Sigma-Aldrich	A6419-1G
2-Chloroadenosine	Sigma-Aldrich	C5134-10MG
STAT3 Inhibitor V, Stattic	EMD Millipore	573099-25MG

Additional Flow Cytometry Reagents	Source	Catalog No.
SpectroFlo® QC Beads	Cytek	N7-97355

UltraComp eBeads	ThermoFisher	01-2222-42

**Plasmid Reagent**	**Source**
MigR1K	Krummel Lab
Stat3-203_MigR1K	Genscript
STAT3-203_Q344H_MigR1K	Genscript
pCL_Eco	Abbas Lab

**Prokaryotic Cells**	**Source**	**Catalog No.**
5-alpha Competent E. coli (High Efficiency) DH5a	New England Biolabs	C2987H
SOC Outgrowth Medium	New England Biolabs	B9020S

**Eukaryotic Cell Lines**	**Source**	**Catalog No.**
293T (CRL-3216)	ATCC	CRL-3216

**Other Reagents**	**Source**	**Catalog No.**
Miller Luria Broth (LB):<br>2.5% w/v, autoclaved and supplemented with ampicillin (100ug/ml)	ThermoFisher Scientific	12795027
Lipofectamine 2000	ThermoFisher Scientific	11668027
Dreamtaq Green PCR Master Mix (2x)	ThermoFisher Scientific	K1081

**PCR Primers**	
STAT3 flx (Forward)	5'- GAA GGC AGG TCT CTC TGG TG
STAT3 flx (reverse)	5'- AGG CTG CCA ACA GCC ACT GCC
Cre Primer 1 (Forward)	5'- GCT AAG GAT GAC TCT GGT CA
Cre Primer 2 (Reverse)	5'- CTA ATC GCC ATC TTC CAG CA

75. Lindell RB, et al. Aberrant STAT signaling and T cell dysregulation define a targetable pediatric sepsis endotype. *J Clin Invest*. [published online ahead of print: June 9, 2026]. https://doi.org/10.1172/JCI202867.

76. Zheng GXY, et al. Massively parallel digital transcriptional profiling of single cells. *Nat Commun*. 2017;8(1):14049.

77. Hao Y, et al. Integrated analysis of multimodal single-cell data. *Cell*. 2021;184(13):3573-3587.e29.

78. McGinnis CS, Murrow LM, Gartner ZJ. DoubletFinder: Doublet detection in single-cell RNA sequencing data using artificial nearest neighbors. *Cell Syst*. 2019;8(4):329-337.e4.

79. Robinson MD, McCarthy DJ, Smyth GK. edgeR: a Bioconductor package for differential expression analysis of digital gene expression data. *Bioinformatics*. 2010;26(1):139–140.

80. Wilkinson L. Ggplot2: Elegant graphics for data analysis by WICKHAM, H. *Biometrics*. 2011;67(2):678–679.

81. Borcherding N, Bormann NL, Kraus G. scRepertoire: An R-based toolkit for single-cell immune receptor analysis. *F1000Res*. 2020;9:47.

82. Shugay M, et al. VDJdb: a curated database of T-cell receptor sequences with known antigen specificity. *Nucleic Acids Res*. 2018;46(D1):D419–D427.

83. Tickotsky N, et al. McPAS-TCR: a manually curated catalogue of pathology-associated T cell receptor sequences. *Bioinformatics*. 2017;33(18):2924–2929.

84. Vita R, et al. The Immune Epitope Database (IEDB): 2018 update. *Nucleic Acids Res*. 2019;47(D1):D339–D343.

85. Zhang W, et al. PIRD: Pan Immune Repertoire Database. *Bioinformatics*. 2020;36(3):897–903.

86. Borcherding N, et al. CD4+ T cells exhibit distinct transcriptional phenotypes in the lymph nodes and blood following mRNA vaccination in humans. *Nat Immunol*. 2024;25(9):1731–1741.

87. Naviaux RK, et al. The pCL vector system: rapid production of helper-free, high-titer, recombinant retroviruses. *J Virol*. 1996;70(8):5701–5705.
